# Increasing the bioactive space of peptide macrocycles by thioamide substitution†
†Electronic supplementary information (ESI) available. See DOI: 10.1039/c7sc04671e


**DOI:** 10.1039/c7sc04671e

**Published:** 2018-01-22

**Authors:** Hitesh Verma, Bhavesh Khatri, Sohini Chakraborti, Jayanta Chatterjee

**Affiliations:** a Molecular Biophysics Unit , Indian Institute of Science , Bangalore 560012 , India . Email: jayanta@iisc.ac.in; b NMR Research Centre , Indian Institute of Science , Bangalore 560012 , India

## Abstract

We show that substituting a single atom, O to S (amide to thioamide), in a peptide bond results in global restriction of the conformational flexibility in peptide macrocycles with minimal perturbation of the parent conformation. The van der Waals interactions between the C=S group and the surrounding atoms are the major driving force in inducing the conformational restriction, resulting in well-defined structures of these cyclic peptides with static 3-D presentation of the pharmacophores. Utilizing this property of thioamides, we report the development of a superactive antagonist of pro-angiogenic αvβ3, αvβ5 and α5β1 integrins, which are responsible for cancer cell proliferation and survival. Using simple thio-scanning and spatial screening of a non-efficacious and conformationally flexible cyclic peptide, we could achieve a more than 10^5^ fold enhancement in its efficacy *in cellulo via* a single O to S substitution. The developed peptide shows better efficacy in inhibiting the pro-angiogenic integrins than the drug candidate cilengitide, with a significantly enhanced serum half-life of 36 h compared to that of cilengitide (12 h). The long shelf-life, absence of non-specific toxicity and resistance to degradation of the thioamidated macrocyclic peptides in human serum suggest the promise of thioamides in markedly improving the affinity, efficacy and pharmacology of peptide macrocycles.

## Abstract 2

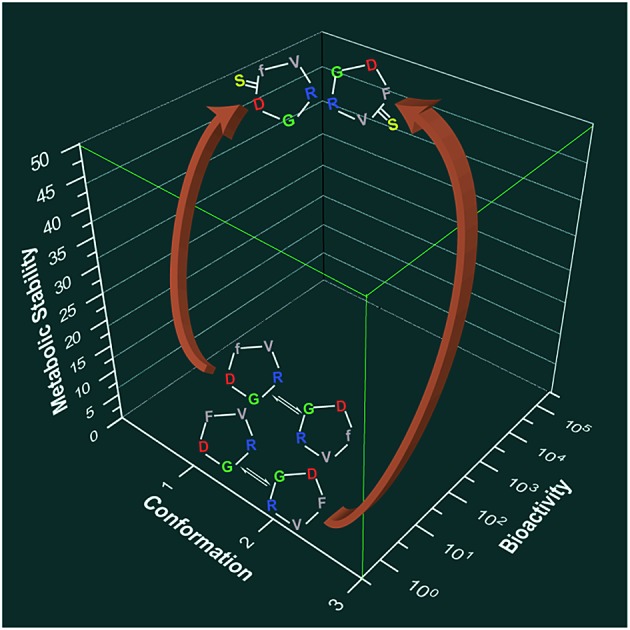
Thioamide substitution into macrocyclic peptides increases the conformational rigidity of the backbone resulting in enhanced biological activity and metabolic stability.

## Introduction

In recent years, peptide macrocycles, as opposed to small molecules, have emerged as an extremely useful scaffold to target various extracellular and intracellular proteins.^1^ The complex structures of peptide macrocycles give rise to a unique combination of *φ*, *ψ*, and *χ* space aiding in molecular recognition towards target binding.^2^ Nevertheless, macrocyclic structures are often associated with conformational flexibility, introducing an entropic penalty to target binding that severely compromises their affinity and selectivity against a biological target.^3^ Thus, covalent or noncovalent constraints that minimize the conformational entropy in peptides are highly desirable.3c,^4^ In particular, constraints that minimally alter the bioactive peptide sequence are of paramount interest. In this context, peptide bond surrogates like peptoids,^5^ retro-inverso peptides,^6^ depsipeptides,^7^ azapeptides^8^ and *N*-methylated peptides^9^ have found use in delivering bioactive analogs with increased affinity, selectivity and metabolic stability. However, the strictest isostere of a peptide bond, the thioamide,^10^ attracted our attention. Thioamides show longer C=S bonds of 1.65 Å compared to 1.23 Å for C=O bonds, and a larger van der Waals radius of S of 1.80 Å compared to 1.52 Å for O in oxoamides.^10^ In addition, the increased rotational barrier about the C–N bond, and the reduced hydrogen bond acceptor but stronger hydrogen bond donor capacity of thioamides compared to regular amides,^10^ encouraged us to understand the conformational behavior of thiosubstituted macrocyclic peptides. Thioamides have found great utility both in biophysics and medicinal chemistry. They have been utilized as minimal fluorescence quenchers, protease sensors, photoswitchable elements in peptides, and probes to interrogate the dynamics of hydrogen bonds in α-helices and β-sheets.^11^ Furthermore, thioamides have also shown improved bioactivity and metabolic stability compared to linear bioactive peptides.^12^ Despite the versatile utility of thioamides, their potential to deliver selective and high-affinity macrocyclic ligands has not been systematically addressed.^13^

Thus, we set out to investigate the role of thioamides in peptide macrocycles, with special emphasis on the effect of thionation in the secondary structure motifs commonly occurring in bioactive macrocycles *viz.* βII, βII′, βVI and γ-turns, as observed in cyclosporine A, gramicidin S, somatostatin agonists, integrin antagonists *etc.*, which have found enormous utility in treating pathological conditions.3*c* This report details the conformational impact of thioamide substitution on cyclic peptide backbones that leads to reduced flexibility of the peptide backbone and allows for the static presentation of the pharmacophores, resulting in a dramatically increased binding affinity and enhanced metabolic stability of the thioamidated macrocyclic peptides.

## Results and discussion

### Conformational impact of thioamides on a cyclic peptide backbone

**Fig. 1:**
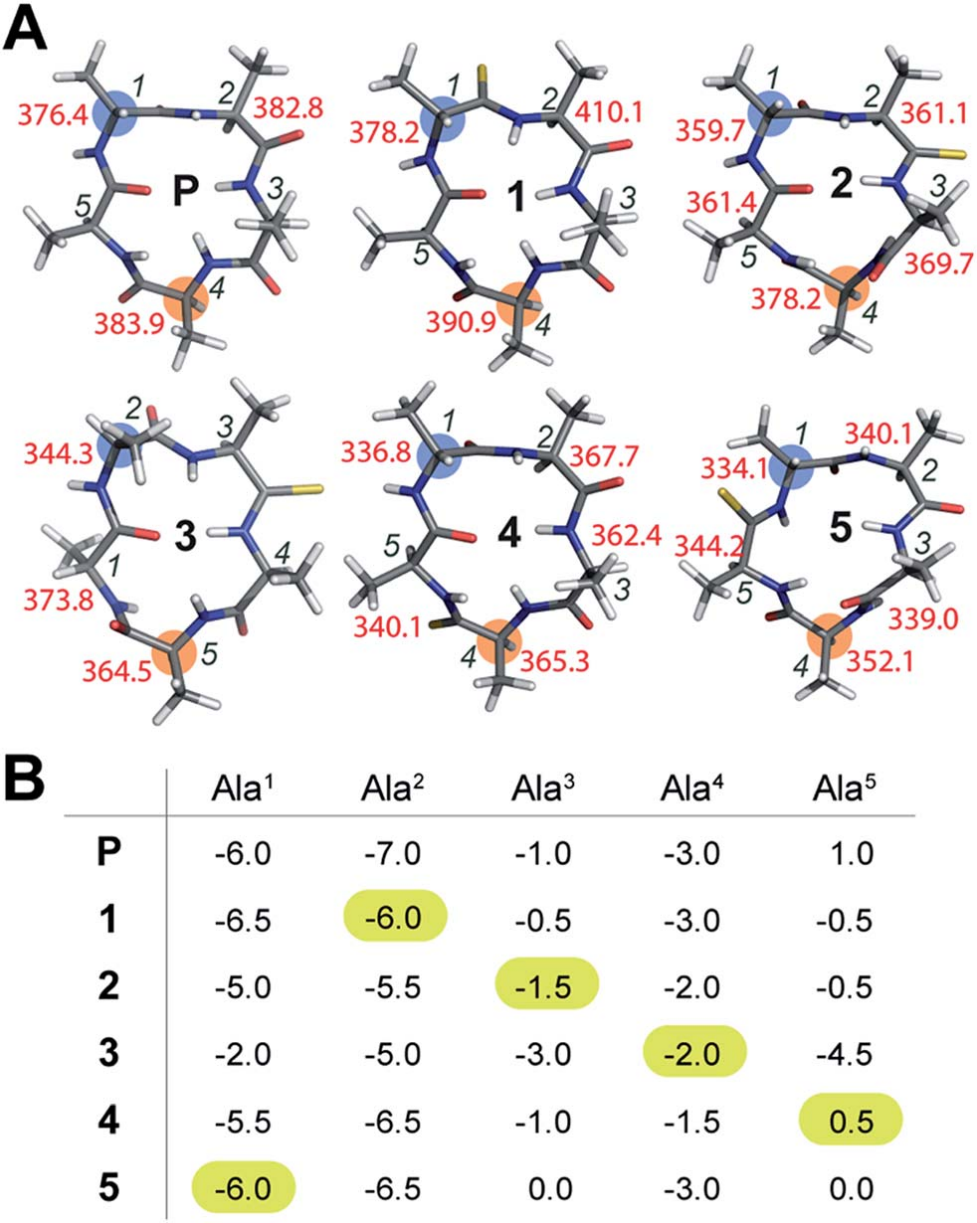
(A) Solution conformation of cyclo(aA_4_) (**P**) and its thio analogs (**1–5**). The *T*_1_ values of the resolved C^α^ are indicated adjacent to the residues. The *i* + 1 residue in the β-turn, and the residue about which the γ-turn is centered, are highlighted. (B) The amide proton temperature coefficients (in ppb K^–1^) determined from the ^1^H NMR spectra acquired between 298 K and 323 K. The value for the thioamide NH is highlighted.

To initially determine the conformational impact of thioamide substitution in peptide macrocycles, we synthesized a regioisomeric library of monothionated analogs of the parent cyclic peptide cyclo(–d-Ala–Ala_4_–), which is known to adopt a βII′-type and γ-type turn (Fig. 1A).^14^ The ^1^H NMR spectra of all the thionated cyclic peptides in DMSO-*d*_6_ were well dispersed indicating the presence of a well-defined conformation (ESI†). Utilizing the distance restraints obtained from the ROESY spectrum, we derived the solution conformations of the cyclic peptides (Fig. 1A). The introduction of the C=S groups did not introduce large perturbation to the global conformations of the cyclic peptides, however, we observed that unlike the C=O groups, none of the thioamides were internally oriented, indicating a lack of intramolecular hydrogen bonding with the C=S groups. The high-resolution structures of the thionated cyclic pentapeptides indicated the coexistence of βII′-type and γ-type turns. The γ-type turn is in equilibrium between an open (indicated by the NOE between the Ala^4^HN–Ala^5^HN protons) and a closed form (suggested by the low temperature coefficient of Ala^5^HN, which is indicative of intramolecular hydrogen bonding (Fig. 1B)) as demonstrated by Kessler and Marshall *et al.*^15^ The thionation of d-Ala^1^, Ala^2^, Ala^4^ and Ala^5^ resulted in cyclic pentapeptides (**1**, **2**, **4** and **5**) with similar global conformations but different *φ* and *ψ* values (ESI†), where the βII′-type turn is centered about d-Ala^1^–Ala^2^ and the γ-turn is centered about Ala^4^.^15^ However, the thionation of Ala^3^ resulted in a conformation with the βII′-type turn centered about Ala^2^–Ala^3^ and the γ-turn centered about Ala^5^ (**3**).

The true externally oriented (solvent exposed) C=O groups in the cyclic pentapeptide are at d-Ala^1^, Ala^2^ and Ala^4^, and upon thionation did not perturb the conformation of the parent peptide. However, thionation at the internally oriented C=O groups, in the case of Ala^3^ (**3**), completely changes the conformation of the peptide; this is also observed for Ala^5^ (**5**), albeit to a lesser extent. These results indicate that thioamide substitution at a site where the C=O group does not engage in intramolecular hydrogen bonding can retain the conformation of the parent macrocyclic peptide. Thus, we note that thioamides can be valuable probes for identifying the hydrogen bond involved in stabilizing the conformation of a macrocycle.11*f* However, we were intrigued by the “frame-shifted” conformation of **3** and wondered whether this was a result of the enhanced hydrogen bond (H-bond) donating properties of the thioamide, as noted by Kessler *et al.*13*d* To understand this behavior, we analysed the temperature coefficients of the amide protons in these peptides (Fig. 1B) along with the ^1^H chemical shifts. The upfield shift of the amide resonances in Ala^3^HN and Ala^5^HN indicate their involvement in intramolecular hydrogen bonding, as opposed to d-Ala^1^HN, Ala^2^HN, and Ala^4^HN in all the peptides except **3** (ESI†).

It was interesting to note that despite the possibility of Ala^2^HN being involved in a γ-turn with Ala^5^CO in **1**,^16^ there was no enhancement in the thioamide H-bond donation and structural alteration. Similarly, the temperature coefficients of the thioamide HN in **2** and **4** did not indicate a significant increase in the thioamide H-bond donation. This suggests that the increased thioamide H-bond donor propensity^17^ is not the driving force for the structural alteration in **3** and **5**, instead, the van der Waals interactions between S (due to the increased size of sulfur and the longer C=S bond) and the surrounding atoms are crucial in dictating the conformation of the peptide, as predicted from the computational studies of thioamides containing dipeptides by Lipton *et al.*11*a* Thus, the internally oriented Ala^3^CO completely flips outward on thio substitution in **3**, presumably due to the 1,2 and 1,3-diaxial strain between S and the flanking Ala^3^ and Ala^4^C^β^. However, owing to the flanking heterochiral residues, thio substitution of Ala^5^CO allows for a partial flip of the thioamide bond.

### Preliminary indication of the structural rigidity in cyclic peptides

To understand whether the thioamide substitution results in reduced flexibility of the cyclic peptide backbone, we determined the ^13^C spin-lattice relaxation time (*T*_1_) of the backbone C^α^ in these cyclic peptides, which provides qualitative information on the segmental motion of amino acids in peptides.^18^ It was intriguing to note that in all the peptides, the *i* + 1 residue in the β-turn displayed the lowest *T*_1_ compared to the other residues in the same macrocycle indicating its segmental rigidity (Fig. 1A) (comparison of the intermolecular *T*_1_ is not feasible due to differences in the concentration and viscosity of the samples).18*a* Subsequently, we compared the *T*_1_ of the *i* + 1 residue with the *T*_1_ of the alanine about which the flexible γ-turn is centered (highlighted in Fig. 1A). Curiously, we observed that this difference in *T*_1_ was smallest in **1** compared to all other thio analogs. Additionally, the difference in *T*_1_ within the residues in **P** was also less than that in the thio analogs, indicating comparable flexibility in the different segments of **P**. This led us to speculate that thioamide substitution presumably assists in increasing the (segmental) structural rigidity in cyclic peptides.

### Evidence of enhanced structural rigidity in aqueous solution

**Fig. 2:**
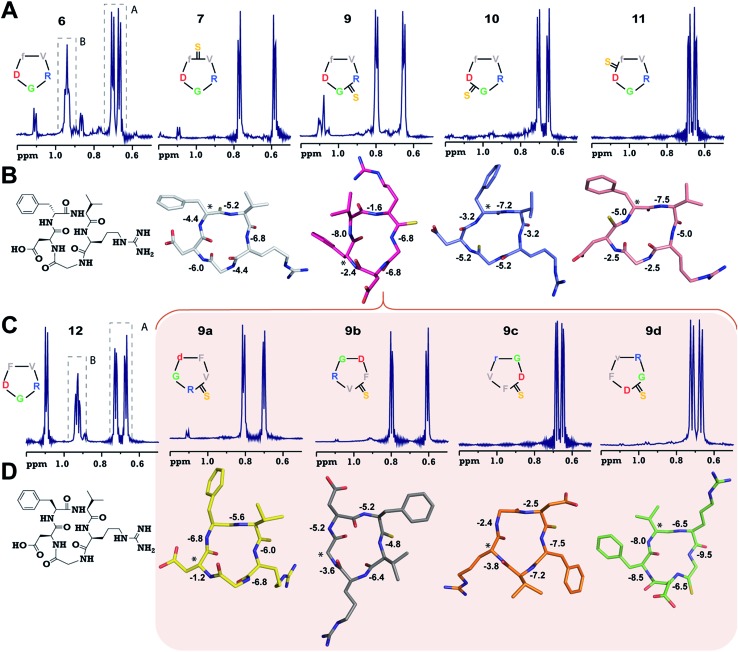
(A) ^1^H NMR spectra of cyclo(RGDfV) (**6**) and its thio-analogs **7–11**. The γ-CH_3_ of Val in both the conformers (A and B) of **6** is shown. (B) The solution structures of **7–11** deduced from 2D ^1^H NMR spectroscopy in aqueous conditions. (C) ^1^H NMR spectra of cyclo(RGDFV) (**12**) showing two conformers. (D) Solution structures of spatial screen analogs of **9** (**9a–9d**). The temperature coefficients of the amide protons (ppb K^–1^) are shown and the asterisk denotes the site of the d-amino acid (Gly in **9b**). Significant resonance overlap precluded the structure determination of **6** and **12**. The hydrogen atoms are omitted for the sake of clarity.

To validate our observation of enhanced structural rigidity in peptide macrocycles through thionation in aqueous solution (due to the hydrogen bonding properties of water, it effectively solvates the intramolecular H-bonds in the peptides, leading to greater flexibility), we resorted to a moderate affinity cyclic peptide integrin antagonist cyclo(RGDfV) (**6**),16*b* which is also known to adopt a βII′-type turn about d-Phe–Val and a γ-turn about Gly as observed in **1**. Interestingly, we noted that **6** displays two conformations in the ratio 1 : 2 on the NMR time scale (Fig. 2A and ESI†) at 25 °C, making it a suitable model to test our hypothesis. Thus, we synthesized a thio-scan library of **6** and assessed the conformations by NMR. Since thioamides undergo sulfur to oxygen exchange under acidic conditions, the orthogonally protected cyclic peptides were initially purified and subjected to global deprotection using an optimized cleavage cocktail of TFA/DCM/TIPS/H_2_O (62.5 : 32.5 : 2.5 : 2.5).^19^ Although we were successful in obtaining all the orthogonally protected thioamidated peptides, c(RGDfV^t^) (**8**) underwent spontaneous acid catalyzed degradation.^19^ To analyze whether the flanking amino acids of the thioamide bond have a role to play in the degradation, we synthesized three additional cyclic peptides with identical chirality but different functional groups: c(RGDfA^t^), c(KGDfV^t^) and c(NleGDfV^t^) (Nle: norleucine). It was surprising to note that all of these protected cyclic peptides underwent spontaneous acid catalyzed degradation as observed in **8** (ESI†). This suggested that the backbone conformation of the peptide has a key role in degradation, rather than the amino acid side chains flanking the thioamide bond. It is worth noting at this point that the model cyclic peptides (**1–5**) did not show any trace of thio to oxo conversion even after 24 months at 25 °C in DMSO solution, indicating the stability of the thiopeptides in an oxidizing environment.

Adding to our excitement, all the thio-variants of **6** displayed a single conformation on the NMR time scale at room temperature, unlike the all-amide **6** (Fig. 2A). Variable temperature ^1^H NMR, recorded from 5 °C to 50 °C, also did not reveal the existence of any minor conformers in slow exchange in **7–11**, (ESI†) directly demonstrating the rigidifying role of the thioamides in the peptide macrocycles, irrespective of the substitution site.

To demonstrate the uniform behavior of the thioamides in dictating the conformation of the cyclic peptide, we analyzed the solution conformations of **7–11** (Fig. 2B). It was encouraging to note that the conformation of these thionated RGD peptides reflects the conformation of the model alanine peptides with identical patterns of thioamide substitution. Furthermore, as observed in **3**, **9** displayed a “frame-shifted” conformation due to the steric bulk associated with the C=S group and not the improved thioamide H-bonding ability.^17^ Despite the involvement of the GlyHN thioamide in a β-type turn in **9**, its temperature coefficient is –6.8 ppb K^–1^, indicating the absence of intramolecular hydrogen bonds, as opposed to d-PheHN (–2.4 ppb K^–1^) and ArgHN (–1.6 ppb K^–1^), which are involved in γ-turns (Fig. 2B). Likewise, the thioamide in **11** (d-PheHN) is also solvent exposed (–5.0 ppb K^–1^) and was not involved in stabilizing the γ-turn about Asp with GlyCO.

We were quite excited to observe that unlike *N*-methylation, which introduces severe conformational heterogeneity into macrocyclic peptides *via cis*–*trans* isomerism (due to the reduced *cis*–*trans* rotational barrier and the pseudoallylic strain between the *N*-methyl and the flanking C^β^ groups within two homochiral residues),^20^ thioamides lower the conformational heterogeneity by reducing the conformational space of the cyclic peptide, which is guided by the van der Waals repulsion between S and the surrounding atoms and the increased *cis*–*trans* rotational barrier of the thioamide bond.^21^ Furthermore, despite structural alteration by thionation of the internally oriented amide bonds (**3** and **9**), the structural rearrangement results in a parent-like conformation, but with an altered side chain identity. These two observations suggested that (i) thioamides are valuable tools to reduce the conformational entropy in peptide macrocycles and (ii) thioamide substitution is an ideal modification for spatial screening^22^ of peptide sequences in search of bioactive conformations.

### Thioamide incorporation yields superactive pro-angiogenic integrin antagonists

To validate our hypothesis and demonstrate the potential of thioamides in delivering selective and high-affinity ligands, we chose to target the cell-adhesion receptors, integrins.^23^ Integrins mediate a diverse set of cellular functions by binding to extracellular matrix components through a set of 24 distinct integrin heterodimers, formed by a combination of 18α-subunits and 8β-subunits. We were keen to simultaneously target the pro-angiogenic αvβ3, αvβ5 and α5β1 integrins, which have partially overlapping ligand specificity and are associated with migration, proliferation and survival in tumor cells.23*b* We sought to develop subtype selective integrin antagonists because: (a) these integrins are validated drug-targets for treating cancer, (b) pre-organization of the pharmacophore (–RGD– sequence) is known to modulate the integrin subtype selectivity, and (c) the *N*-methylated cyclic peptide, cilengitide (**Cilen**), unfortunately failed in phase III clinical trials against glioblastoma.^24^

The inhibitory potency of the peptides to block the association between the extracellular matrix and the integrins overexpressed on cell-surfaces was determined using an *in vitro* competitive solid phase binding assay.^25^ We assessed the affinity of these peptides against the pro-angiogenic αvβ3, αvβ5 and α5β1 integrins, and their selectivity against their αIIbβ3 integrin, which plays an important role in platelet aggregation (Table 1).^26^ The parent peptide, **6**, showed a moderate affinity towards the pro-angiogenic integrins due to the conformational flexibility, which does not allow the static presentation of the pharmacophores responsible for tight binding. On the contrary, the conformationally rigid thio analogs yielded very interesting results. **7**, which can be directly compared with **Cilen**, where an *N*-methylated amide bond is substituted with a thioamide bond, displayed a higher IC_50_ than **6** against αvβ3. However, remarkably it showed a better IC_50_ than **Cilen** against αvβ5 and α5β1 in the *in vitro* assays with purified integrins.

**Table 1:** *In vitro* IC_50_ (nM) values of the peptides against αvβ3, αvβ5, α5β1 and αIIbβ3 integrins determined from competitive ELISAs. The efficacy (*in cellulo* IC_50_ in nM) was determined from the competitive cell-adhesion to vitronectin with αvβ5 expressing MDA-MB-231 breast cancer cells and αvβ3, αvβ5 and α5β1 expressing U-87 MG glioblastoma cells. MCF7 breast cancer cells were taken as negative control in the cell-adhesion assay, where the IC_50_ for all the analogs was in the high μM range (data not shown). *n* = 5 ± SEM

Compound	*In vitro*				*In cellulo*		αvβ3Xαvβ5^*a*^
	αvβ3	αvβ5	α5β1	αIIbβ3	MDA-MB-231	U-87 MG	
**6**	140 ± 95	516 ± 117	164 ± 134	>10^5^	>10^5^	>10^5^	>10^5^
**7**	372 ± 52	0.7 ± 0.2	1.3 ± 0.6	>10^4^	4 ± 2	326 ± 97	260.4
**9**	>10^5^	>10^4^	53 ± 42	>10^4^	>10^5^	>10^5^	>10^5^
**10**	252 ± 200	>10^5^	>10^5^	>10^5^	>10^5^	>10^5^	>10^5^
**11**	72 ± 63	1.3 ± 1	62 ± 45	>10^5^	4.2 ± 4	96 ± 77	93.6
**9a**	169 ± 100	0.5 ± 0.3	23 ± 12	>10^5^	11 ± 9	40 ± 28	84.5
**9b**	0.2 ± 0.1	1.6 ± 0.3	2 ± 0.4	>10^5^	1 ± 0.1	1.6 ± 0.6	0.32
**9c**	>10^5^	>10^4^	461 ± 246	>10^5^	>10^5^	>10^5^	>10^5^
**9d**	584 ± 504	>10^4^	104 ± 95	>10^5^	>10^5^	>10^5^	>10^5^
**12**	166 ± 98	>10^4^	48 ± 20	>10^5^	>10^5^	>10^5^	>10^5^
**Cilen**	0.6 ± 0.4	3 ± 2	6 ± 1	>10^5^	1.2 ± 1	3 ± 2	1.8

^*a*^Multiplication of *in vitro* IC_50_ against these integrins.

To validate our *in vitro* data and determine the efficacy of the peptides against the pro-angiogenic integrins in a complex cellular milieu, we subjected the peptides to a competitive cell-adhesion assay (Table 1) utilizing MDA-MB-231 human breast cancer cells expressing αvβ5 integrins, and U-87 MG human glioblastoma cells expressing αvβ3, αvβ5 and α5β1 integrins.^27^ Despite the moderate affinity of **6** towards the pro-angiogenic integrins *in vitro*, it failed to show any efficacy at all in the cell-based assay, which could be attributed to the off-target binding associated with the conformational flexibility in **6**. On the other hand, we observed a remarkable correlation between the *in vitro* affinity of the thioamidated peptides against the purified integrins and their efficacy *in cellulo* (αvβ5 with MDA-MB-231, and αvβ3 and αvβ5 with U-87 MG). This suggests the high specificity and selectivity of the thioamidated analogs towards the pro-angiogenic integrins in the presence of complex cellular components, which can otherwise sequester the peptides, reducing their efficacy.

We were quite excited to note that as detected in the solid phase assay, **7** strongly competed with vitronectin (Vn) in inhibiting the adhesion of MDA-MB-231 cells, suggesting its strong affinity towards αvβ5. A relatively lower potency of **7** was observed when inhibiting U-87 MG cell-adhesion to Vn (Vn binds to αvβ3 and αvβ5),23*a* which is in accord with the lower affinity of **7** towards αvβ3. **11** was the other member of the thio-scan library that showed better affinity than **6** towards the pro-angiogenic integrins *in vitro*, which was also reflected by its efficacy in the cell-adhesion assay.

The lowest affinity towards the pro-angiogenic integrin αvβ3 was obtained for **9** with thionation at Arg, which led to a complete reshuffling of the residues about which both the turns in **9** were centered (Fig. 2B), resulting in a total loss of its efficacy. However, since **9** displayed a βII′- and γ-type turn, we hypothesized that a subsequent spatial screening by altering the spatial position of the pharmacophore on the cyclic peptide backbone might improve the affinity and efficacy of the resulting peptides.

The spatial screening of **9** resulted in **9a–9d** (Fig. 2C), of which **9c** and **9d** did not show any appreciable change in affinity and efficacy against the pro-angiogenic integrins. However, **9a** displayed a remarkable improvement in affinity, and the most exciting observation was the dramatically enhanced (∼10^5^ fold) affinity and efficacy of **9b** in comparison to **9**, by simple alteration in the spatial positioning of the pharmacophores on the thioamide backbone. To emphasize the effect of thio substitution, we synthesized compound **12** (a non-thio analog of **9b**) (Fig. 2C), which showed moderate affinity only towards αvβ3 and α5β1 integrins with no efficacy in the cell-adhesion assay. Additionally, we noted that **12** also displayed two conformers (1 : 2) on the NMR time scale, as observed in **6**, whereas **9b** (along with **9a**, **9c** and **9d**) showed a single conformation, further reinforcing the rigidifying role of thioamides in peptide macrocycles.

Interestingly, the backbone conformations of **9** and the spatial screen analogs (**9a–9c**) were comparable, except for **9d**, which did not undergo the conformational switch (Fig. 2D). This is due to the absence of C^β^ in glycine resulting in its ability to adopt a left-handed α-helical conformation,^28^ allowing the conformation of **9d**. This result, along with the high temperature coefficient value of the thioamide in **9d**, re-emphasizes the fact that the van der Waals interactions between the C=S group and the surrounding atoms are critical in dictating the conformation of thioamidated macrocycles.

**Fig. 3:**
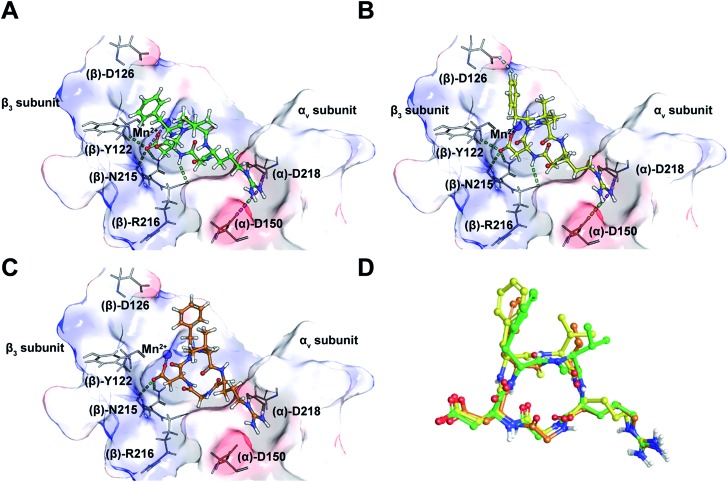
(A) The interaction of cilengitide (shown as a green ball and stick model) bound to the αvβ3 integrin in the PDB entry 1L5G. (B) The interaction of docked **9b** (shown as a yellow ball and stick model) with the αvβ3 integrin. (C) The interaction of docked **7** (shown as an orange ball and stick model) with the αvβ3 integrin. (D) An overlay of the docked poses of **9b** and **7** onto that of **Cilen** co-crystallized with the αvβ3 integrin in ; 1L5G. The green, magenta and cyan dashed lines represent hydrogen bonds, salt bridges and aromatic hydrogen bonds, respectively. The binding site of the protein is shown as a surface representation and the carbon atoms of the protein residues are shown in grey. Oxygen, nitrogen, hydrogen and sulphur atoms are shown in red, blue, white and yellow, respectively. Please see the ESI† for a detailed description of the interactions between the various ligands and the αvβ3 integrin.

Taking into consideration the *in vitro* and *in cellulo* activity of the synthesized peptides (Table 1), we then performed docking simulations to understand the structure activity relationships of the thioamidated peptides with respect to the αvβ3 integrin, for which the crystal structure in complex with **Cilen** is already known (Fig. 3A).^29^ The most potent thioamidated peptide **9b** and **Cilen** display a similar bound pose, the similarity being closest at the –RGD– motif (Fig. 3D) (ESI†). The important electrostatic interactions mediated by Arg and Asp of the –RGD– motif and the hydrophobic contacts mediated by Gly and the aromatic ring of Phe are also maintained in **9b**.^29^ Additionally, **9b** shows an aromatic hydrogen bond (C–H···O interaction) between H^ε^ of Phe and one of the carboxylate oxygens of (β)-Asp126 (Fig. 3B). The C–H···O interactions are very weak but play an important role in protein–ligand binding events.^30^ This extra C–H···O interaction, together with the improved van der Waals interactions (mediated by a S atom as discussed later), possibly contributes to the slightly improved inhibitory potency of **9b**.

The docked poses of all the other thioamidated peptides do not show such a C–H···O interaction, except for **11**. However, the lower potency of **11** stems from the loss of some crucial interactions with the receptor mediated by the –RGD– motif (ESI†). Likewise, all other analogs except **9b** show the loss of one or multiple electrostatic interactions with the receptor. The substitution of O with S provides a greater contact surface area between the peptide and the receptor, which leads to improved van der Waals interactions mediated by the C=S group compared to the C=O group.^10^ Although we did not notice any direct hydrogen bonds between the protein and S atom,^31^ we cannot fully negate the possibility of any water mediated hydrogen bonding between the S atom and the receptor.^32^

Interestingly, **9b** and **7**, which differ only in the chirality about the phenylalanine, show comparable affinity for αvβ5 and α5β1, but >10^3^ fold difference in αvβ3 affinity. Docking of **7** revealed that this difference is due to the smaller distance between Arg and Asp C^β^ compared to **Cilen** and **9b**, leading to the loss of end-on hydrogen bonding between Arg and (α)-Asp150 (Fig. 3C) and the side chain mediated interaction of Asp with the backbone of (β)-Tyr122. Additionally, the backbone mediated interaction of Asp and (β)-Arg216 is also lost due to a flip in the Gly–Asp amide bond (Fig. 3D). This result demonstrates how an inversion of chirality drastically affects the overall efficacy of a macrocyclic peptide.^26^ Lastly, our docking results were nicely validated by **9**, which did not yield any meaningful binding mode (ESI†). The complete loss in its affinity could be rationalized by the absence of biologically relevant interactions mediated by the Asp and Arg side chains with αvβ3. Thus, our *in silico* analysis corroborates the *in vitro* and *in cellulo* results.

### Thionated macrocyclic peptides show minimal non-specific cytotoxicity and enhanced serum half-life

**Fig. 4:**
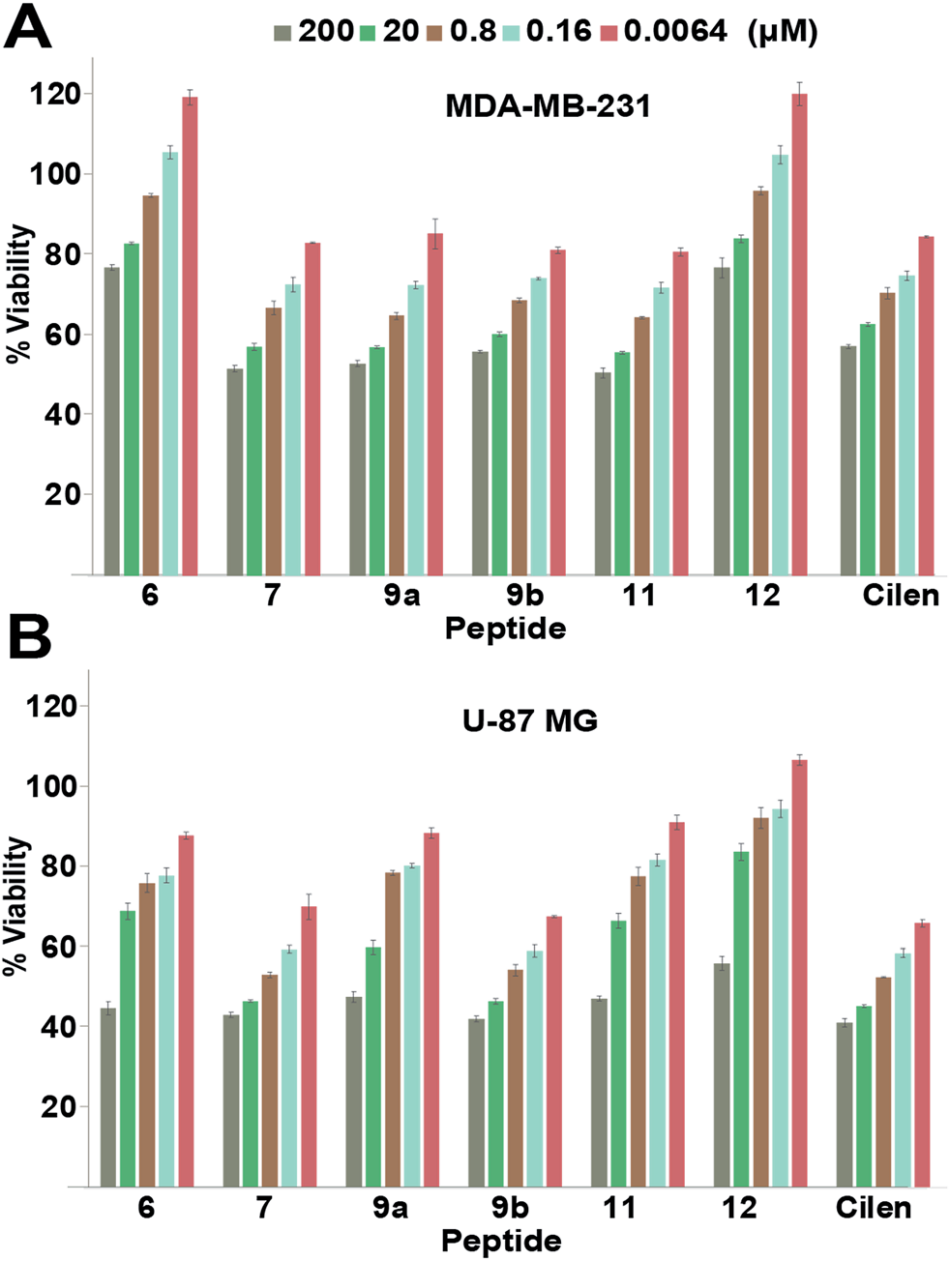
Proliferation of (A) breast cancer cells and (B) glioblastoma cells at 48 h; *n* = 4 ± SEM. Note the slow dose-dependent decrease in proliferation and minimal cytotoxicity even with a 2 × 10^4^ fold increase in initial concentration. Please refer to the ESI† for the entire dose-dependent study.

Since integrin antagonists are known to inhibit the proliferation of cancer cells,^33^ we assessed the effect of a subset of these analogs on the proliferation of MDA-MB-231 and U-87 MG cells and simultaneously determined whether the incorporation of thioamides in peptide macrocycles leads to unspecific cytotoxicity, which is of paramount importance in lead design (Fig. 4). Although in both the cell types we observed a moderate dose-dependent decrease in cell viability at 48 h, a very high concentration of the thio-analogs and **Cilen** was required to achieve the half maximal inhibitory concentration (IC_50_). This clearly suggested that incorporation of thioamides into macrocyclic peptides does not result in unspecific cytotoxicity even after 48 h, which is an important concern for the use of thioamides in lead design. We also noted that glioblastoma cells were more sensitive towards these antagonists (**Cilen**, **9b** and **7**) than breast cancer cells, presumably due to the expression of all the pro-angiogenic integrins, despite the known resistance of U-87 MG cells against detachment-induced apoptosis.^27^ Furthermore, as expected the least efficacious flexible cyclic peptides **6** and **12** displayed minimal detachment-induced apoptosis in breast cancer cells compared to the potent thioanalogs.

**Fig. 5:**
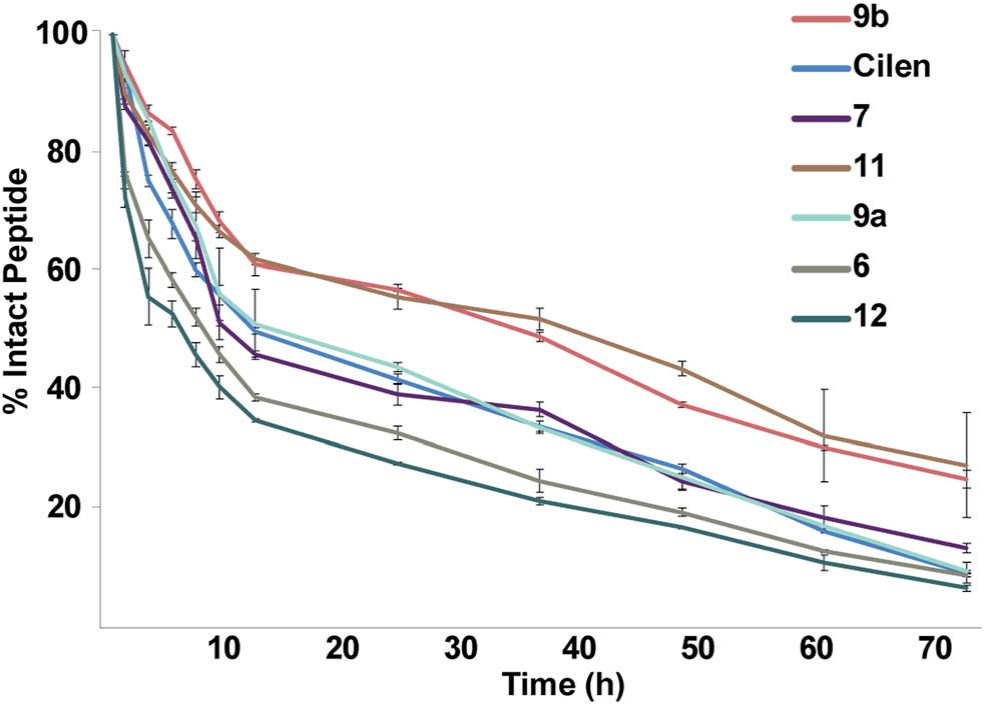
The *ex vivo* metabolic stability of the macrocyclic peptides in freshly isolated human serum for 72 h; *n* = 6 ± SEM. Note the rapid degradation of the unmodified cyclic peptides **6** and **12**. Even at 72 h, 1/4 of the thioamidated peptides **9b** and **11** are intact.

One major drawback to translating peptides as drugs is their inherent metabolic instability due to the action of endo- and exoproteases.4*b* Thus, we determined the stability of the unmodified peptide macrocycles, **Cilen** (*N*-methylated peptide) and the most potent thioanalogs in human serum *ex vivo* for 72 h (Fig. 5). As expected, **6** showed slightly better metabolic stability (*t*_1/2_: 9 h) than **12** (*t*_1/2_: 7 h) due to the presence of d-Phe. It is interesting to note the significant improvement in metabolic stability of all the thioamidated analogs throughout the course of the experiment as observed previously in linear thionated peptides.12a,b,e,f Since these analogs have thionation at different amino acids, we speculate that thioamidation has the potential to significantly improve the metabolic stability of macrocyclic peptides irrespective of the site and amino acid being substituted. It was also quite striking to note that **11** and **9b** showed a significantly improved serum half-life (*t*_1/2_: 36 h each) compared to that of the clinical candidate **Cilen** (*t*_1/2_: 12 h). These data collectively suggest that thioamidation could have a stronger influence in improving the metabolic stability of macrocyclic peptides than d-amino acid incorporation.

## Conclusions

In summary, we have demonstrated that macrocyclic peptides exhibit conformational flexibility in both apolar (model alanine peptides) and polar environments (integrin antagonists), which tends to reduce their bioactivity.3a,b,4c We observed that C=O to C=S substitution in peptide macrocycles dramatically reduces the conformational flexibility while minimally perturbing the global conformation as observed in the model alanine, thio-scan and spatial screen integrin antagonists. Thus, thioamide incorporation leads to predictable changes in the conformation of the peptide backbone, unlike other amide bond modifications. Thioamide incorporation fine tunes the 3D-pharmacophore orientation, which qualifies thioamides as important bioisosteric mimics of peptide bonds.^34^ The remarkable correlation between the *in vitro* and *in cellulo* data against the pro-angiogenic integrins demonstrates the specificity of these thioamidated macrocyclic peptides against their endogenous target, which can be extended to other bioactive peptides as well. Thioamidated macrocyclic peptides are non-cytotoxic and, presumably due to their marked conformational rigidity, display enhanced stability against degradation by proteases,^35^ leading to their increased therapeutic efficacy. Due to the adoption of well-defined structures (turns) caused by increased conformational rigidity, thioamides might have the potential to increase the membrane permeability of cyclic peptides.^36^

It is curious to note that few thioamide containing peptide natural products, such as thioviridamide, thioholgamides, and their related analogs,^37^ which are ribosomally synthesized and post-translationally modified (RiPP), or are of non-ribosomal origin (closthioamide),^38^ show potent antibacterial and antineoplastic activity compared to their all-oxo-amide variant.38*a* Thus, the potent biological activity in these compounds possibly arises from a combination of conformational restriction, increased metabolic stability and enhanced cellular permeability by thio substitution. Furthermore, as observed in **10**, the conformational restriction imparted by the thioamidation of glycine is also predicted to stabilize the local conformation in the vicinity of the active site in methyl-coenzyme M reductase, leading to the increased half-life of the protein.^39^ Therefore, we strongly believe that this single atom substitution, by successfully overcoming the two major drawbacks of peptides *i.e.* conformational flexibility and metabolic instability, will accelerate the discovery of peptide-based drugs and antibacterials.^40^ All the efficacious thioamidated macrocyclic peptides reported here have the potential to block angiogenesis *in vivo* and are currently being investigated.

## Conflicts of interest

There are no conflicts of interest to declare.

## Supplementary Material

Supplementary information
