# Supplementary material for: Increasing the bioactive space of peptide macrocycles by thioamide substitution
Source: Chem Sci. 2018 Jan 22;9(9):2443–51. doi: 10.1039/c7sc04671e (PMC5909342; doi:10.1039/c7sc04671e)
Supplement: Supplementary file 1 [file SC-009-C7SC04671E-s001.pdf]

Electronic Supplementary Material (ESI) for Chemical Science.
This journal is © The Royal Society of Chemistry 2018

Supporting Information

Increasing the Bioactive Space of Peptide Macrocycles by Thioamide Substitution

Hitesh Verma, Bhavesh Khatri, Sohini Chakraborti and Jayanta Chatterjee

Molecular Biophysics Unit, Indian Institute of Science, Bangalore 560012, India

Table of Contents:

1. Materials and Methods …….……………..………………………………… S2
    a. Peptide Synthesis S3
    b. NMR Acqusition/Structure Calculation ..……..………………… S5
    c. Competitive solid phase integrin binding assay …...…...…….. S6
    d. Cell adhesion inhibition assay ....…………………………...... S7
    e. MTT Assay ..………..…………………………........……... S7
    f. Serum Stability Assay ....………………………………….. S7
    g. *In silico* Experiment S7
        i Table S1 List of peptides used for docking S8
        ii Figure S1 Flowchart for docking study S8
2. Table S2 and Figure S2-17 characterization of **P to 12** ……..…………………….. S9
3. Table S3 : Chemical Shifts of **1-5** …………..……………………...............… S26
4. Table S4: ($_{3}J^{HNH\alpha}$) and H^N temperature coefficient of **1-5** …..............…..… S26
5. Figure S18-23 1D HSQC Overlay of **P-5** S26
6. Table S5 : Chemical shifts of major and minor conformer of **6** and **12** …..… S28
7. Figure S24-25 ^{1}H TOCSY of **6** and **12** with strip plot S29
8. Table S6 : Chemical shifts of **7-11**…………..……………………..............… S31
9. Figure S26-35 ^{1}H overlay of **6-12** acquired at different temperature S31
10. Figure S36-43 : Stereoview of peptides **7**, **9**, **9a-d, 10** and **11**………..…...... S34
11. Table S7-20 : Violation list of peptides ..……………………………......... S36
12. Table S21 : Φ-Ψ angles of peptide **P-5** over the trajectory………................ S47
13. Figure S44-45: MTT Assay of MDA-MB-231 and U-87 MG……........... S47
14. Figure S46-52: HPLC chromatogram ovelay for serum stability S48
15. Results and discussion of *in silico* experiment S52
16. Table S22 : Summarised analysis of *in silico* experiment S58
17. Figure S59-62 Mechanistic insight into degradation of **8** S60
18. References………………………………………………….................. S62

Materials and Methods:

All the Fmoc and orthogonally protected amino acids, 1-hydroxybenzotriazole (HOBt), O-(6-chlorobenzotriazol-1-yl)-N,N,N’,N’-tetramethyluronium hexafluorophosphate (HCTU), (1[Bis(dimethylamino)methylene]-1H-1,2,3-triazolo[4,5-b] pyridinium 3-oxid hexafluorophosphate) (HATU), 1-hydroxy-7-azabenzotriazole (HOAt), 2-Chlorotrityl chloride polystyrene (2Cl-TCP) were purchased from GL Biochem, Shanghai, China. *N,N’*-Diisopropylcarbodiimide (DIC), *N,N*-diisopropylethylamine (DIPEA), Trifluoroacetic acid (TFA), Trifluoroethanol (TFE), Triisopropylsilane (TIPS), Triphenylphosphine, anhyd. tetrahydrofuran (THF), anhyd. methanol, Diisopropyl azodicarboxylate (DIAD), 1,8-Diazabicyclo[5.4.0]undec-7-ene (DBU), 2-Mercaptoethanol, Glacial acetic acid, *N,N'*-Dicyclohexylcarbodiimide (DCC), N-methyl-2-pyrrolidone (NMP) , Thionyl Chloride, Calcium hydride and piperidine were purchased from Sigma-Aldrich. All the above reagents were used as commercially supplied. Solvents for RP-HPLC were purchased as HPLC grade and used without further purification. Dichloromethane was dried with Calcium hydride. All the other solvents were used as commercially supplied.

All the reactions were performed in oven-dried glass apparatus. Reactions on solid support were carried out in plastic syringes (10 ml) fitted with a frit column plate.

High-resolution mass spectra were recorded on a Bruker Daltonics ESI Q TOF- (Maxix Impact) with Nano LC (Proxeon easy nLC) mass spectrometer. ESI mass spectra were recorded in positive ion mode on a HCTultra ETD II ion trap spectrometer (PTM Discovery System, Bruker Daltonics, Germany). MALDI mass spectra were recorded on UltrafleXtreme TOF/TOF (Bruker Daltonics, Germany) and the data were processed and analysed using the Flex Analysis 3.1 software.

Nuclear magnetic resonance (NMR) spectra were recorded on a 700 MHz and 500 MHz Bruker Avance spectrometer (Bruker, Karlsruhe, Germany), and also on 600 MHz Agilent NMR spectrometer at 298K. All chemical shifts are reported in parts per million (ppm) from tetramethylsilane (TMS) ($\delta = 0$) and were measured relative to the solvent $(CD_3)_2SO$: δ 2.49 ppm for ^{1}H NMR, δ 39.9 for ^{13}C NMR).

Analytical RP-HPLC was performed on a Shimadzu UFLC system equipped with Prominence Diode Array (PDA) UV Detector at 210 and 254 nm using an analytical column (Phenomenex C18, 250 mm x 4.6 mm I.D., 5 µm) at a flow rate of 1 mL min^{-1}. Purifications were performed using a semi-preparative column (Phenomenex C18, 250 mm x 10 mm I.D., 5 µm) at a flow rate of 4 mL min^{-1}.

Human integrins αvβ3, αvβ5, α5β1, αIIbβ3, extracelluar matrix proteins: vitronectin, fibronectin, fibrinogen, mouse anti-human CD49e and CD41b were purchased from Millipore. Anti-mouse IgG-peroxidase, 3,3,5,5’-tetramethylethylenediamine (TMB), 3M H_2SO_4 from Sigma. Neutravidin-horseredish peroxidase (HRP) and sulpho-NHS-LCLC-biotin from Thermo scientific.

All cell culture materials like media, FBS, antibiotic etc. were purchased from Himedia Labs.

Peptide Synthesis

Peptides **P** to **12** were synthesized on TCP resin (1.3 mmolg^{-1}), using standard Fmoc based chemistry. The C-terminal amino acid residues (1.25 equiv) were loaded on to the resin with 2.5 equiv DIPEA in anhydrous DCM at room temperature. After loading the first amino acid, the remaining unreacted trityl chloride groups bound to the solid support were capped using methanol (200 µl/100 mg resin) for 15 min. Next, the resin was thoroughly washed with DCM (3 times), 1:1 DCM-methanol (3 times) and methanol (3 times) and finally dried under vaccum. The loading capacity was estimated from the dry weight of the resin, which ranged from 0.6-0.8 mmolg^{-1}. The elongation of the rest of the peptide was performed on 250 mg (0.14-0.25 mmol) scale with DIC/HOBt as the coupling agents (2.5 equiv). Fmoc deprotections were carried out with 20% piperidine (5 min x 1, 15 min x 1) in DMF. 10% piperidine (30 sec x 2) in DMF was used for residue coupled after the incorporation of thionated amino acid.

General method for the synthesis of the aminoanilide precursors[1]:

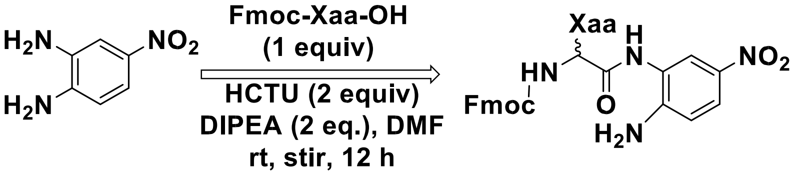

Following the reported protocol, a mixture of diisopropylethylamine (2 equiv) and diaminonitrobenzene (1 equiv) was added to a DMF solution of Fmoc-Xaa-OH (1 equiv) and HCTU (2 equiv) at room temperature. After 12 h of stirring, this reaction mixture was poured into a saturated KCl solution and extracted with ethyl acetate, washed thoroughly with brine, dried and concentrated to a sticky mass. The yellow solid obtained after drying the mass under high vacuum was used for the next step of synthesis without further purification and characterisation. (All the aminoanilide precursors except for arginine aminoanilide, were used directly for the following step) General method for the synthesis of the thioamide precursor:

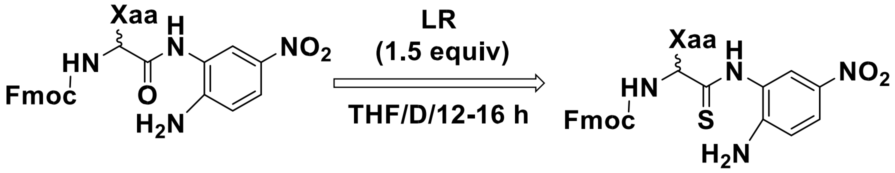

To a solution of aminoanilide precursor in dry tetrahydrofuran (THF), 0.75 equiv of Lawesson's reagent was added to reflux the mixture at 70 °C for 2h. An additional 0.75 equiv of the same was added to push the reaction to completion by overnight reflux. The progress of the reaction was monitored by thin layer chromatography (TLC). Upon completion, the solvent was evaporated and the crude mixture was purified by column chromatography on silica gel (10-100 mesh size) using 10-80 % gradient of hexane/ethyl acetate to yield pure thioamide precursor. (To obtain the glycine thioamide precursor 2e, the thionation was carried out at room temperature instead of refluxing condition.) General method for the synthesis of the benzotriazolide Precursors:

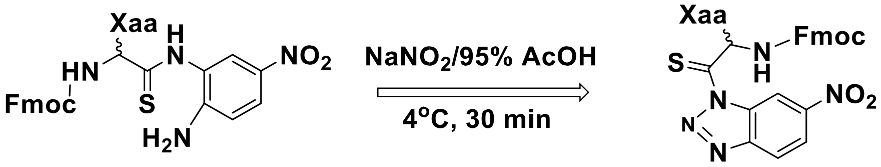

To a solution of thioamide precursor in glacial acetic acid containing 5% water, 1.1 equiv of odium nitrite was added in portion at 4 °C. The reaction mixture was stirred at 4 °C for 30 min. The progress of the reaction mixture was monitored by thin layer chromatography (TLC). Upon completion, chilled water was poured onto the reaction mixture. The light orange precipitate hence formed was filtered, dried and dissolved in dichloromethane. This solution was immediately utilized for the incorporation of the thioamide moiety onto solid support in the next step without further purification.

General method for the incorporation of the thioamide on solid support:

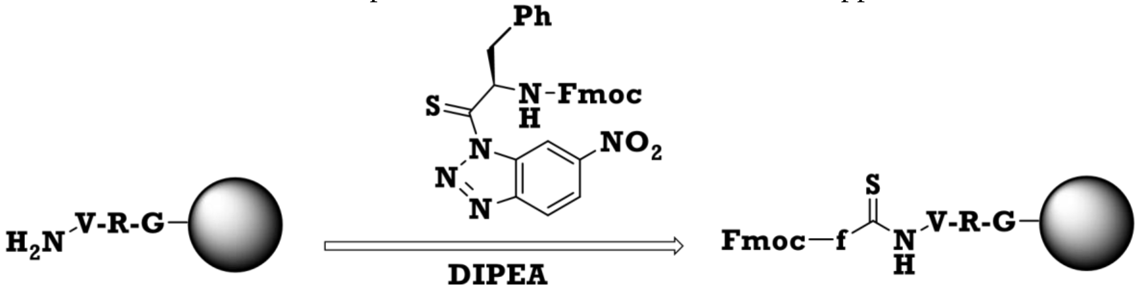

The Fmoc dedprotected peptide was swollen in dichloromethane in a fritted syringe prior to the coupling. Half of the solution of benzotriazolide precursor (around 1.5 equiv) in dichloromethane was taken into the syringe along with 60 µl (0.75 equiv) of diisopropylethylamine. This mixture was stirred for 45 min under protection from light. The fluid inside the syringe was squeezed out and the resin was thoroughly washed with dichloromethane to remove any residual reagents. Similarly the rest portion of benzotriazolide precursor was taken into the syringe along with the base to stir for 45 min under protection from light. Finally the resin was washed with dichloromethane again to remove any residual reagents. The extension of the peptide was done using DIC/HOBt method in DMF.

N-Methylation:

A modified protocol[2] for Mitsunobu reaction on the solid support was utilized for selective N-methylation of amino acid residue.

Coupling of the amino acid residue following the N-methylated amino acid:

Coupling of Fmoc-Xaa-OH to the free N^{α}-methylamine terminal of the peptides on the resin was carried out using 3 equiv each of HOAt, HATU and Fmoc-Xaa-OH and 6 equiv of DIPEA in DMF at room temperature.[2]

Global Deprotection and Cleavage from the Resin:

Peptides **P** to **12** were cleaved-off from the resin under mild condition with the cleavage cocktail- AcOH:TFE:DCM (3:1:6) for 3 hrs. The syringe was thoroughly washed with dichloromethane and the combined extracts were concentrated in vacuo. The residual acetic acid present in the mixture was co-evaporated with toluene. The colorless sticky

mass hence obtained was then subjected to macrocyclization under high dilution (12 mM) in DMF via DPPA (3 equiv)/$NaHCO_3$ (5 equiv).

Upon completion of the cyclization (monitored by mass spectrometry), the solvent was removed and the concentrated mass was dissolved in 500 µl of DMF and this solution was poured drop wise into 10 ml of chilled water to precipitate the peptide. The peptide was washed by centrifuging thrice (4000 rpm, 20 min) with chilled water. The white mass hence obtained was dried and purified by RP-HPLC using 0.1% formic acid in Water:ACN system. Purified peptide was dried under lyophillizer in high vacuum condition.

The global deprotection of purified protected peptide **6-12** was carried out with 62.5% TFA (TFA:DCM:TIPS:H2O – 62.5:32.5:2.5:2.5) for 30 min. Deprotected peptide was precipitated in chilled ether yielding pure product.

NMR Acquisition

For compounds **P** to **5,** the samples were dissolved in DMSO-*d6*. For compounds **Cilen** and **6** to **12**, the compounds were dissolved in H_2O:D_2O **(**9:1) with 0.1% TMSP used as an internal standard (δ = 0 ppm). Standard Bruker pulse sequences zgesgp for 1-D, mlevesgpph/dipsi2rcesgpph (60 ms mixing time) for TOCSY and roesyesgpph (100 ms mixing time) for ROESY were used with 2048 data points in direct dimension and 512 data points in indirect dimension to acquire the NMR data. Hmbcglpndqf pulse sequence was used for the acquisition of HMBC.

The NMR of all compounds **P** to **12** were obtained using concentration of 1-3 mM.

All NMR data were processed using iNMR (www.inmr.net), and the 2D NMR data were analyzed with SPARKY.[3] The chemical shift tables were generated from TOCSY, HMBC (alanine model peptides) and ^{1}H spectra. The sequential assignments and inter- and intra-residue NOEs were determined through ROESY. The NOEs were then integrated and the integration values were converted to distances using the formula $V=Kd^{-6}$, where V is the integrated peak volume, K is a constant (determined using Hα-Hβ distance on Ala in case of **1-5** and resolved diastereotopic CH_2 groups from Phe or some cases Gly in **7-11**), and d is the distance between the protons.

Determination of temperature coefficient for amide protons were performed by acquiring ^{1}H spectrum at different temperature ranging from 298 K to 323 K at an interval of 5 K in DMSO-*d6* and 278 K to 323 K at an interval of 25 K in 9:1 H_2O:D_2O.

Determinaation of T_1 relaxation time for **P-5** was done on Varian 600 MHz using gCHSQC pulse program. In every experiment different T_1 was taken ranging from 10 ms to 1750 ms. Absolute intensity of Hα pekas were then plotted against the respective T_1 value and using one phase decay analysis ½ T_1 was determined.

Structure Calculation

To calculate the structure of the molecule we have used charmM force field,[4] via the interface of Discovery Studio, for the entire process.

The distance restraints were converted into a charm restraint file using a custom Perl script. The resulting file was then used to define NOE restraints inside the charmM syntax. To the distance, 10% were added or subtracted to define the upper and lower limits respectively. If there were any methyl protons involved in the restraints, an additional 0.4

Å per methyl group (pseudoatom correction) were added to the upper limit to compensate for the errors involved.

The initial structure was obtained by following a simulated annealing protocol. It was then refined by dihedral angle constraints derived from ^{1}H NMR spectra employing Bystrov equation followed by a 10 ns restrained molecular dynamics run. The average over the dynamics run was considered to be the final structure and 10 structures were sampled at equal time intervals to generate the ensemble.

For the structure of **7-11** (including **9a-9d**) explicit solvent box of H_2O was used with periodic boundary conditions. A 2 ns restrained molecular dynamics run was then performed. The average conformation over the dynamics run was considered to be the final structure.

Competitive solid-phase integrin binding assay

The activity of peptide as integrin antagonist was assessed using solid phase integrin binding assay. Following buffers were prepared freshly.

Carbonate buffer (15 mM Na_2CO_3, 35 mM $NaHCO_3$, pH 9.6).

PBS-T-buffer (phosphate-buffered saline/Tween20, 137 mM NaCl, 2.7 mM KCl, 10 mM Na_2HPO_4, 2 mM KH_2PO_4, 0.01% Tween20, pH 7.4; 3 × 200 µL)

TS-B-buffer (Tris-saline/BSA buffer; 150 µL/well; 20 mM Tris-HCl, 150 mM NaCl, 1 mM $CaCl_2$, 1 mM $MgCl_2$, 1 mM $MnCl_2$, pH 7.5, 1% BSA).

Vitronectin-αvβ3 assay : Half-volume, flat bottom, high binding 96-well ELISA plates (Eppendorf) were coated with 50 µl of 0.4 µg/ml of human αvβ3 protein in TS buffer at room temperature. Plates were blocked with 100 µl/well of TSB buffer for 2 h at room temperature.Post blocking plates were washed with 100 µl/well of PBST buffer for three times. Peptides (**Cilen**, **6-12**) were serially diluted in 1:5 ratio and were premixed with 1 µg/ml of biotinylated human vitronectin (suppliers protocol was followed for the biotinylation of vitronectin) and then 75 µl of this solution was incubated in the plates for 2 h at room temperature. After draining the solution five thorough washes with PBST was done. Plates were then incubated with 50 µl/well of 0.25 µg/ml of neutravidin-HRP for 1 h at room temperature. Post-washing plates were developed using 50 µl of TMB substrate. After 15 min reaction was quenched with 3 M H_2SO_4 and reading is taken under varioskan 96-well plate reader at 492 nm.

Vitronectin-αvβ5 assay : Plates were coated overnight with 50 µl of 1 µg/ml of αvβ5 integrin at room temperature. Post-blocking and washing peptides were mixed with biotinylated vitronectin and this mixure was then incubated with coated plates for 2h at room temperature. Similar to αvβ3 assay binding was visualized using neutravidin-HRP and TMB substrate.

Fibronectin-α5β1 assay : Plates were coated overnight with 50 µl /well of 0.5 µg/ml of fibronectin at 4°C in carbonate buffer.After blocking and washing peptide and 1 µg/ml of α5β1 integrin was incubated in coated plates for 1h at room temperature. After washing

1:500 diluted CD49e antibody was incubated with plates for 1 h. Anti-mouse IgG antibody (1:10,000) conjugated with HRP was then used as secondary antibody.
After thorough washing binding was visualized using TMB substrate for 15 min.
Fibrinogen-αIIbβ3 assay : Plates were coated overnight with 50 μl/well of 10 μg/ml of fibrinogen at 4 °C. After blocking and washing peptide along with 2.5 μg/ml of αIIbβ3 integrin was incubated for 1h at room temperature. After washing 1:250 diluted CD41b antibody was incubated for 1h at room temperature. Anti-mouse IgG-peroxidase antibody was used as secondary antibody. Binding was visualized using TMB substrate.

Cell adhesion inhibition assay

96 well plates were coated overnight with 2 μg/ml of vitronectin in TS buffer at room temperature. After washing thoroughly with PBST buffer peptides were serially diluted in the plate. 32,000 cells/well were added in the plate and allowed to adhere at 37 °C. After 2 h solution is dispensed and plates were washed with PBS buffer. Adhered cells were incubated with 0.5% crystal violet for 15 min. Cells were then washed thoroughly with PBS buffer to remove excess of crystal violet. Finally dye was eluted out and dissolved using DMSO and quantification was done at 570 nm.

Cell proliferation assessment of MDA-MB-231 and U-87 MG by MTT assay

5000 cells/well was seeded in 96 well plate in 10% FBS conaining media. Next day media was dispensed and 5% FBS containing media was added. Serially diluted peptide was added in all the wells. After 48 h cells MTT was added for the quantification of cell number. MTT crystals were dissolved in DMSO and absorbance was measured at 590 nm.

Serum Stability assay

Blood was freshly drawn from an adult healthy human and kept still at room temperature for clotting. After 3 h, centrifugation was done at 4000 rpm for 10 min to separate the clot and serum. Serum was then diluted in DMEM media to make 25% v/v solution. 1 mg/ml of peptide was added in 1 ml of serum solution and incubated at 37 °C. Aliquots were isolated at various time point and equal volume of 15% Trichloroacetic acid was used to precipitate the proteins. After centrifugation at 10,000 rpm for 10 min, supernatant was lyophilized. Dried sample was reconstituted in 100 μl of water and injected in HPLC. Peak were idenfied using ESI-MS and quanitification was done by integrating the area under the peak at 200 nm.

***In silico* Experiments**

Material and Methods: 3D co-ordinates of the minimum energy conformer of all the cyclic pentapeptides (as listed below) as obtained from NMR studies were used for the docking simulations after preparing the peptides using the LigPrep module from Schrödinger, LLC (Schrödinger Realease 2017-1). X-ray crystal structure of αvβ3 in complex with Cliengitide (PDB ID: 1L5G) was obtained from Protein Databank[5] and prepared using Protein Preparation Wizard[6] in Maestro Version (11.1.011). The prepared cyclic pentapeptides were then docked into the prepared αvβ3 using Glide.[7] Cilengitide (Cilen) was used as reference compound for the current study. OPLS3[8] force field was used during protein preparation, ligand preparation and docking. The steps followed are summarized in the **Fig S1**

Table S1 : List of peptides used for docking

**Molecule**	**7**	**9**	**9a**	**9b**	**10**	**11**	**Cilen**
**Chemical Structure**	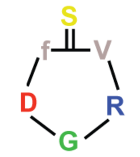	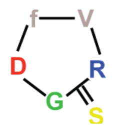	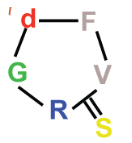	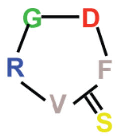	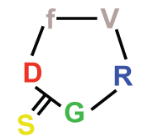	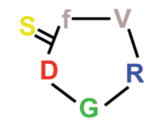	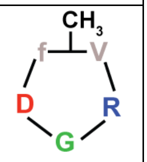

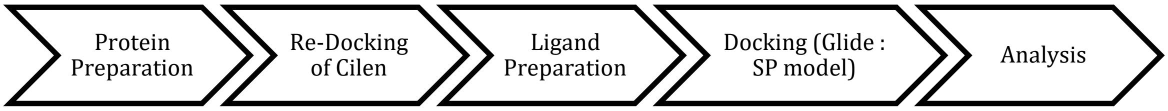

Figure S1 : Flowchart of steps followed for docking simulation study

Cut off values used for interaction analysis.

The following cut-offs for each criteria (which are the default values Maestro) were used for detection of

Hydrogen bonds :

Maximum distance between H atom and acceptor atom (H…A)	2.8Å
Donor minimum angle (D-H…A angle)	120º
Acceptor minimum angle (H…A-B angle)	90º

Salt Bridge :

Maximum distance between two oppositely charged atoms	5Å (default)

Aromatic Hydrogen Bond :

Maximum distance from the H atom to an O acceptor atom	2.8Å
Maximum distance from the H atom to an sp^2 nitrogen atom	2.5Å
Donor minimum angle (D-H…A angle) when O acceptor is involved	90º
Donor minimum angle (D-H…A angle) when sp^2 N atom is involved	108º
Donor maximum angle (D-H…A angle) when sp^2 N atom is involved	130º
Acceptor minimum angle (H…A-B angle)	90º

Contact :

The contact between G of RGD motif in peptide ligands and carbonyl O of (β)-Arg216 were identified using the default 'Good Contact' criteria in Maestro version 11.1.011. The contact criteria is based on the following formula:

$$C=D_{12}/(R_1+R_2),$$

where D_{12} is the distance between two atomic centers 1 and 2, and R_1, R_2 are the van der waal's radii of atomic centers 1 and 2.

For Good contact, the cut-off of distance ratio (C)	0.89<C<1.30

Note : Any hydrogen and acceptor atom in a hydrogen bond is not considered to be in contact by default.

Any distance ratio larger than the good cut off is not considered to be in contact.

Characterization of peptides

Table S2 : HRMS and ESI-MS of peptides **1-12** along with their obtained yield.

				HRMS		ESI-MS		
	Calculated mass			Observed mass		Observed mass		Yield
	$[M+H]^+$	$[M+Na]^+$		$[M+H]^+$	$[M+Na]^+$	$[M+H]^+$	$[M+Na]^+$	in%
**P**	355.1856	378.1753		356.2006	378.1839		378.08	16
**1**	371.1627	394.1525		372.1787	394.1611	371.98	393.98	17
**2**	371.1627	394.1525			394.1616	372.03	393.99	16
**3**	371.1627	394.1525		372.1733	394.1563	371.95	393.99	14
**4**	371.1627	394.1525		372.1786	394.161	371.97	393.99	15
**5**	371.1627	394.1525		372.172	394.1555		393.97	16
**6**	574.2863	597.2761		575.2896	597.2691	575.37	597.32	16
**7**	590.2635	613.2533		591.2671	613.2464	591.33	613.28	15
**9**	590.2635	613.2533		591.2704		591.11		15
**10**	590.2635	613.2533		591.2724	613.2479	591.31	613.26	12
**11**	590.2635	613.2533		591.2742	613.2493	591.28	613.23	14
**12**	574.2863	597.2761		575.2911	597.27	575.34	597.29	15
**9a**	590.2635	613.2533		591.2691	613.2474	591.42	613.33	14
**9b**	590.2635	613.2533		591.2673	613.2481	591.12	613.07	14
**9c**	590.2635	613.2533		591.2621		591.11		14
**9d**	590.2635	613.2533		591.1713	613.1317	591.10	613.04	12

Yield was calculated considering the resin loading and the final cyclic peptide obtained.

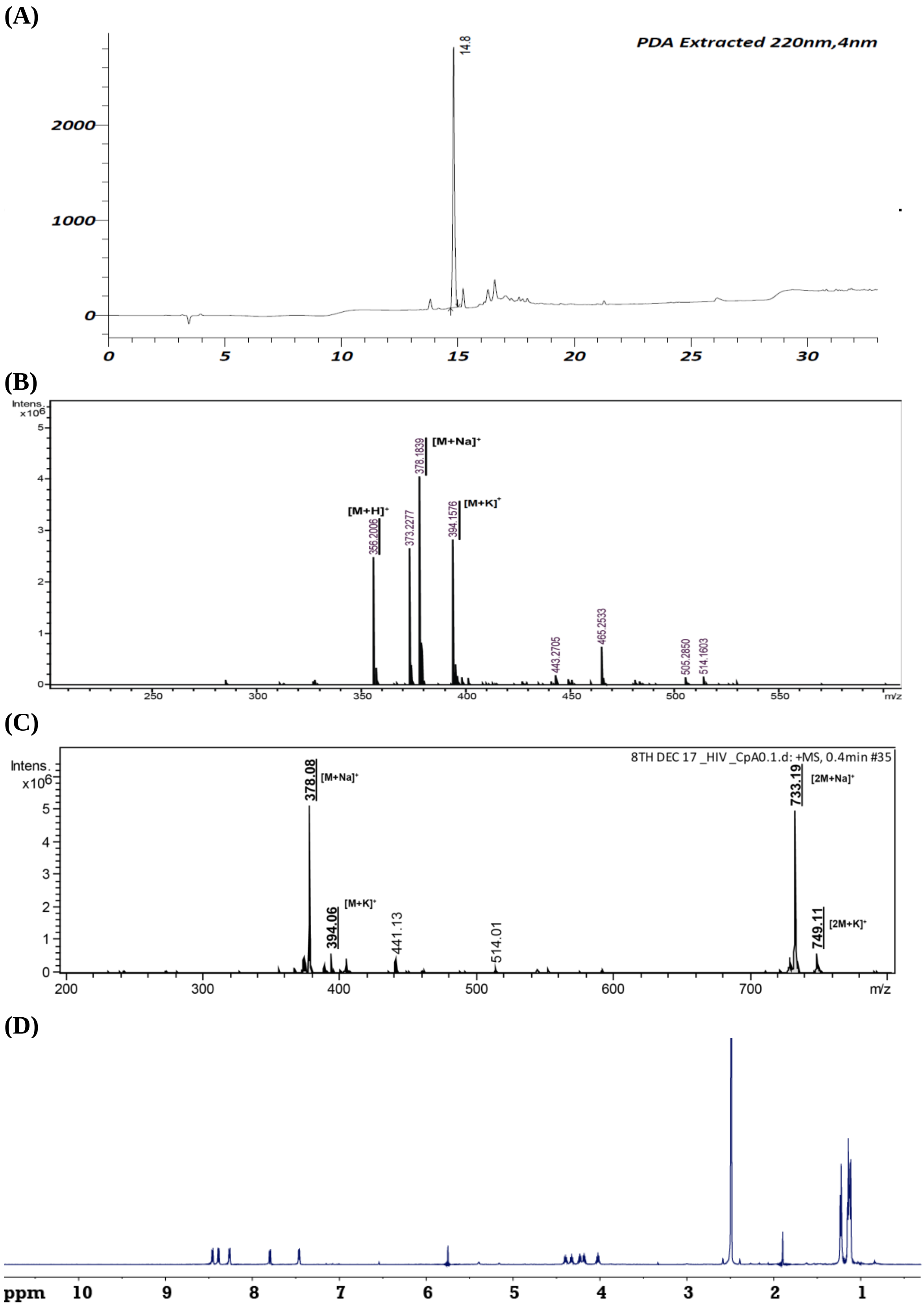

Figure S2 : (A) Analytical HPLC chromatogram of purified peptide **P,** (B) MALDI (HRMS) profile. The higher and lower molecular weight peak is due to unusual fragmentation pattern. (C) ESI-MS profile. (D) One dimensional ^{1}H NMR of pure peptide.

(A)

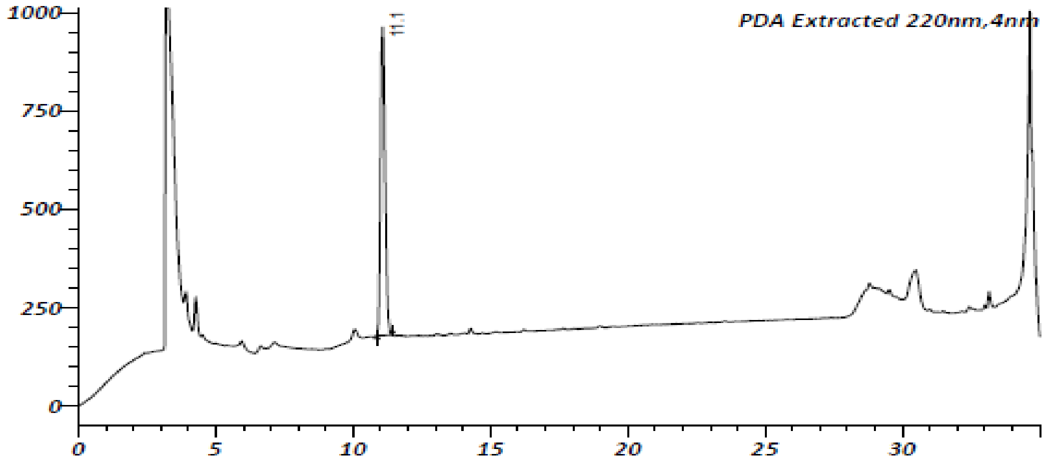

(B)

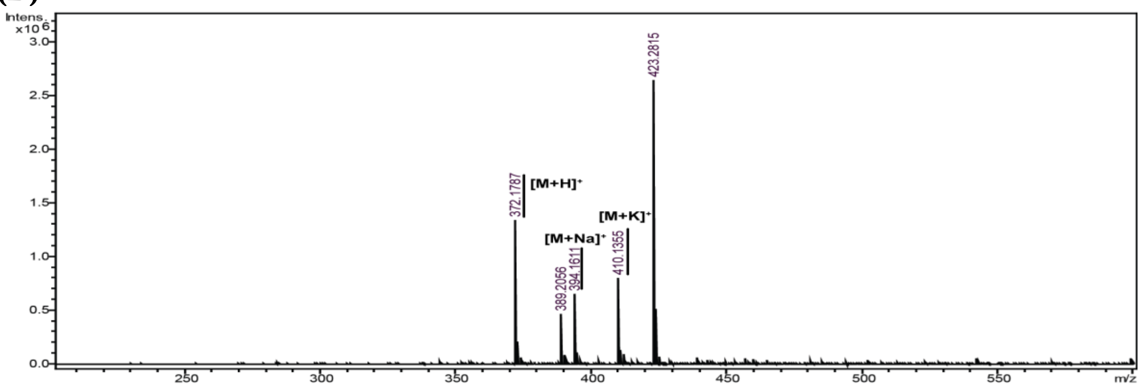

(C)

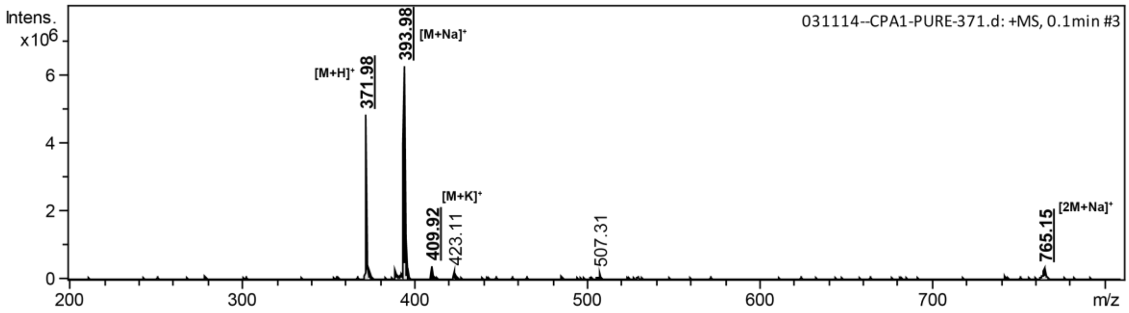

(D)

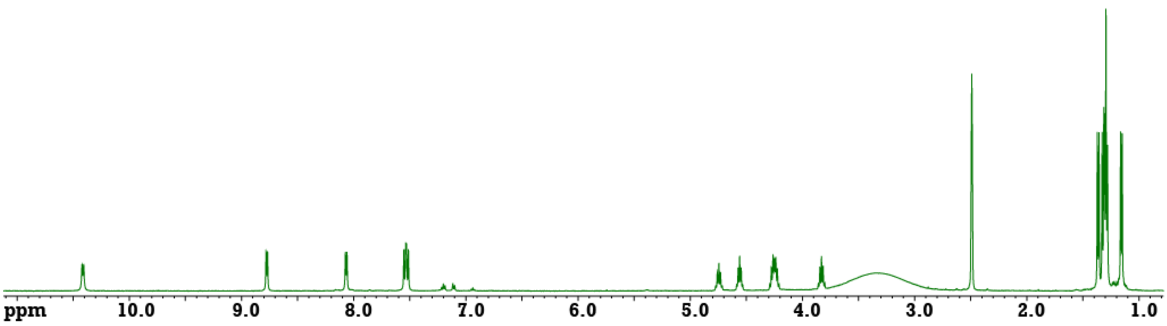

Figure S3 : (A) Analytical HPLC chromatogram of purified peptide **1,** (B) MALDI (HRMS) profile. The higher molecular weight peak is due to unusual fragmentation pattern. (C) ESI-MS profile. (D) One dimensional ^{1}H NMR of pure peptide.

(A)

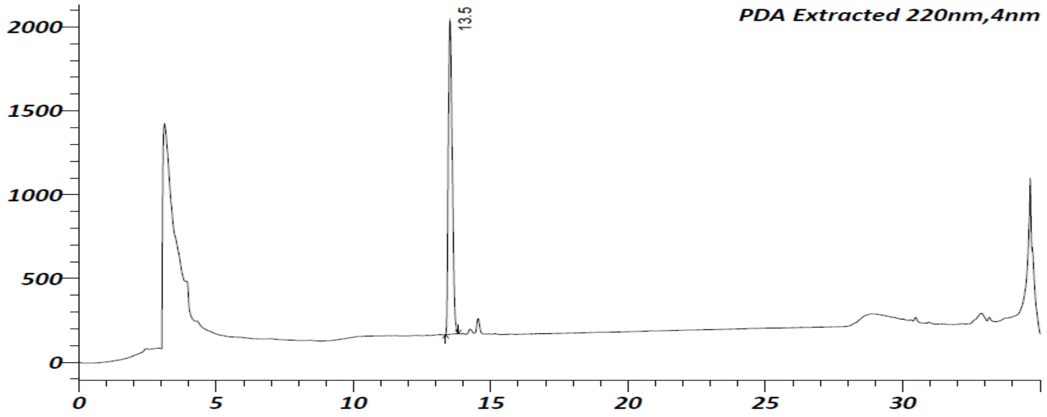

(B)

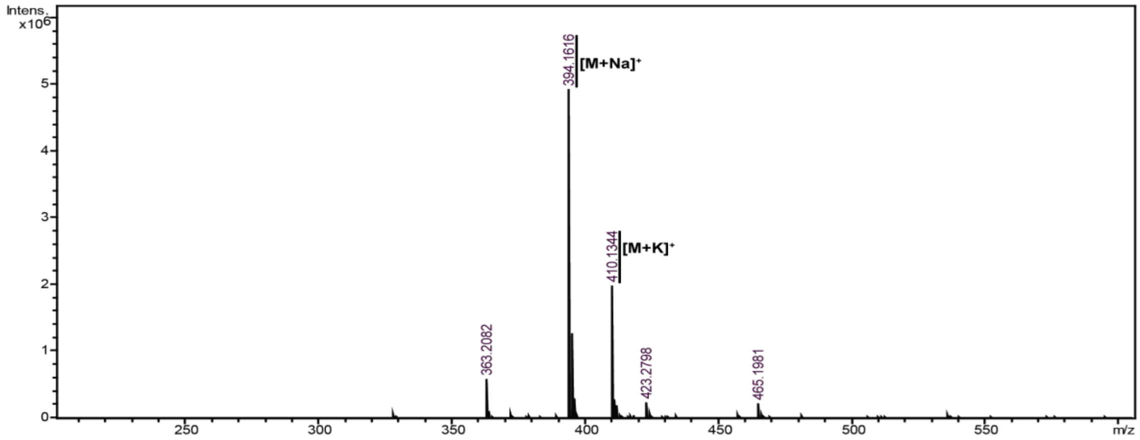

(C)

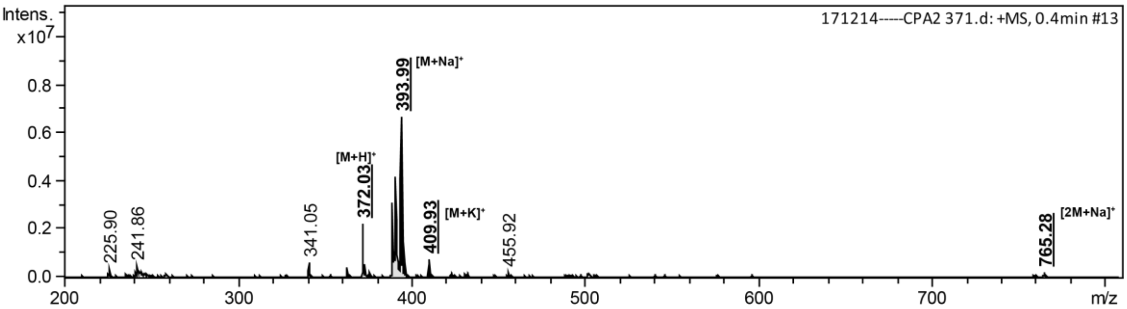

(D)

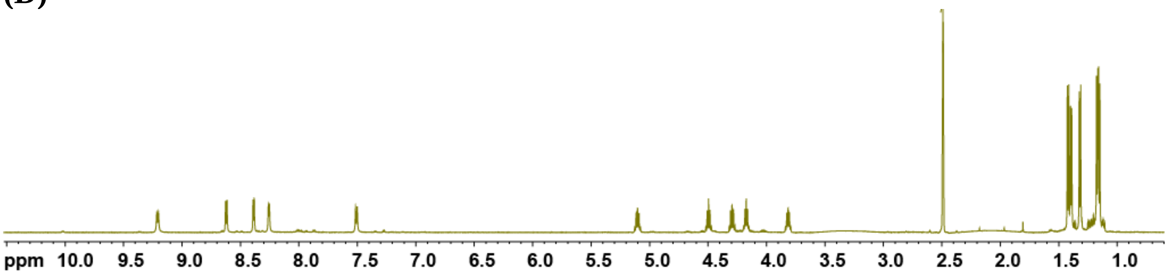

Figure S4 : (A) Analytical HPLC chromatogram of purified peptide **2,** (B) MALDI (HRMS) profile. The higher and lower molecular weight peak is due to unusual fragmentation pattern. (C) ESI-MS profile. (D) One dimensional ^{1}H NMR of pure peptide.

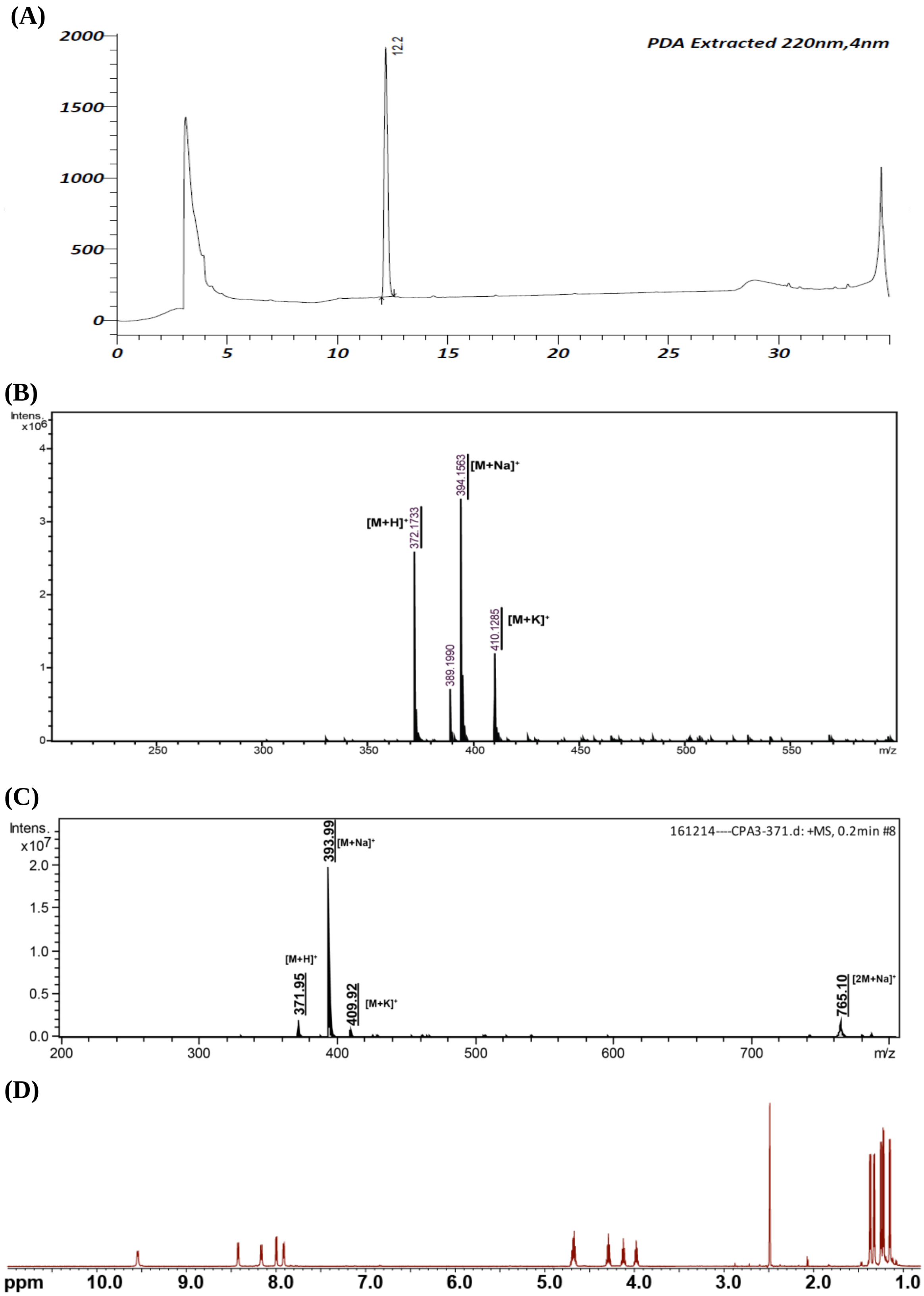

Figure S5 : (A) Analytical HPLC chromatogram of purified peptide **3,** (B) MALDI (HRMS) and (C) ESI-MS profile. (D) One dimensional ^{1}H NMR of pure peptide.

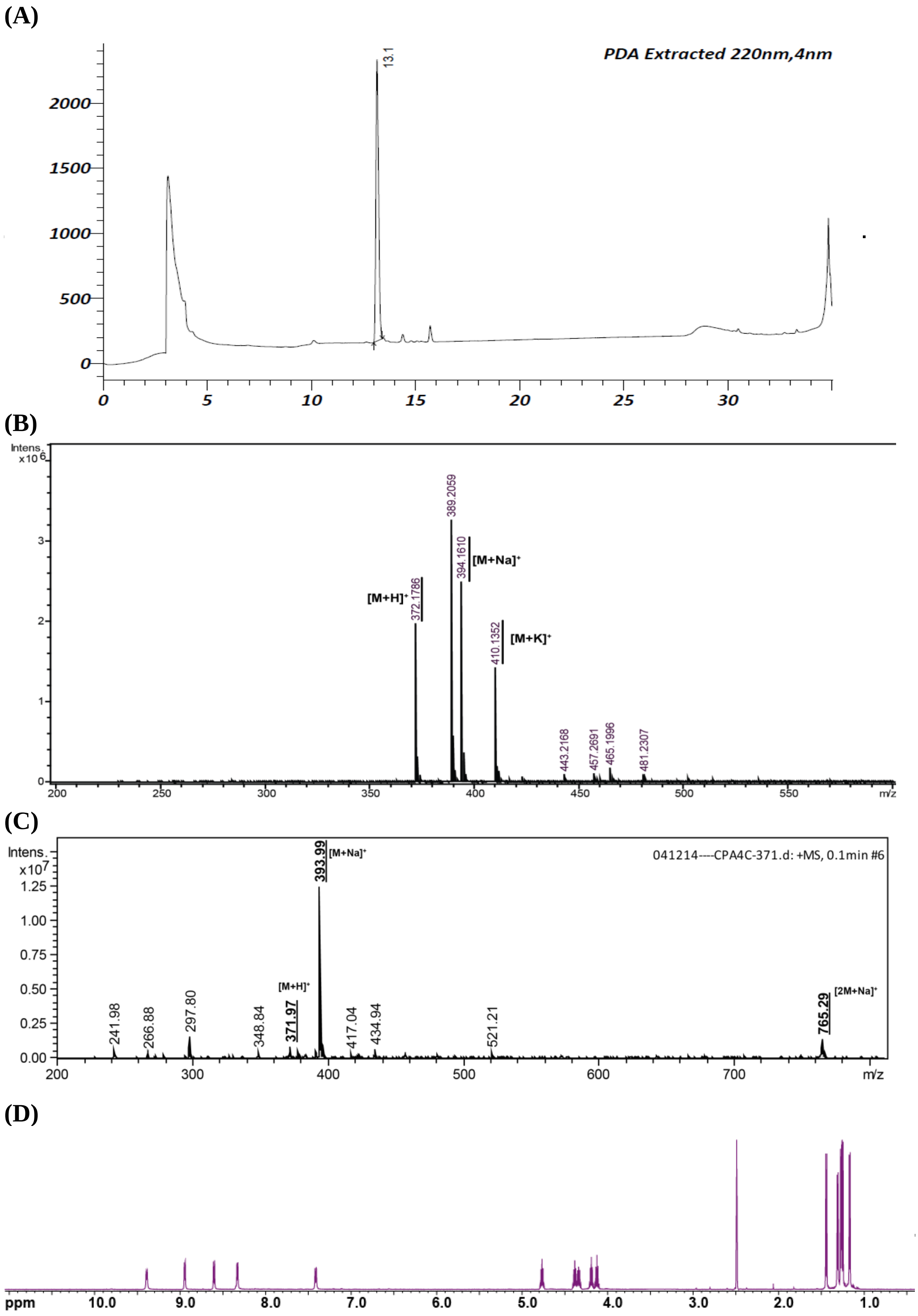

Figure S6 : (A) Analytical HPLC chromatogram of purified peptide **4,** (B) MALDI (HRMS) profile. The higher molecular weight peak is due to unusual fragmentation pattern. (C) ESI-MS profile. (D) One dimensional ^{1}H NMR of pure peptide.

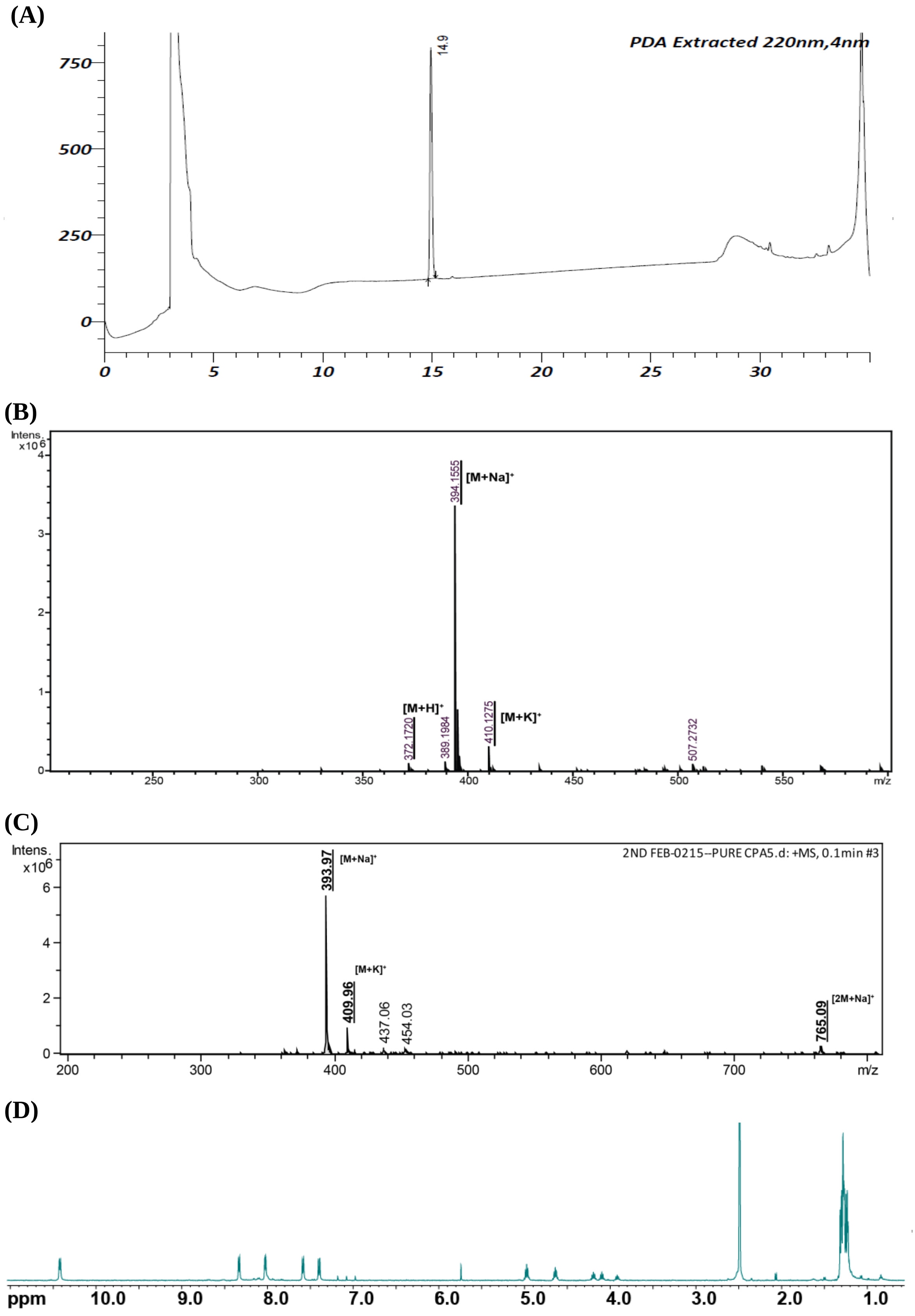

Figure S7 : (A) Analytical HPLC chromatogram of purified peptide **5,** (B) MALDI (HRMS) profile. The higher molecular weight peak is due to unusual fragmentation pattern. (C) ESI-MS profile. (D) One dimensional ^{1}H NMR of pure peptide.

(A)

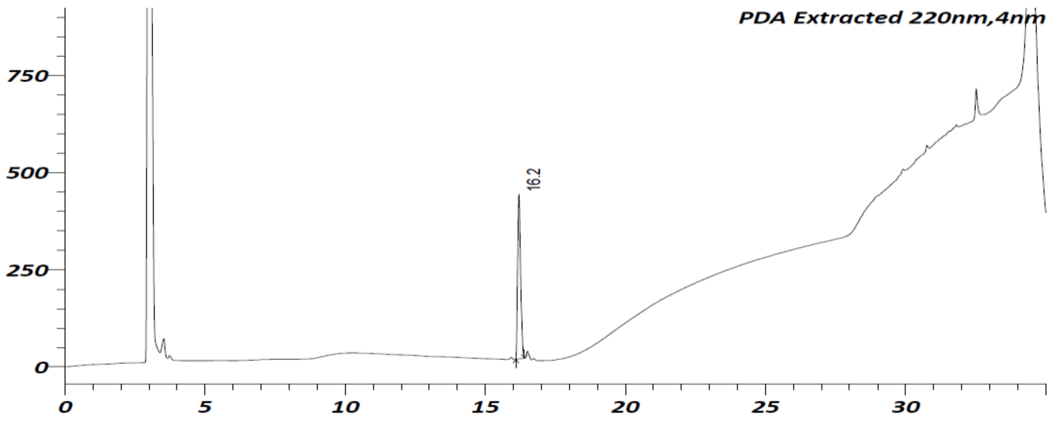

(B)

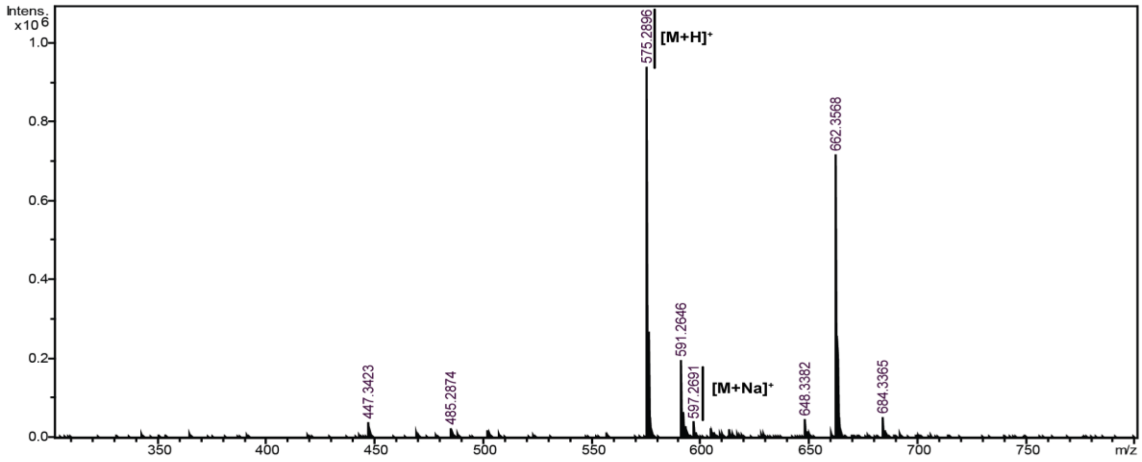

(C)

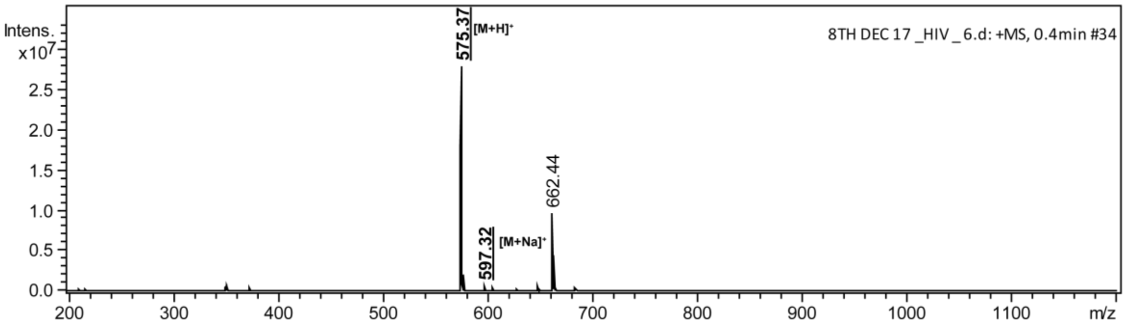

(D)

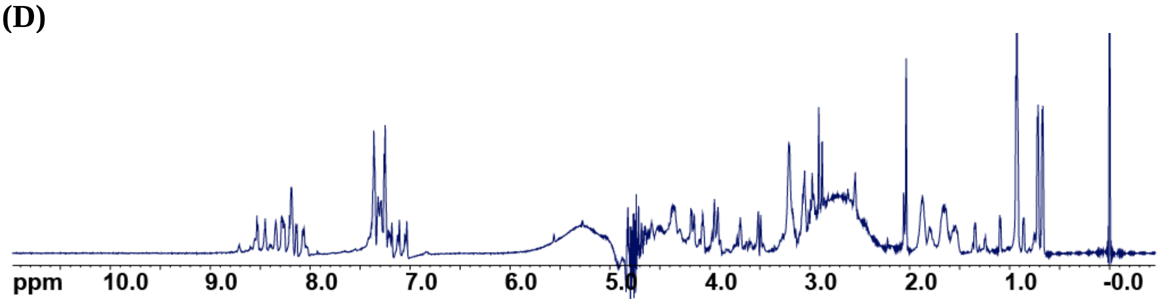

Figure S8 : (A) Analytical HPLC chromatogram of purified peptide **6,** (B) MALDI (HRMS) profile. The higher and lower molecular weight peak is due to unusual fragmentation pattern. (C) ESI-MS profile. (D) One dimensional ^{1}H NMR of pure peptide.

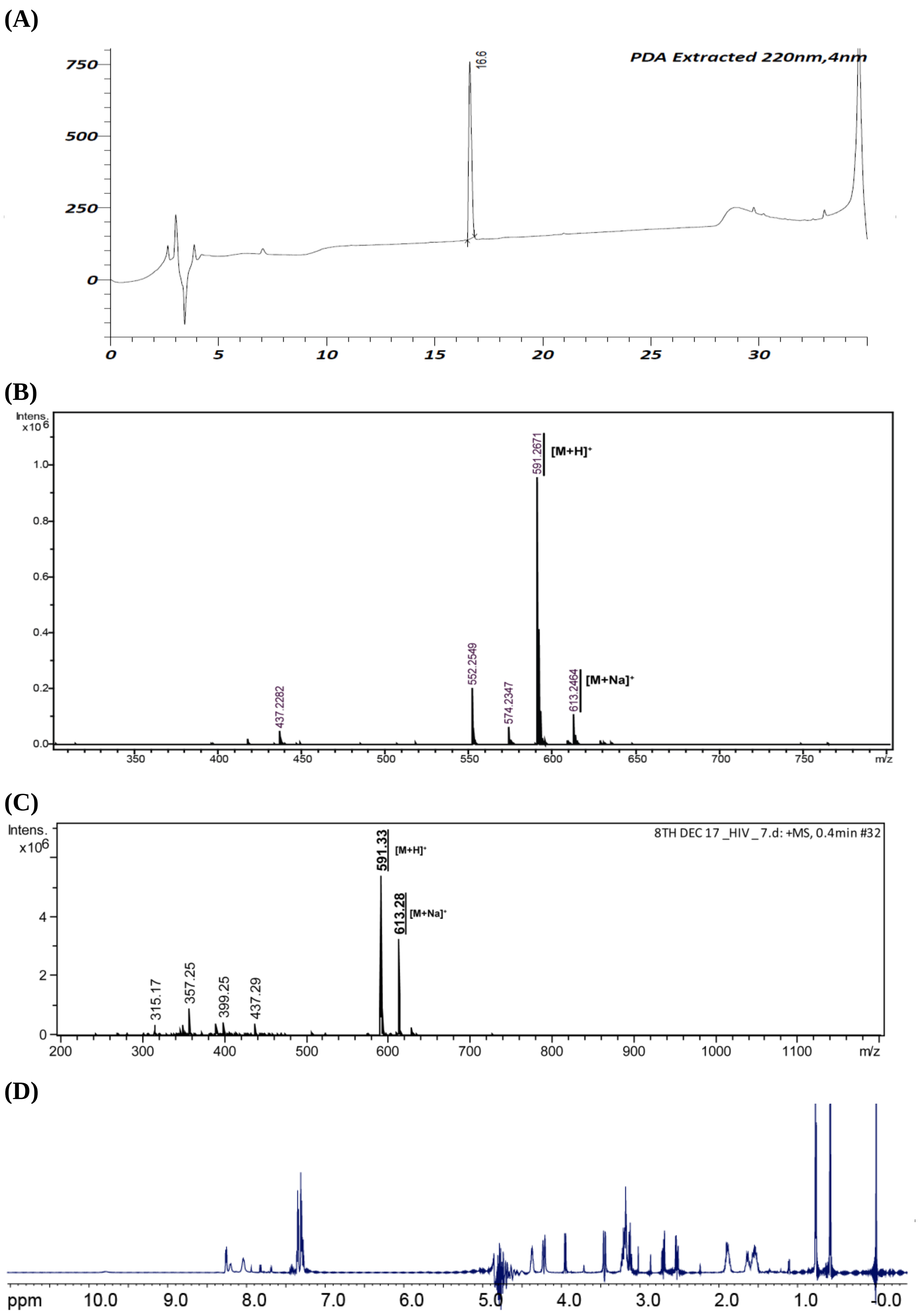

Figure S9 : (A) Analytical HPLC chromatogram of purified peptide **7,** (B MALDI (HRMS) profile. The lower molecular weight peak is due to unusual fragmentation pattern. (C) ESI-MS profile. (D) One dimensional ^{1}H NMR of pure peptide.

(A)

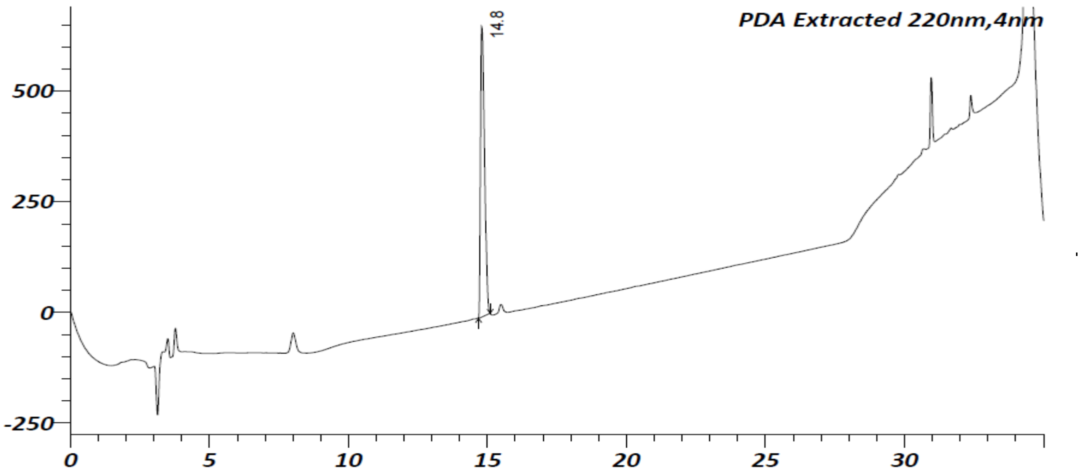

(B)

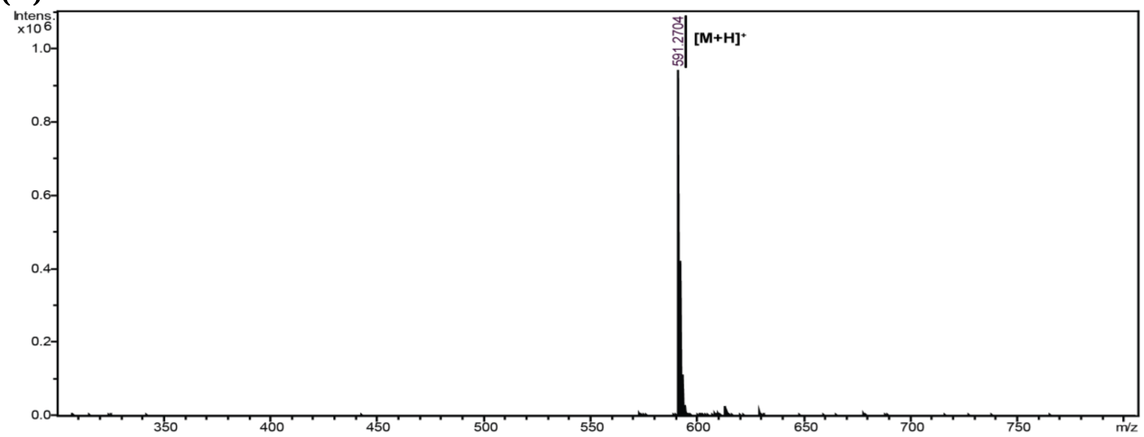

(C)

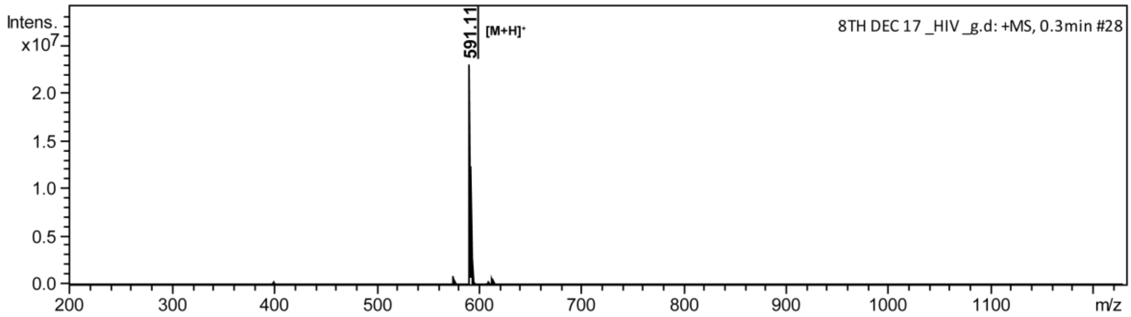

(D)

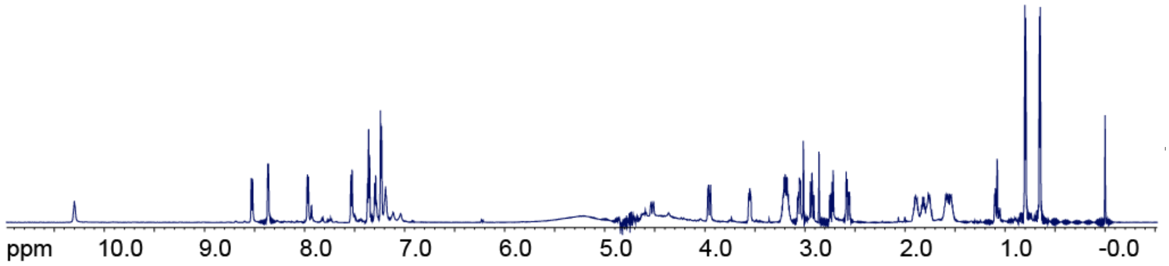

Figure S10 : (A) Analytical HPLC chromatogram of purified peptide **9,** (B) MALDI (HRMS) and (C) ESI-MS profile. (D) One dimensional ^{1}H NMR of pure peptide.

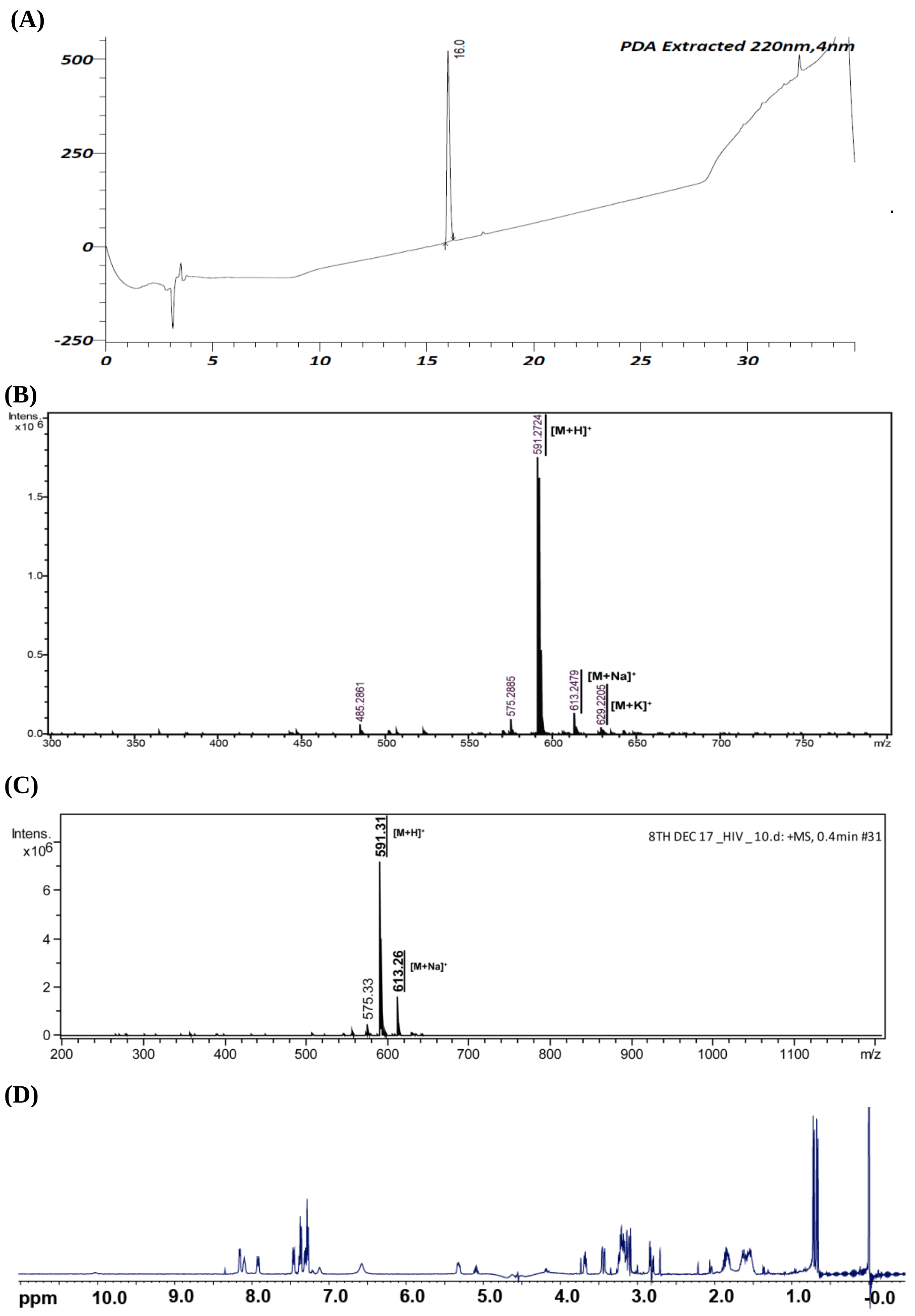

Figure S11 : (A) Analytical HPLC chromatogram of purified peptide **10,** (B) MALDI (HRMS) profile. The lower molecular weight peak is due to unusual fragmentation pattern. (C) ESI-MS profile. (D) One dimensional ^{1}H NMR of pure peptide.

(A)

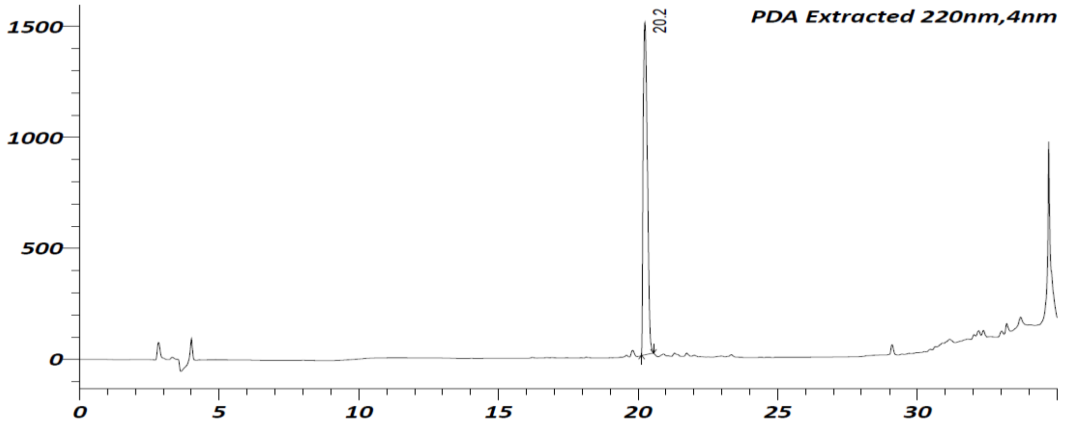

(B)

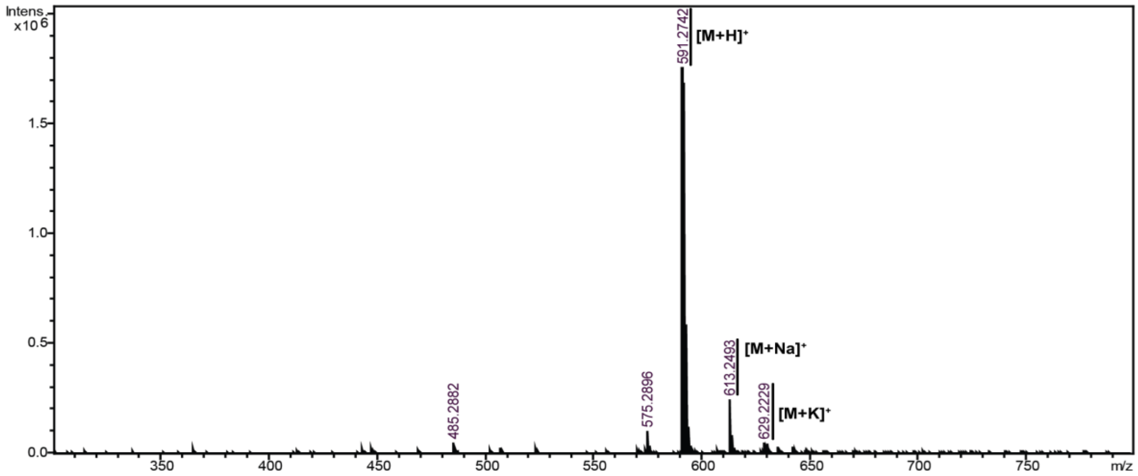

(C)

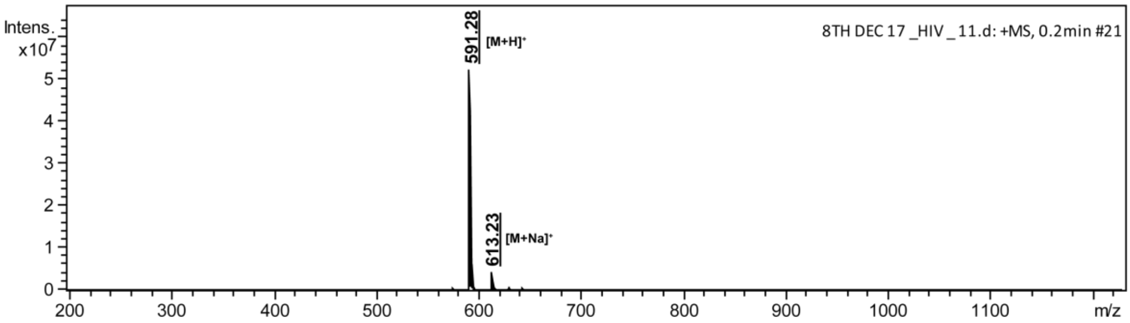

(D)

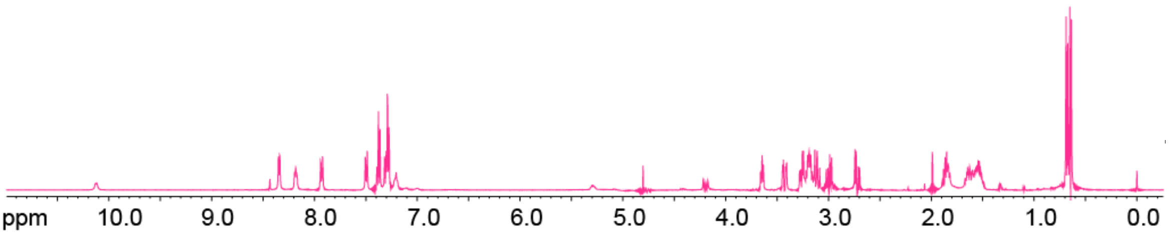

Figure S12 : (A) Analytical HPLC chromatogram of purified peptide **11,** (B) MALDI (HRMS) profile. The lower molecular weight peak is due to unusual fragmentation pattern. (C) ESI-MS profile. (D) One dimensional ^{1}H NMR of pure peptide.

(A)

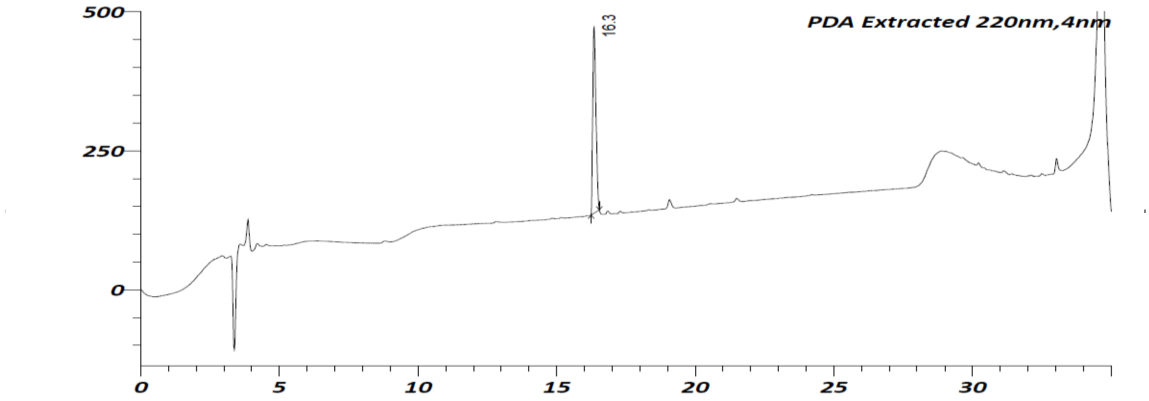

(B)

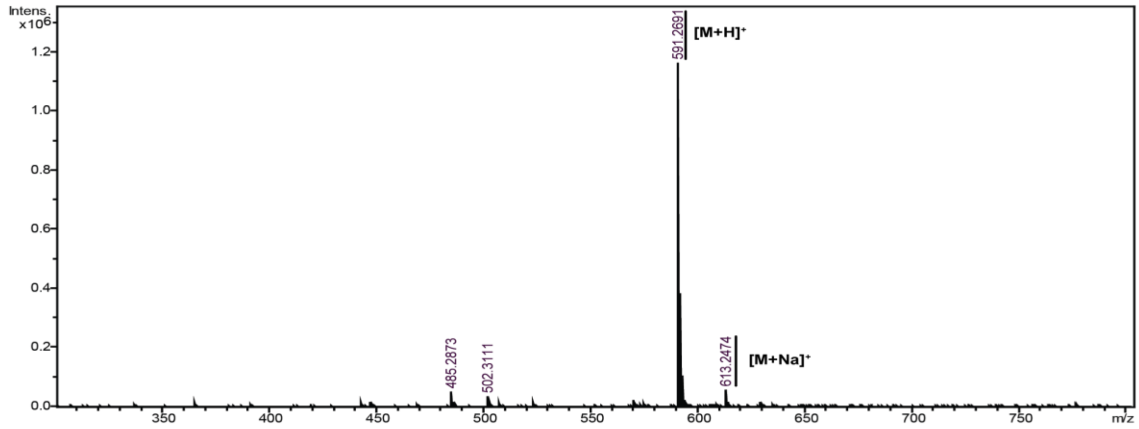

(C)

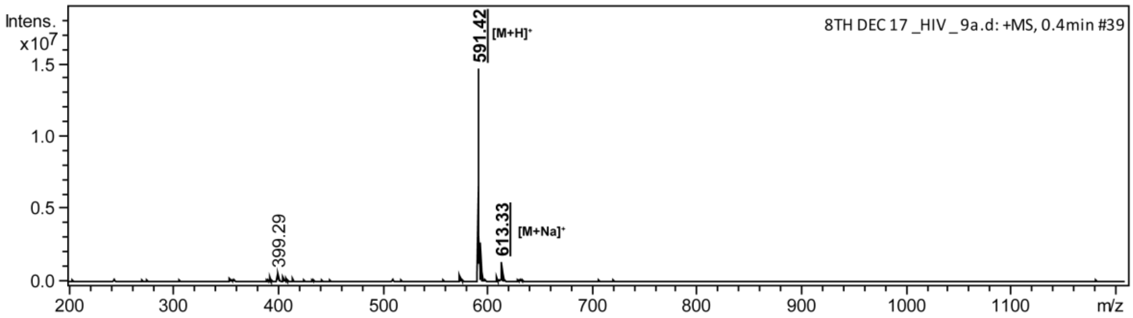

(D)

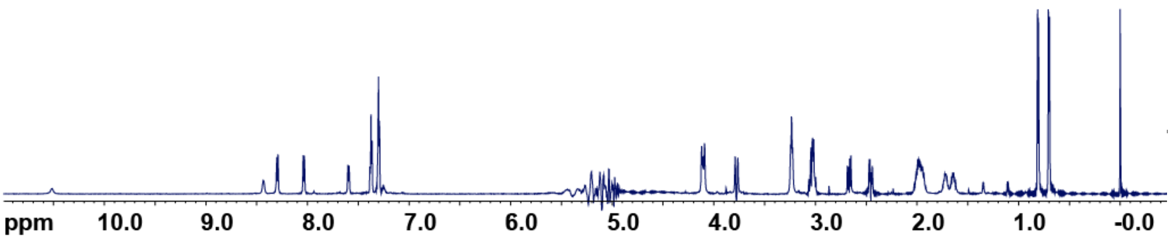

Figure S13 : (A) Analytical HPLC chromatogram of purified peptide **9a,** (B) MALDI (HRMS) profile. The lower molecular weight peak is due to unusual fragmentation pattern. (C) ESI-MS profile. (D) One dimensional ^{1}H NMR of pure peptide.

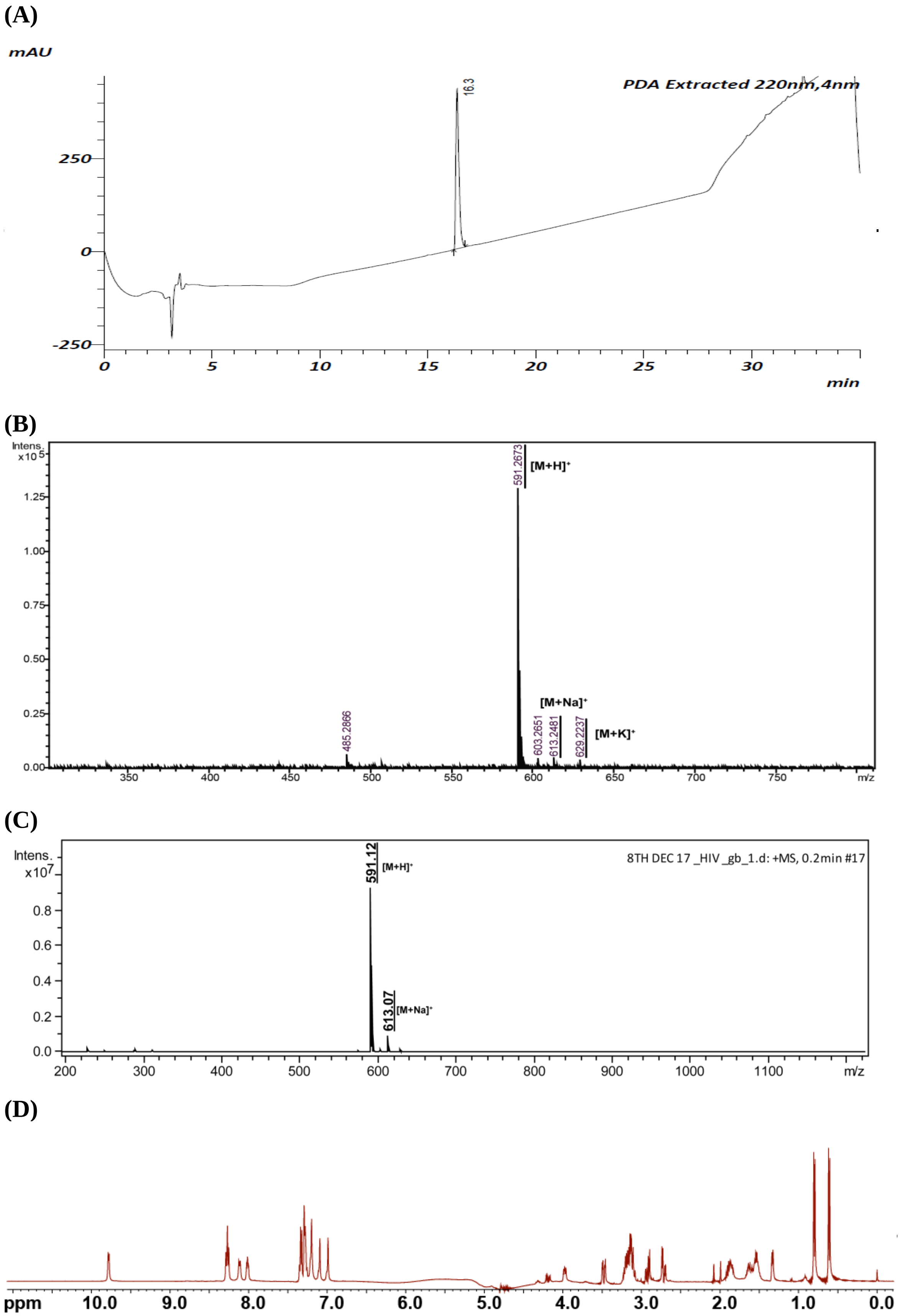

Figure S14 : (A) Analytical HPLC chromatogram of purified peptide **9b,** (B) MALDI (HRMS) profile. The lower molecular weight peak is due to unusual fragmentation pattern. (C) ESI-MS profile. (D) One dimensional ^{1}H NMR of pure peptide.

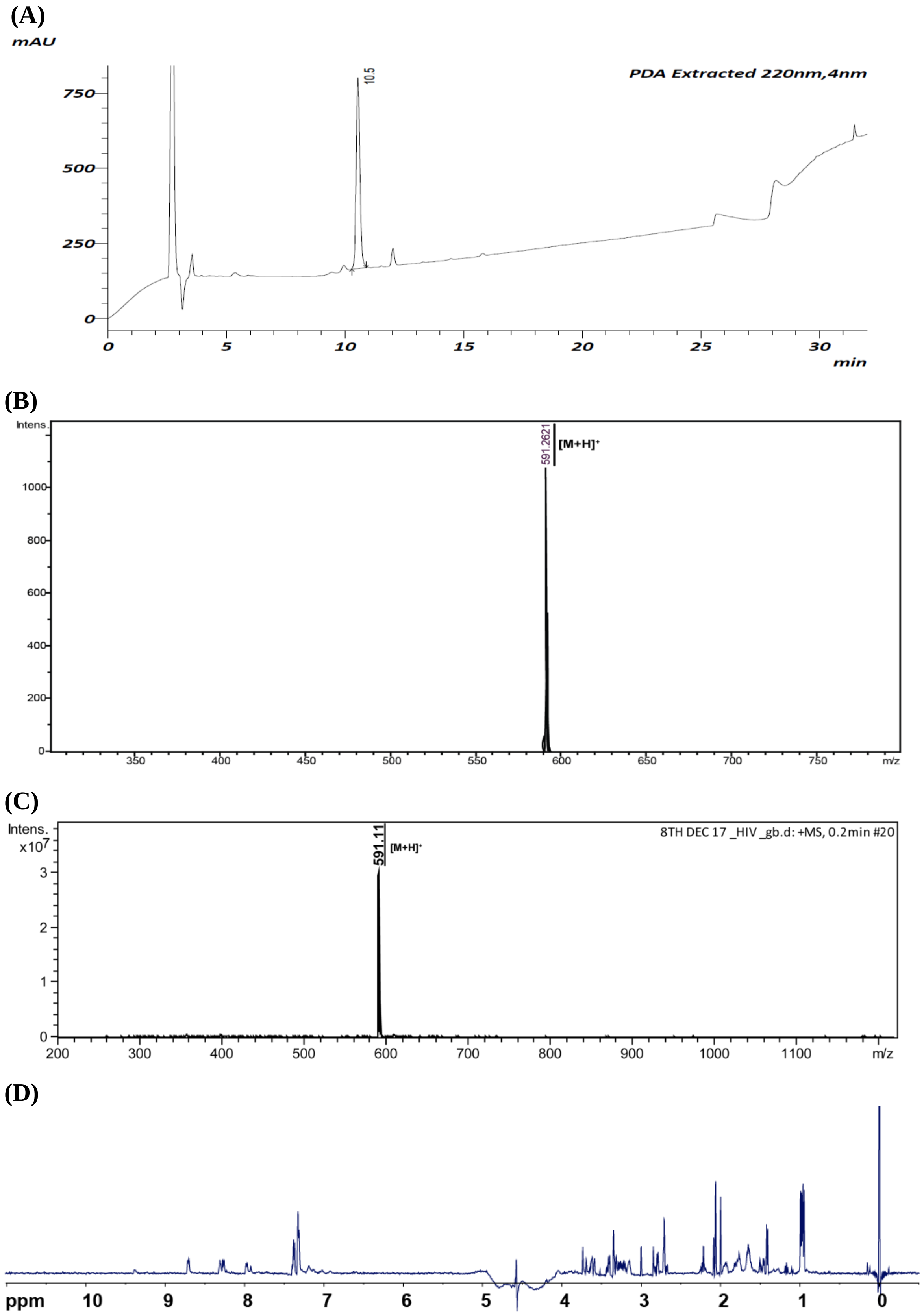

Figure S15 : (A) Analytical HPLC chromatogram of purified peptide **9c,** (B) MALDI (HRMS) (C) ESI-MS profile. (D) One dimensional ^{1}H NMR of pure peptide.

(A)

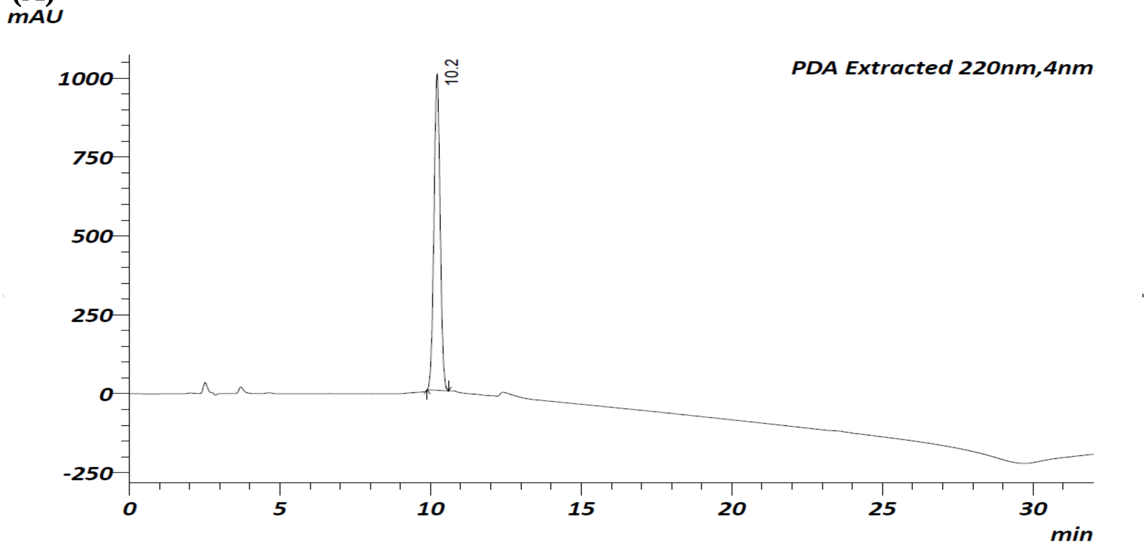

(B)

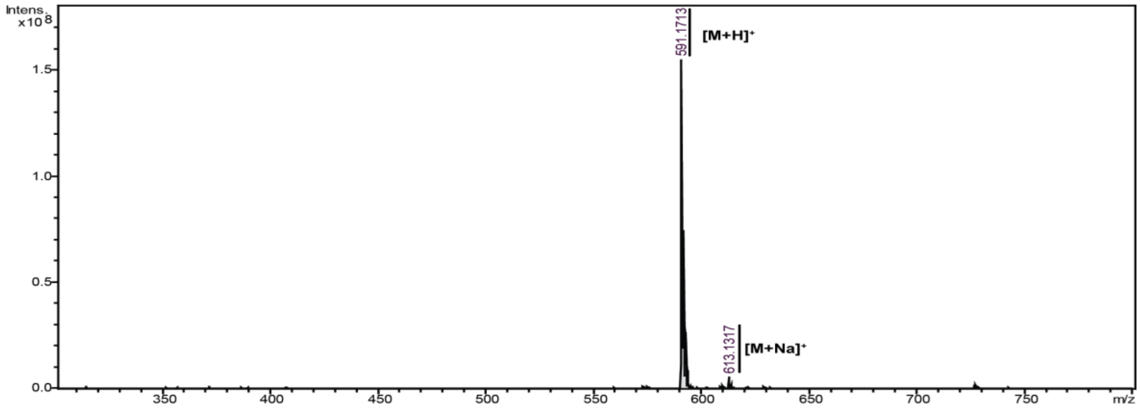

(C)

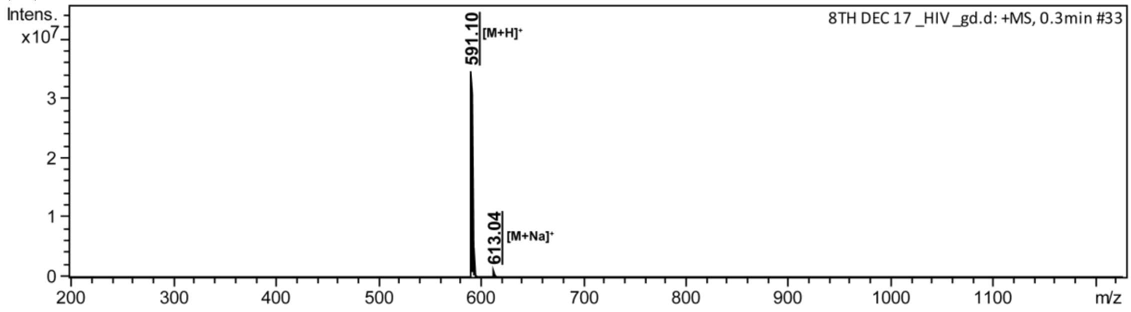

(D)

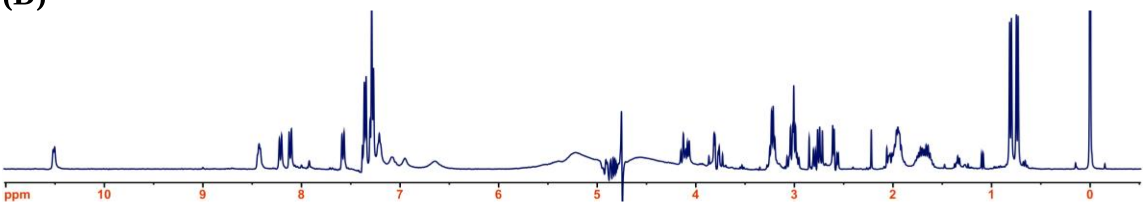

Figure S16 : (A) Analytical HPLC chromatogram of purified peptide **9d,** (B) MALDI (HRMS) (C) ESI-MS profile. (D) One dimensional ^{1}H NMR of pure peptide.

(A)

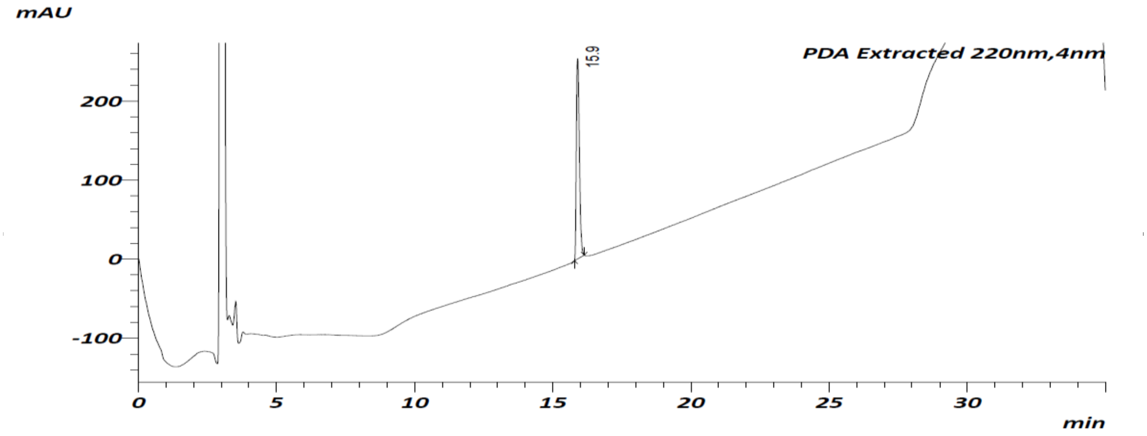

(B)

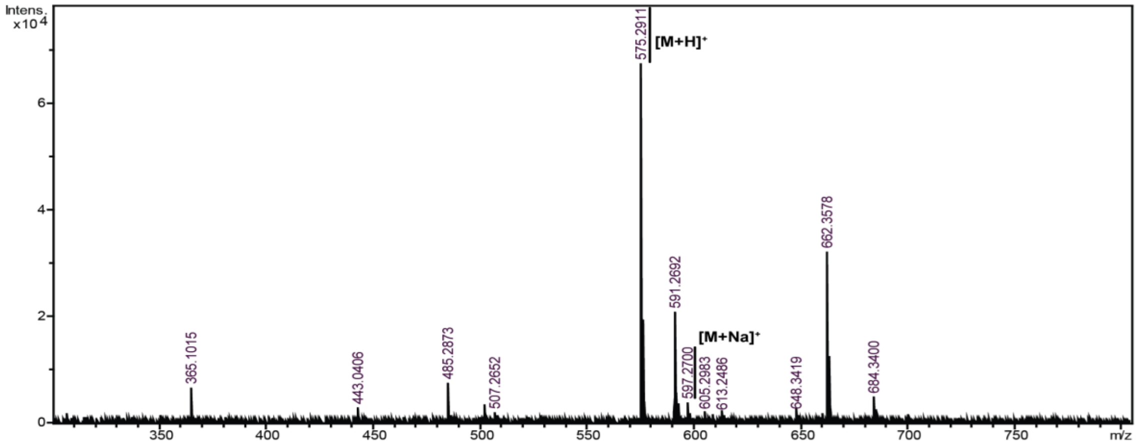

(C)

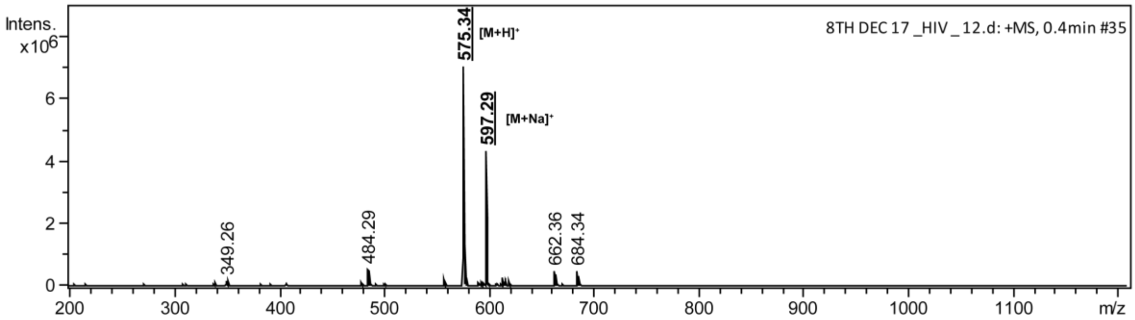

(D)

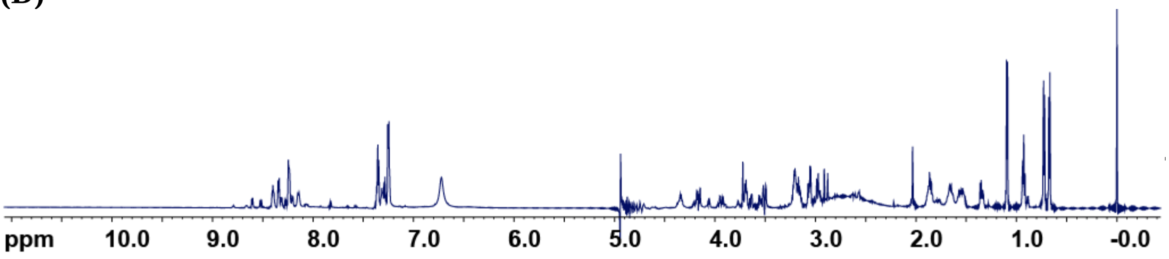

Figure S17 : (A) Analytical HPLC chromatogram of purified peptide **12,** (B) MALDI (HRMS) profile. The higher and lower molecular weight peak is due to unusual fragmentation pattern. (C) ESI-MS profile. (D) One dimensional ^{1}H NMR of pure peptide.

Table S3 : Absolute chemical shifts of **P** to **5**

	Ala1			Ala2			Ala3			Ala4			Ala5		
	HN	Hα	Hβ	HN	Hα	Hβ	HN	Hα	Hβ	HN	Hα	Hβ	HN	Hα	Hβ
**P**	8.49	4.17	1.14	8.36	4.11	1.24	7.69	4.24	1.16	8.04	3.90	1.30	7.61	4.25	1.29
**1**	8.77	4.56	1.30	10.41	4.74	1.36	7.52	4.22	1.28	8.06	3.82	1.32	7.54	4.26	1.15
**2**	8.38	4.17	1.15	8.62	4.49	1.42	9.20	5.10	1.39	8.25	3.81	1.31	7.50	4.29	1.17
**3**	7.99	4.28	1.14	7.91	4.12	1.24	8.16	4.68	1.36	9.54	4.67	1.32	8.42	3.98	1.22
**4**	8.94	4.19	1.16	8.60	4.12	1.25	7.41	4.33	1.30	8.33	4.38	1.44	9.39	4.76	1.27
**5**	10.43	4.96	1.26	8.35	4.10	1.21	7.41	4.19	1.26	8.03	3.91	1.26	7.59	4.63	1.29

Table S4 : Coupling constant ($_3J^{HNH\alpha}$) of **P-5** and their respective amide temperature coefficient (ppb/K) is shown in parantheses

	Ala1	Ala2	Ala3	Ala4	Ala5
**P**	8.2(-5.0)	8.2(-4.5)	8.3(0)	6.8(-6.5)	8.3(-3.0)
**1**	6.4(-6.5)	7.8(-6.0)	8.3(-0.5)	6.4(-3.0)	7.9(0.5)
**2**	6.1(-5.5)	7.2(-5.5)	8.4(-1.5)	5.9(-2.0)	8.5(-0.5)
**3**	7.4(-2.0)	7.4(-5.0)	8.5(-3.0)	7.2(-2.0)	7.2(-4.5)
**4**	6.5(-5.5)	7.9(-6.5)	8.8(-1.0)	6.5(-1.5)	7.7(0.5)
**5**		7.9(-6.5)	8.2(0)	6.6(-3.0)	7.4(0)

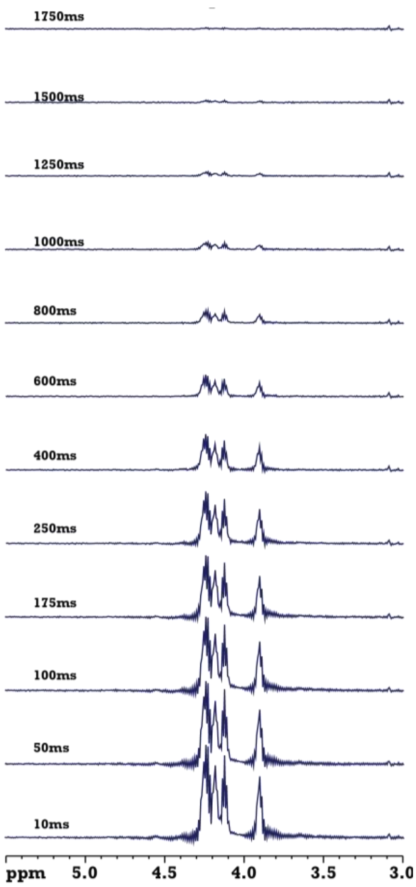

Figure S18 : 1-D HSQC of **P** acquired at noted T_1.

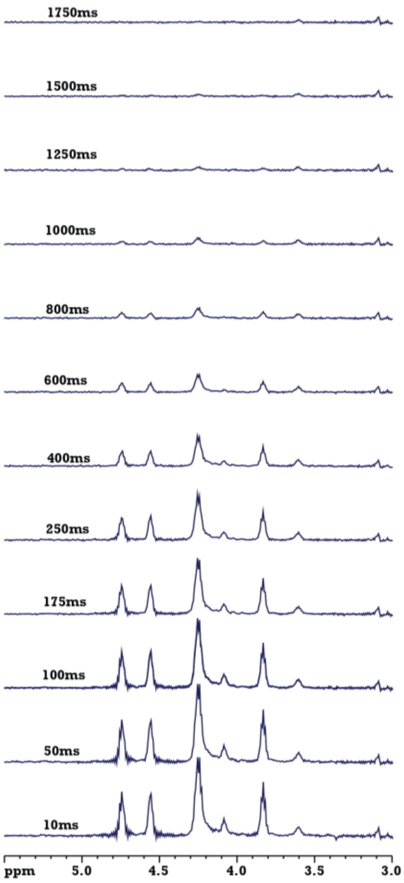

Figure S19 : 1-D HSQC of **1** acquired at noted T_1.

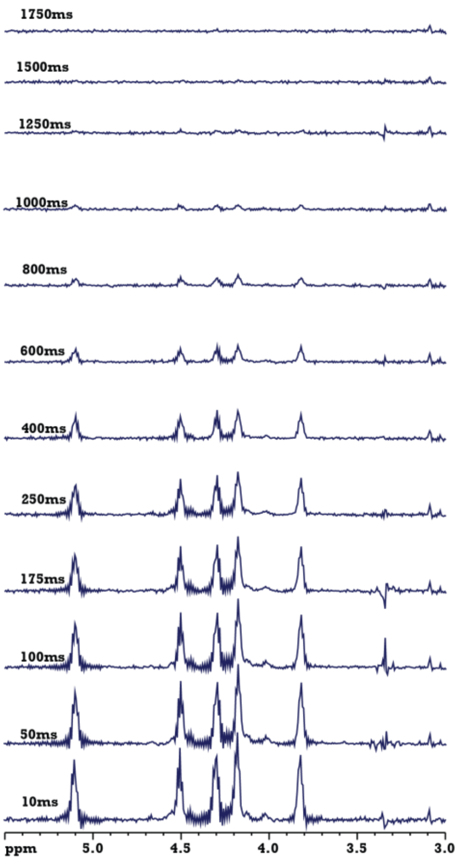

Figure S20 : 1-D HSQC of **2** acquired at noted T_1.

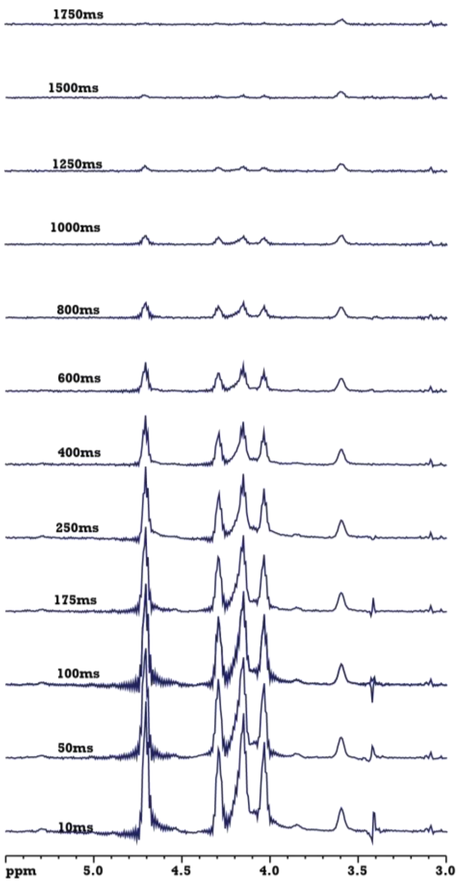

Figure S21 : 1-D HSQC of **3** acquired at noted T_1.

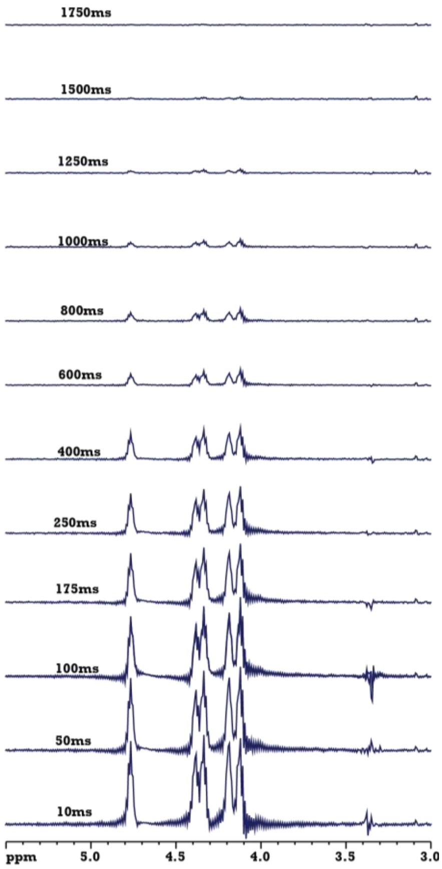

Figure S22 : 1-D HSQC of **4** acquired at noted T_1.

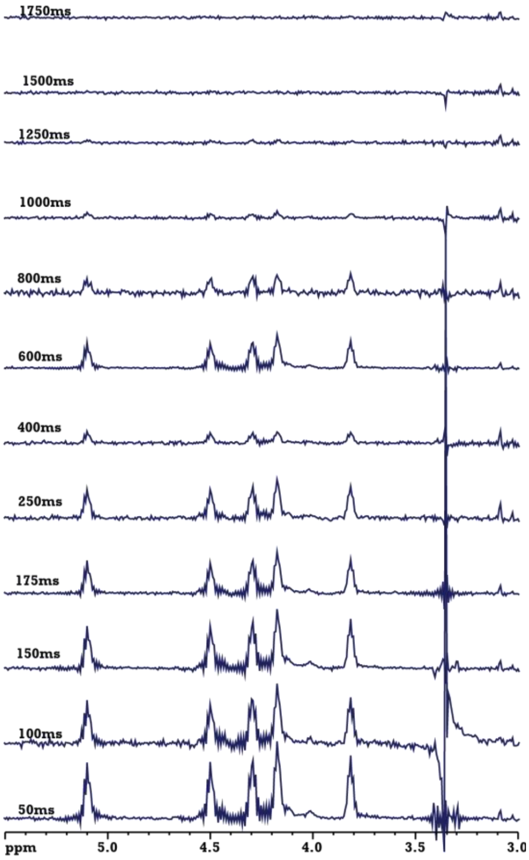

Figure S23 : 1-D HSQC of **5** acquired at noted T_1.

Table S5 : Absolute chemical shift of **6** and **12** in their major and minor conformers.

		6-Major Conformer	6-Minor Conformer	12-Major Conformer	12-Minor Conformer
Phe1	HN	8.24	8.11	8.33	8.19
	HA	4.68	5.06	4.69	5.05
	HB	3.04/2.99	3.06/2.99	3.05/2.98	3.05/2.95
Val2	HN	8.16	8.17	8.23	8.27
	HA	3.71	4.07	3.69	4.06
	HB	1.88	2.06	1.88	2.06
	HG	0.70/0.66	0.93/0.91	0.67/0.72	0.92
Arg3	HN	8.01	8.50	8.14	8.59
	HA	4.36	4.38	4.33	4.38
	HB	1.86	2.44	1.87	1.90
	HG	1.68/1.55	1.88/1.76	1.67/1.55	1.80/1.65
	HD	3.73	3.21	3.20	3.21
Gly4	HN	8.32	8.42	8.39	8.79
	HA	4.17/3.51	3.93	4.16/3.51	4.06/3.61
Asp5	HN	8.13	8.22	8.23	8.66
	HA	4.68	4.58	4.73	4.49
	HB	2.68/2.57	3.02/2.50	2.79/2.62	2.56/2.43

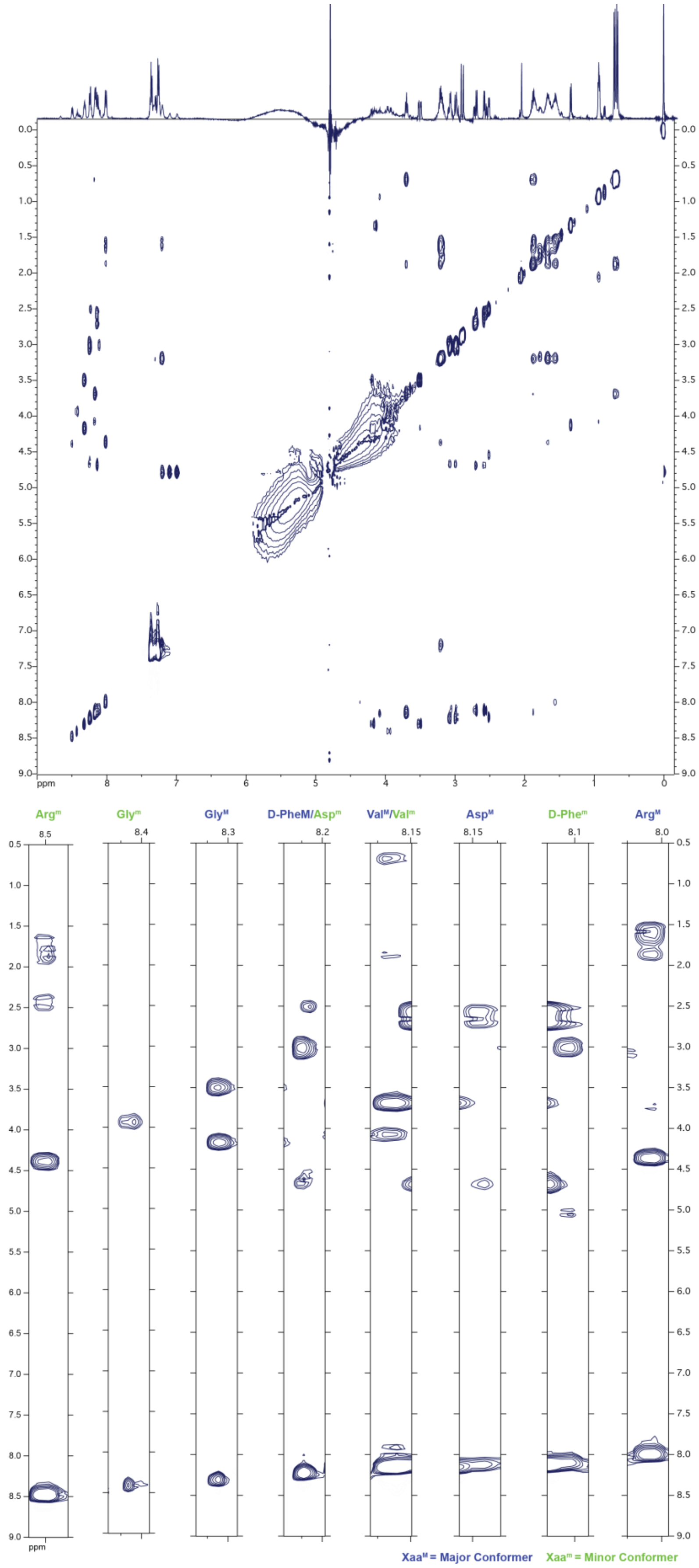

Figure S24 : [1]H-TOCSY of **6** showing two distinct conformers. Identified residues are shown in the form of strip plot.

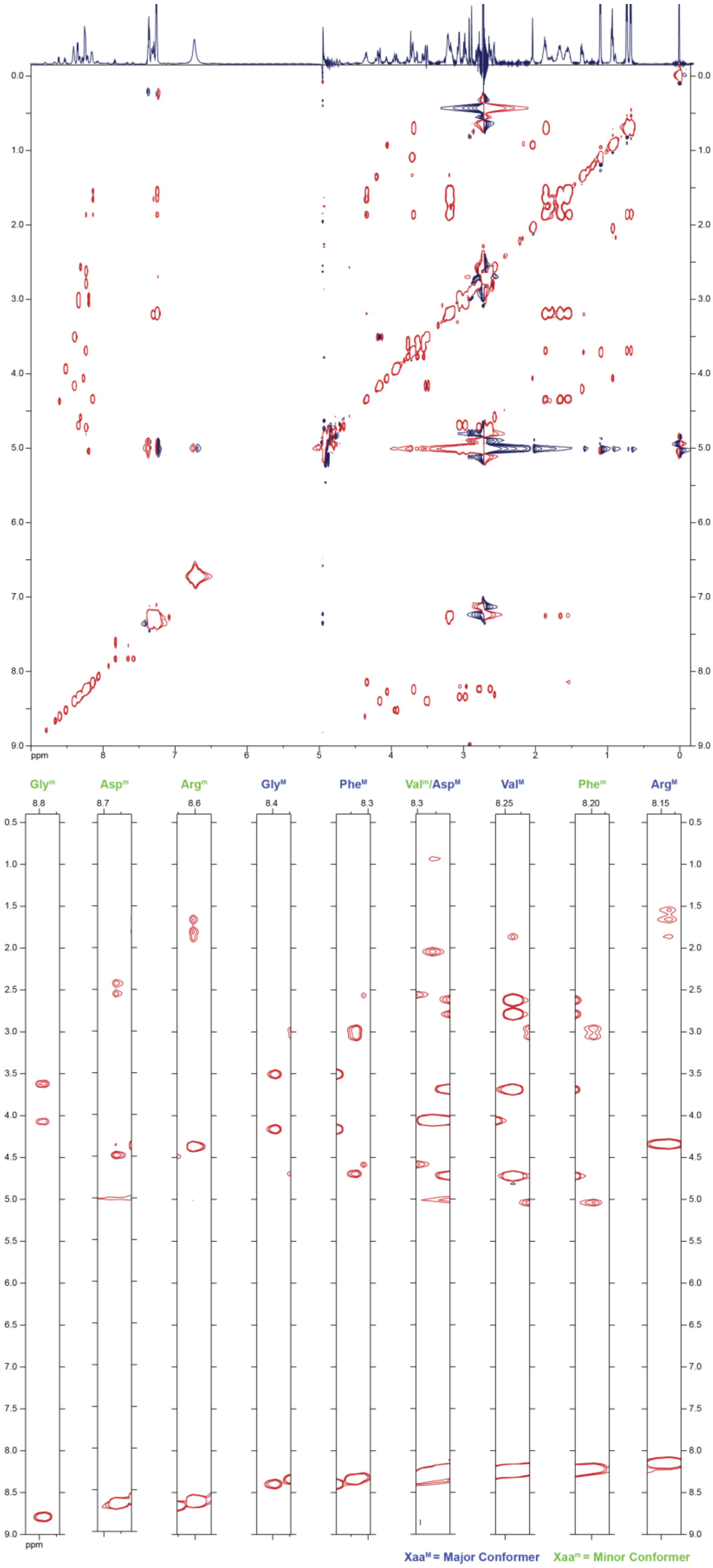

Figure S25 : [1]H-TOCSY of **12** showing two distinct conformers. Identified residues are shown in the form of strip plot.

Table S6 : Absolute chemical shift of **7-11**.

		7	9	9a	9b	9c	9d	10	11
Phe1	HN	8.26	7.97	8.04	8.25	7.96	10.49	7.91	10.11
	HA	4.76	4.6	4.77	5.03	4.63	4.80	5.07	5.29
	HB	2.83/2.66	3.05/2.93	3.03	3.12	2.77/2.69	2.84/2.54	3.12/2.81	3.25/3.11
	HD	7.30	7.21	7.29	7.27	7.26	7.29	7.26	7.29
Val2	HN	9.78	8.37	8.24	9.78	8.27	8.20	8.14	8.34
	HA	3.98	3.54	4.11	3.97	4.18	4.11	3.68	3.66
	HB	1.88	1.83	1.99	1.89	2.22	2.00	1.84	1.86
	HG	0.78/0.58	0.81/0.66	0.82/0.71	0.79/0.6	0.97/0.95	0.81/0.74	0.69/0.66	0.68
Arg3	HN	8.11	7.52	10.5	8.11	8.71	7.58	7.45	7.49
	HA	4.32	4.61	4.85	4.31	4.07	4.65	4.39	4.41
	HB	1.85	1.89	1.95	1.86	1.92	1.97	1.85	1.85
	HG	1.60/1.51	1.76/1.56	1.69	1.57/1.63	1.79/1.64	1.69	1.63/1.53	1.63/1.56
	HD	3.17	3.18	3.24	3.18		3.22	3.2	3.19
Gly4	HN	8.01	10.31	8.41	8	8.3	8.40	8.09	8.18
	HA	4.18/3.47	4.51/3.95	4.12/3.77	4.18/3.48	4.21/3.58	4.12/3.75	4.17/3.46	4.19/3.42
Asp5	HN	8.24	8.55	7.58	8.27	9.37	8.08	10.03	7.92
	HA	5.03	4.75	4.69	4.8	4.73	4.59	4.55	5.07
	HB	3.13	2.77/2.60	2.70/2.50	2.93/2.72		3.00/2.95	3.2	2.99/2.72

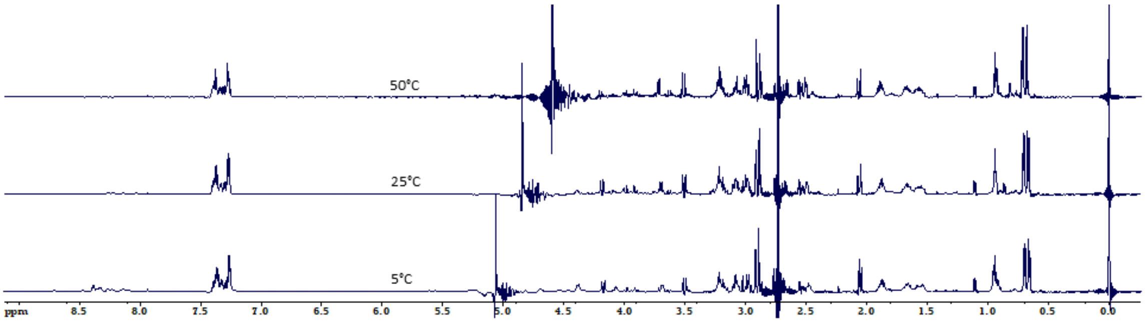

Figure S26 : ^{1}H overlay of **6** acquired at three different temperature (5, 25 and 50°C).

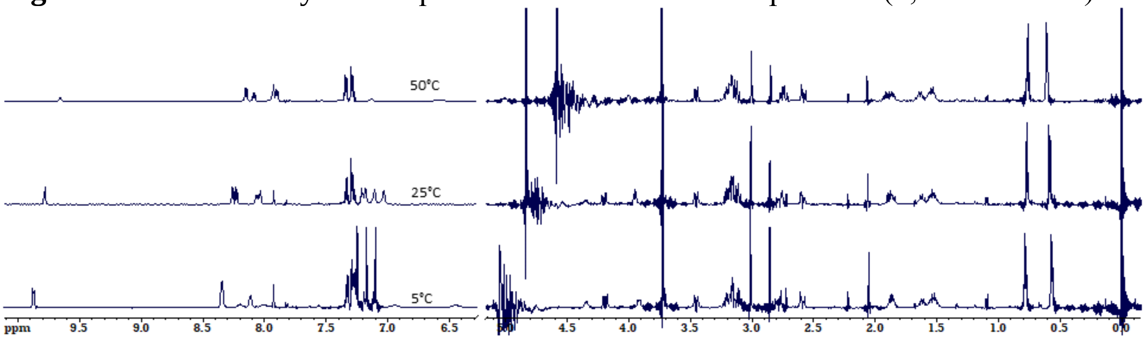

Figure S27 : ^{1}H overlay of **7** acquired at three different temperature (5, 25 and 50 °C).

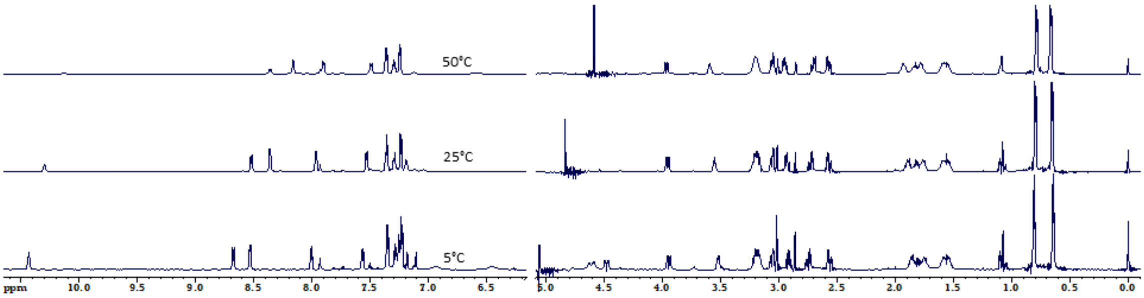

Figure S28 : ^{1}H overlay of **9** acquired at three different temperature (5, 25 and 50 °C).

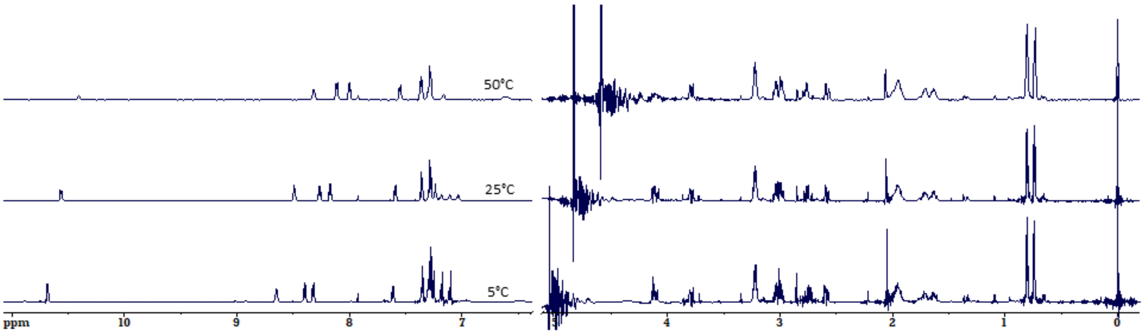

Figure S29 : [1]H overlay of **9a** acquired at three different temperature (5, 25 and 50 °C).

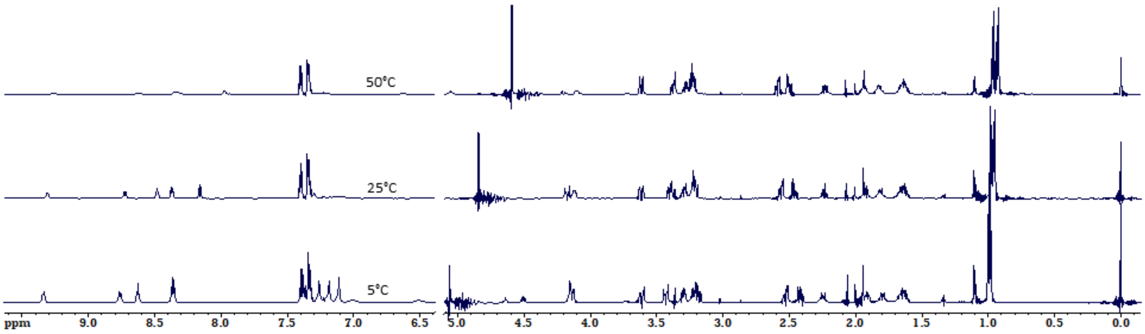

Figure S30 : [1]H overlay of **9b** acquired at three different temperature (5, 25 and 50 °C).

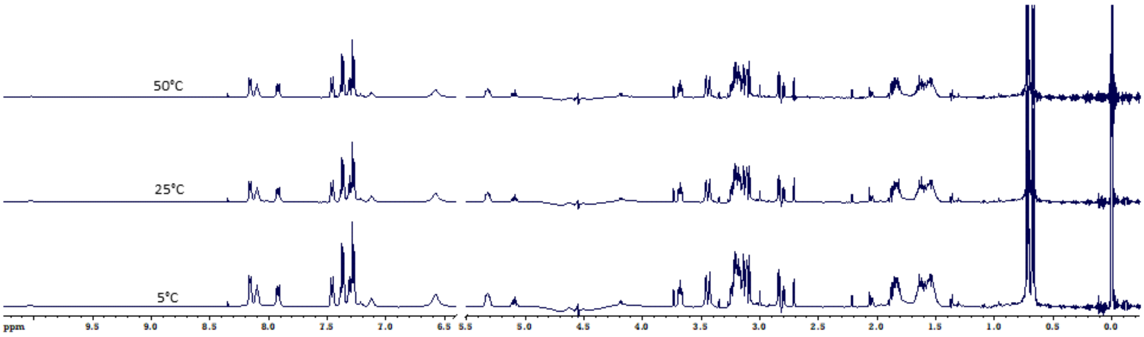

Figure S31 : [1]H overlay of **9c** acquired at three different temperature (5, 25 and 50 °C).

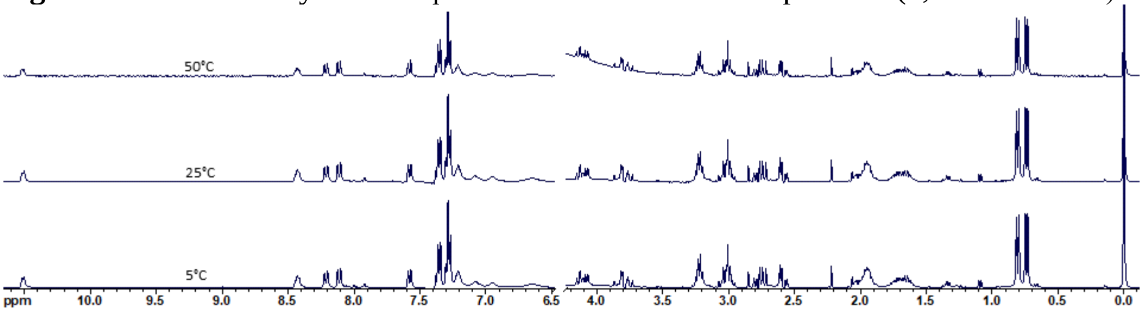

Figure S32 : [1]H overlay of **9d** acquired at three different temperature (5, 25 and 50 °C).

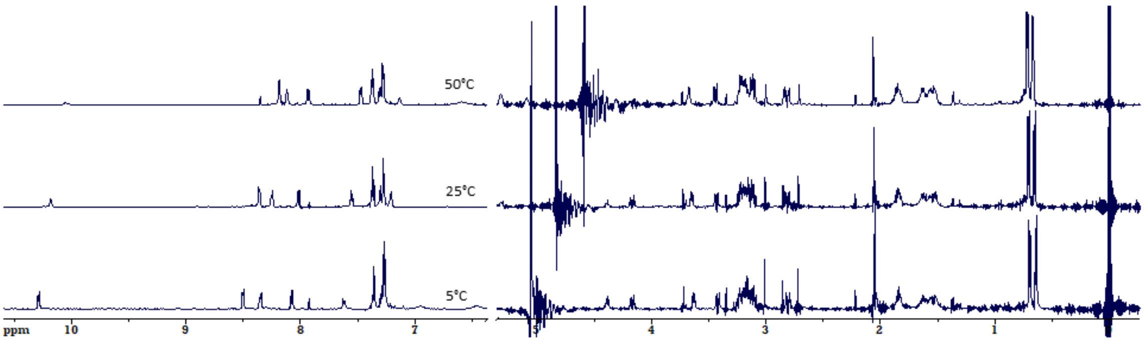

Figure S33 : ^{1}H overlay of **10** acquired at three different temperature (5, 25 and 50 °C).

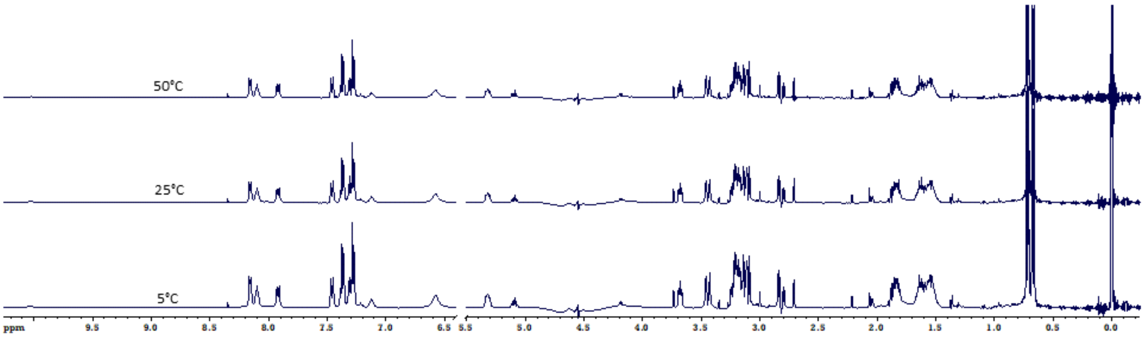

Figure S34 : ^{1}H overlay of **11** acquired at three different temperature (5, 25 and 50 °C).

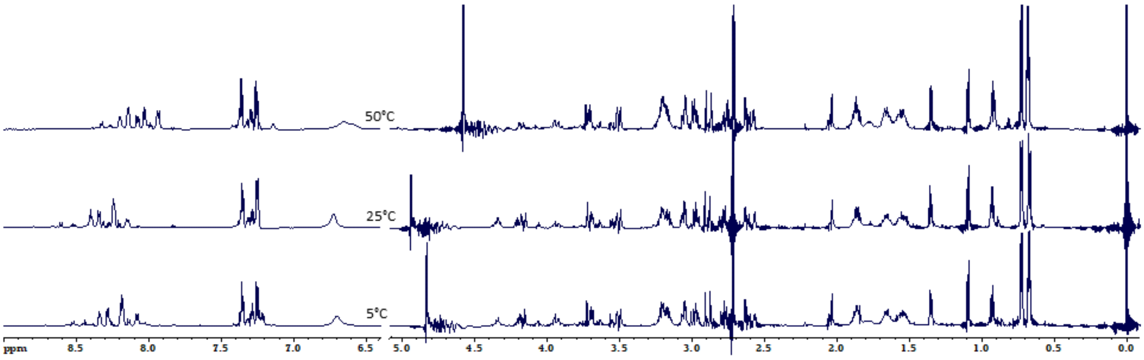

Figure S35 : ^{1}H overlay of **12** acquired at three different temperature (5, 25 and 50 °C).

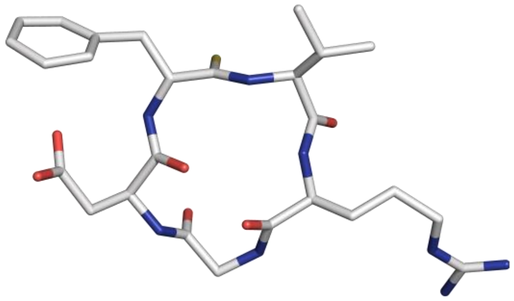

Figure S36 : Stereo view of **7**.

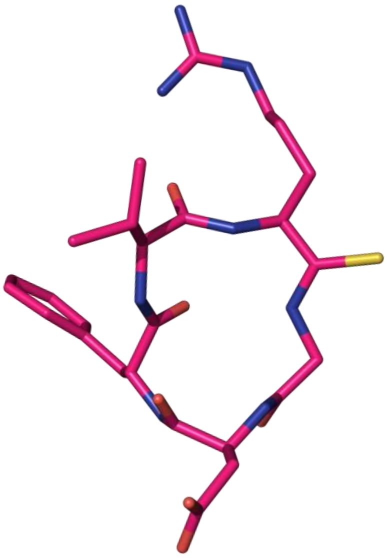

Figure S37 : Stereo view of **9**.

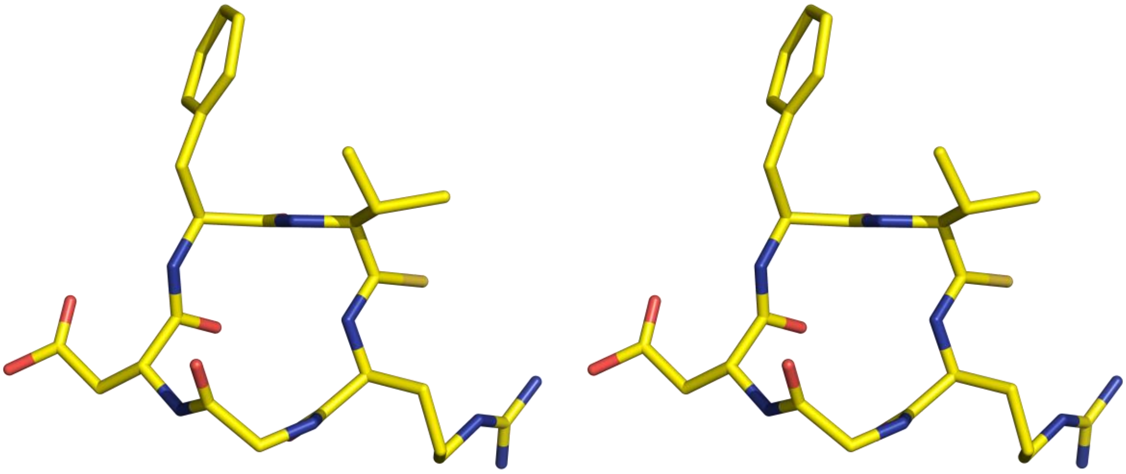

Figure S38 : Stereo view of **9a**.

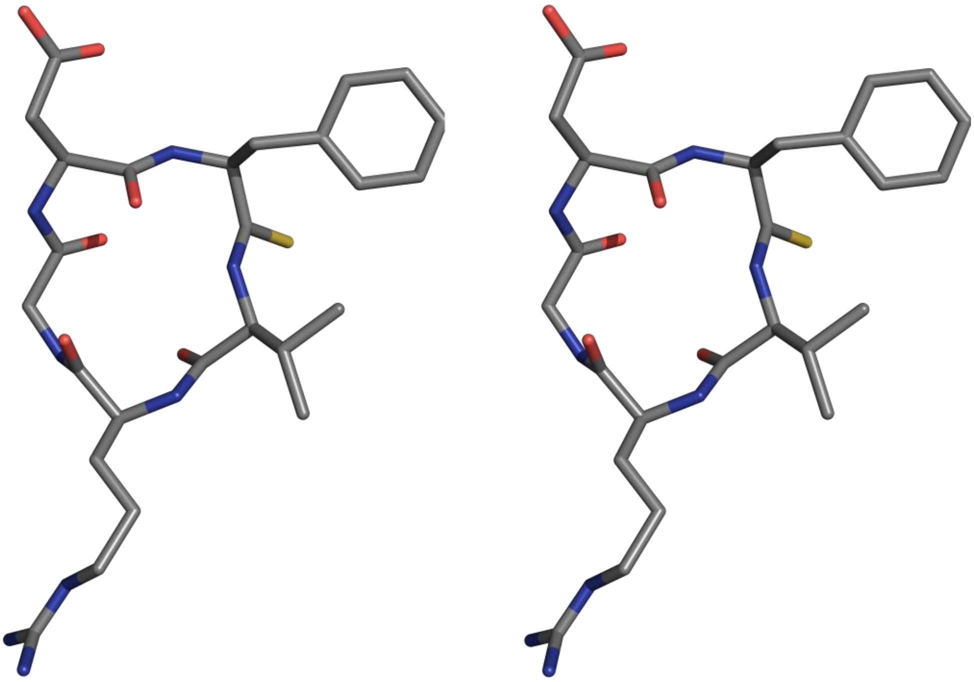

Figure S39 : Stereo view of **9b**.

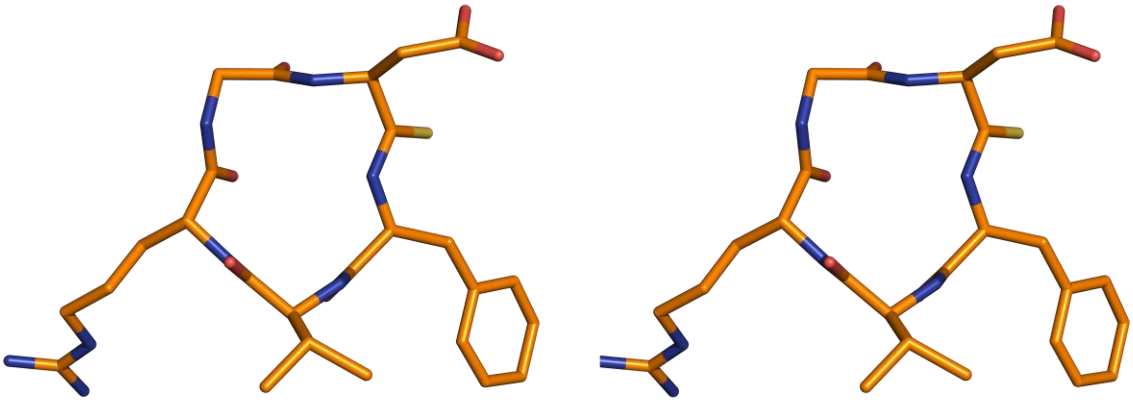

Figure S40 : Stereo view of **9c**.

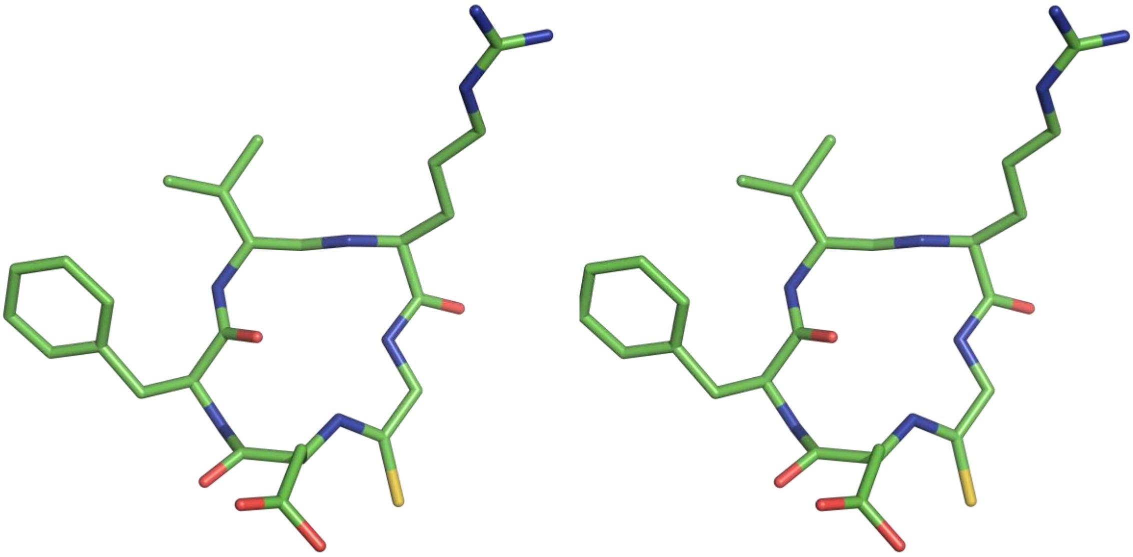

Figure S41 : Stereo view of **9d**.

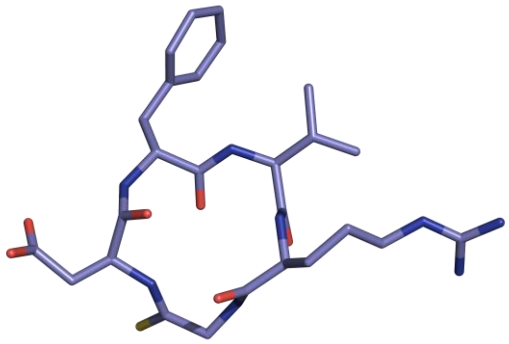

Figure S42 : Stereo view of **10**.

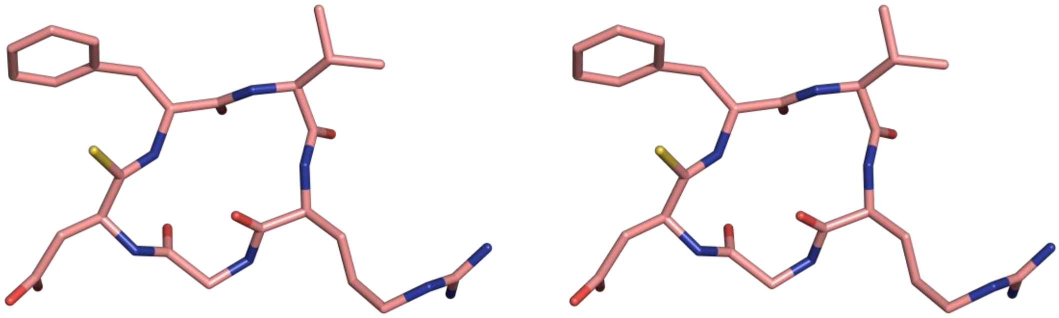

Figure S43 : Stereo view of **11**.

Table S7 : List of NOE's taken for structure calculation of **P** and the violation observed.

Interaction	NOE Distance	Lower Limit	Upper Limit	Violation
**Ala1HB-HA**	2.19	1.97	2.81	0.00
**Ala1HN-HB**	2.96	2.66	3.26	0.00
**Ala1HN-HA**	2.95	2.66	3.25	0.00
**Ala1HN-Ala5HA**	2.89	2.60	3.18	-0.21
**Ala2HA-Ala3HN**	2.05	1.85	2.26	0.39
**Ala2HA-HB**	2.19	1.97	2.41	0.12
**Ala2HN-HB**	2.95	2.66	3.25	0.00
**Ala2HN-Ala1HA**	2	1.80	2.20	0.00
**Ala2HN-HA**	2.73	2.46	3.00	0.00
**Ala3HA-HN**	2.64	2.38	2.90	0.03
**Ala4HN-Ala3HA**	3.32	2.99	3.65	0.00
**Ala2HN-Ala3HN**	3.61	3.25	3.97	-0.46
**Ala4HN-HA**	3.18	2.86	3.50	0.00
**Ala4HA-Ala5HN**	3.53	3.18	3.88	0.00
**Ala4HA-HB**	2.04	1.84	2.64	0.00
**Ala4HB-HN**	2.59	2.33	3.65	0.00
**Ala5HA-HN**	2.56	2.30	2.82	0.14
**Ala5HB-HN**	3.1	2.79	3.81	0.00
**Ala4HN-Ala5HN**	3.41	3.07	3.75	0.52

Table S8 : List of NOE's taken for structure calculation of **1** and the violation observed.

Interactions	NOE Distance	Lower Limit	UpperLimit	Violation
**Ala1HA-HB**	2.19	1.97	2.81	0.00
**Ala1HA-HN**	2.68	2.41	2.95	0.00
**Ala2HN-Ala1HA**	2.23	2.01	2.46	0.00
**Ala1HA-Ala3HN**	3.48	3.13	3.82	0.00
**Ala1HB-HN**	2.64	2.38	3.30	0.00
**Ala1HN-Ala5HA**	2.26	2.03	2.49	0.00
**Ala2HA-HB**	2.20	1.98	2.83	0.00
**Ala2HA-Ala3HN**	2.80	2.52	3.08	0.01
**Ala2HN-HA**	2.93	2.64	3.22	0.00
**Ala2HN-HB**	3.11	2.80	3.82	0.00
**Ala2HN-Ala3HN**	2.79	2.51	3.07	-0.61
**Ala3HA-HB**	2.22	2.00	2.84	0.00
**Ala3HA-Ala4HN**	2.61	2.35	2.87	0.49
**Ala4HA-Ala5HN**	2.88	2.59	3.17	0.26
**Ala4HB-HA**	2.14	1.93	2.75	0.00
**Ala5HA-HB**	2.18	1.96	2.80	0.00
**Ala5HN-HB**	2.80	2.52	3.48	0.00
**Ala4HN-Ala5HN**	2.48	2.23	2.73	-0.29
**Ala3HN-Ala4HN**	1.56	1.40	1.72	0.00

Table S9 : List of NOE's taken for structure calculation of **2** and the violation observed.

Interactions	NOE Distance	Lower Limit	Upper Limit	Violation
**Ala1HA-HB**	2.21	1.99	2.83	0.00
**Ala1HA-HN**	2.63	2.36	2.89	0.02
**Ala2HA-HB**	2.22	2.00	2.84	0.00
**Ala2HA-HN**	2.84	2.56	3.13	0.00
**Ala2HB-HN**	2.52	2.27	3.17	0.06
**Ala3HA-HB**	2.24	2.02	2.87	0.00
**Ala3HA-HN**	2.89	2.60	3.18	0.00
**Ala3HB-HN**	2.73	2.45	3.40	0.00
**Ala4HA-HB**	2.18	1.96	2.80	0.00
**Ala4HA-HN**	2.82	2.53	3.10	-0.25
**Ala5HA-HB**	2.14	1.93	2.76	0.00
**Ala5HA-HN**	2.72	2.45	3.00	0.00
**Ala5HB-HN**	2.95	2.66	3.65	0.00
**Ala1HA-Ala2HN**	2.34	2.10	2.57	0.00
**Ala1HN-Ala5HA**	2.52	2.27	2.78	-0.10
**Ala2HN-Ala3HN**	2.69	2.42	2.95	-0.28
**Ala3HA-Ala4HN**	3.04	2.73	3.34	-0.22
**Ala3HN-Ala5HN**	3.43	3.08	3.77	-0.15
**Ala4HA-Ala5HN**	3.30	2.97	3.63	0.00

**Ala4HN-Ala5HN**	2.83	2.55	3.12	0.07
**Ala4HB-Ala5HN**	2.98	2.68	3.68	0.00

Table S10 : List of NOE's taken for structure calculation of **3** and the violation observed.

Interactions	NOE Distance	Lower Limit	Upper Limit	Violation
**Ala1HA-HB**	2.22	2	2.84	0.00
**Ala1HA-HN**	2.73	2.46	3	0.00
**Ala1HB-HN**	2.80	2.52	3.48	0.00
**Ala2HA-HB**	2.21	1.99	2.83	0.00
**Ala2HA-HN**	2.74	2.47	3.01	-0.24
**Ala2HB-HN**	2.75	2.48	3.43	0.18
**Ala3HA-HB**	2.21	1.99	2.83	0.00
**Ala3HB-HN**	2.85	2.57	3.54	0.00
**Ala4HA-HB**	2.22	2	2.84	0.00
**Ala4HB-HN**	3.16	2.84	3.88	0.00
**Ala5HA-HB**	2.27	2.04	2.90	0.00
**Ala5HA-HN**	2.61	2.35	2.87	0.10
**Ala1HA-Ala2HN**	2.61	2.35	2.87	0.00
**Ala1HN-Ala5HA**	2.67	2.4	2.94	-0.13
**Ala1HN-Ala5HN**	3.16	2.84	3.48	0.00
**Ala2HA-Ala3HN**	3.16	2.84	3.48	0.00
**Ala2HB-Ala3HN**	2.84	2.56	3.52	0.00
**Ala2HN-Ala3HN**	3.34	3.01	3.67	0.22
**Ala3HB-Ala4HN**	2.81	2.53	3.5	0.44
**Ala3HN-Ala4HN**	2.74	2.47	3.01	-0.57
**Ala4HA-Ala5HN**	2.68	2.41	2.95	0.50
**Ala4HN-Ala5HN**	3.06	2.75	3.37	-0.38
**Ala4HB-Ala5HN**	3.06	2.75	3.77	0.00
**Ala5HN-HB**	2.90	2.61	3.59	0.00
**Ala4HA-HN**	2.94	2.65	3.24	0.00
**Ala3HA-HN**	2.63	2.37	2.89	0.07

Table S11 : List of NOE's taken for structure calculation of **4** and the violation observed.

Interactions	NOE Distance	Lower Limit	Upper Limit	Violation
**Ala1HA-HB**	2.18	1.96	2.80	0.00
**Ala1HA-HN**	2.84	2.56	3.12	0.00
**Ala1HB-HN**	2.60	2.34	3.26	0.00
**Ala2HA-HB**	2.16	1.94	2.78	0.00
**Ala2HA-HN**	2.76	2.48	3.04	0.00
**Ala2HB-HN**	2.67	2.40	3.34	0.00
**Ala3HA-HB**	2.10	1.89	2.71	0.00
**Ala3HA-HN**	2.74	2.47	3.01	0.00
**Ala3HB-HN**	2.87	2.58	3.56	0.00
**Ala4HA-HB**	2.15	1.94	2.77	0.00
**Ala4HB-HN**	2.66	2.39	3.33	0.00
**Ala5HA-HB**	2.18	1.96	2.80	0.00

**Ala5HA-HN**	2.96	2.66	3.26	0.00
**Ala5HB-HN**	3.34	3.01	4.07	0.00
**Ala1HA-Ala2HN**	2.20	1.98	2.42	0.00
**Ala1HN-Ala5HA**	2.35	2.12	2.59	0.00
**Ala1HN-Ala5HB**	3.51	3.16	4.26	0.00
**Ala2HA-Ala3HN**	2.99	2.69	3.29	-0.14
**Ala2HN-Ala3HN**	2.75	2.48	3.03	0.00
**Ala3HB-Ala4HN**	2.43	2.19	3.07	0.00
**Ala3HN-Ala4HN**	3.23	2.91	3.55	-0.61
**Ala3HN-Ala5HN**	3.37	3.03	3.71	0.00
**Ala4HA-Ala5HN**	3.88	3.49	4.27	0.00
**Ala4HN-Ala5HN**	2.60	2.34	2.86	-0.49
**Ala4HB-Ala5HN**	3.43	3.09	4.17	0.00

Table S12 : List of NOE's taken for structure calculation of **5** and the violation observed.

Interactions	NOE Distance	Lower Limit	Upper Limit	Violation
**Ala1HA-HB**	2.19	1.97	2.81	0.00
**Ala1HN-HA**	2.87	2.58	3.16	0.00
**Ala2HN-Ala1HA**	2.14	1.93	2.35	0.13
**Ala1HN-HB**	2.42	2.18	3.06	0.00
**Ala2HN-HA**	2.55	2.30	2.81	0.14
**Ala3HN-Ala2HA**	2.84	2.56	3.12	0.00
**Ala2HB-HA**	2.11	1.90	2.72	0.00
**Ala2HN-HB**	2.40	2.16	3.04	0.17
**Ala3HN-HA**	2.68	2.41	2.95	0.00
**Ala4HN-Ala3HA**	2.75	2.48	3.03	0.12
**Ala3HA-HB**	2.22	2.00	2.84	0.00
**Ala3HN-HB**	2.41	2.17	3.05	0.20
**Ala2HN-Ala3HN**	2.48	2.23	2.73	-0.04
**Ala4HN-Ala3HN**	2.76	2.48	3.04	0.58
**Ala4HN-HA**	2.43	2.19	2.67	0.22
**Ala5HN-Ala4HA**	2.90	2.61	3.19	0.10
**Ala4HA-HB**	2.23	2.01	2.85	0.00
**Ala4HN-HB**	2.14	1.93	2.75	0.04
**Ala1HN-Ala5HA**	2.15	1.94	2.37	0.00
**Ala5HN-HA**	2.68	2.41	2.95	0.00
**Ala5HA-HB**	2.20	1.98	2.82	0.00
**Ala5HN-HB**	2.33	2.10	2.96	0.28
**Ala1HN-Ala5HN**	2.96	2.66	3.26	0.00
**Ala4HN-Ala5HN**	2.50	2.25	2.75	0.00

Table S13 : List of NOE's taken for structure calculation of **7** and the violation observed.

Interaction	NOE Distance	Lower Limit	Upper Limit	Violation
**Phe1HA-HB2**	1.73	1.56	1.90	0.43
**Phe1HA-HD1**	2.58	2.32	2.84	0.16
**Phe1HA-HN**	3.02	2.72	3.32	0.00
**Phe1HA-Val2HN**	2.25	2.03	2.48	0.00

**Phe1HB2-HD2**	2.21	1.99	2.43	0.24
**Val2HN-HA**	3.01	2.71	3.31	0.00
**Val2HA-HB**	2.62	2.36	2.88	0.00
**Val2HN-HB**	2.83	2.55	3.11	0.43
**Val2HA-HG1**	2.72	2.45	2.99	0.00
**Val2HB-HG1**	2.37	2.13	2.61	0.00
**Val2HN-HG1**	3.29	2.96	3.62	0.21
**Val2HA-HG2**	2.66	2.39	2.93	0.41
**Val2HB-HG2**	2.3	2.07	2.53	0.00
**Arg3HA-HG1**	1.9	1.71	2.09	0.30
**Arg3HA-HN**	2.94	2.65	3.23	0.00
**Gly4HN-Arg3HA**	2.52	2.27	2.77	0.00
**Arg3HN-Val2HA**	2.9	2.61	3.19	0.19
**Arg3HN-Val2HN**	3.52	3.17	3.87	-0.43
**Gly4HN-HA2**	3.19	2.87	3.51	-0.15
**Gly4HA2-Asp5HN**	2.55	2.30	2.81	0.19
**Gly4HA1-HA2**	2.01	1.81	2.21	-0.04
**Gly4HN-HA1**	2.51	2.26	2.76	0.00
**Asp5HN-Gly4HA1**	3.24	2.92	3.56	-0.37
**Asp5HA-HN**	2.14	1.93	2.35	0.08
**Asp5HN-HB1**	3.08	2.77	3.39	0.00
**Asp5HB1-HB2**	1.86	1.67	2.05	0.00
**Asp5HN-HB2**	3.12	2.81	3.43	0.00
**Phe1HN-HB2**	2.51	2.26	2.76	0.10
**Arg3HA-HB1**	2.61	2.35	2.87	0.17
**Arg3HD2-HB1**	2.63	2.37	2.89	0.37
**Arg3HB2-HG1**	2.23	2.01	2.45	0.17
**Arg3HB1-HG2**	2.42	2.18	2.66	0.00
**Arg3HB1-HN**	2.44	2.20	2.68	0.00
**Arg3HD1-HG1**	1.84	1.66	2.02	0.48

Table S14 : List of NOE's taken for structure calculation of **9** and the violation observed.

Interaction	NOE Distance	Lower Limit	Upper Limit	Violation
**Phe1HA-HN**	2.52	2.27	2.77	0.00
**Phe1HA-Val2HN**	2.13	1.92	2.34	0.06
**Phe1HD1-HB1**	2.3	2.07	2.53	0.00
**Phe1HN-HB1**	2.885	2.60	3.17	-0.06
**Phe1HD2-HB1**	2.345	2.11	2.58	0.00
**Phe1HN-HB2**	2.8	2.52	3.08	-0.22
**Val2HN-HA**	2.37	2.13	2.61	0.09
**Arg3HN-Val2HA**	3.02	2.72	3.32	0.19
**Val2HA-HB**	2.11	1.90	2.32	0.34
**Val2HN-HB**	2.73	2.46	3.00	0.00
**Val2HA-HG1**	2.49	2.24	2.74	0.27
**Val2HG1-HB**	1.92	1.73	2.51	0.00
**Val2HA-HG2**	2.53	2.28	2.78	0.29
**Val2HG2-HB**	1.91	1.72	2.50	0.00

**Val2HN-HG2**	2.93	2.64	3.22	0.00
**Arg3HA-HB1**	2.4	2.16	2.64	0.38
**Arg3HA-HD1**	2.47	2.22	2.72	0.18
**Arg3HA-HG1**	2.46	2.21	2.71	0.20
**Arg3HA-HG2**	2.35	2.12	2.59	0.26
**Arg3HA-HN**	2.38	2.14	2.62	0.33
**Arg3HA-Gly4HN**	2.31	2.08	2.54	0.23
**Arg3HB1-HN**	2.44	2.20	2.68	0.09
**Arg3HD1-HB2**	2.43	2.19	2.67	0.00
**Arg3HB1-HG1**	1.86	1.67	2.05	0.40
**Arg3HG1-HD1**	2.45	2.21	2.70	0.30
**Arg3HG2-HB1**	2.17	1.95	2.39	0.00
**Arg3HG2-HD1**	1.83	1.65	2.01	0.37
**Arg3HG2-HG1**	2.16	1.94	2.38	-0.18
**Arg3HN-Val2HB**	2.86	2.57	3.15	-0.11
**Val2HN-Arg3HN**	2.69	2.42	2.96	0.07
**Arg3HN-HG1**	2.88	2.59	3.17	0.14
**Gly4HA1-HN**	2.71	2.44	2.98	-0.02
**Gly4HA1-Asp5HN**	3.24	2.92	3.56	0.00
**Gly4HA2-HA1**	1.8	1.62	1.98	0.00
**Gly4HN-HA2**	2.96	2.66	3.26	0.00
**Asp5HA-Phe1HN**	2.2	1.98	2.42	0.00
**Asp5HA-HN**	2.73	2.46	3.00	0.00
**Asp5HN-HB1**	3.06	2.75	3.37	0.00
**Asp5HN-HB2**	3.04	2.74	3.34	-0.10
**Asp5HN-Phe1HN**	3.57	3.21	3.93	0.00

Table S15 : List of NOE's taken for structure calculation of **9a** and the violation observed.

Interaction	NOE Distance	Lower Limit	Upper Limit	Violation
**Phe1HA-HB2**	2.05	1.85	2.26	0.24
**Phe1HA-Val2HN**	2.14	1.93	2.35	0.23
**Val2HA-HN**	2.44	2.20	2.68	0.17
**Val2HA-HB**	2.36	2.12	2.60	0.41
**Val2HB-HN**	2.51	2.26	2.76	0.00
**Val2HB-HG1**	2.19	1.97	2.41	0.09
**Val2HA-HG2**	2.37	2.13	2.61	0.45
**Val2HA-HG1**	2.45	2.21	2.70	0.31
**Val2HB-HG2**	2.10	1.89	2.31	0.17
**Val2HG2-HN**	3.28	2.95	4.01	-0.02
**Arg3HA-HB1**	2.28	2.05	2.51	0.02
**Arg3HD1-HB1**	2.37	2.13	2.61	0.00
**Arg3HB2-HN**	3.97	3.57	4.37	0.00
**Arg3HD2-HG1**	2.20	1.98	2.42	0.07
**Gly4HA1-HN**	2.55	2.30	2.81	0.00
**Gly4HA1-Asp5HN**	3.09	2.78	3.40	-0.43
**Gly4HA2-HA1**	1.91	1.72	2.10	0.00

**Gly4HN-Asp5HN**	3.44	3.10	3.78	0.00
**Asp5HA-Phe1HN**	2.18	1.96	2.40	0.00
**Asp5HA-HB1**	2.65	2.39	2.92	0.13
**Asp5HA-HB2**	2.08	1.87	2.29	0.17
**Asp5HA-HN**	2.55	2.30	2.81	0.12
**Asp5HB2-HB1**	1.87	1.68	2.06	0.00
**Asp5HB1-HN**	2.58	2.32	2.84	0.00
**Phe1HB1-HD2**	2.22	2.00	2.44	0.48
**Phe1HN-HB1**	2.39	2.15	2.63	0.00

Table S16 : List of NOE's taken for structure calculation of **9b** and the violation observed.

Interaction	NOE Distance	Lower Limit	Upper Limit	Violation
**Phe1HA-HB1**	1.73	1.56	1.90	0.40
**Phe1HA-HD1**	2.58	2.32	2.84	-0.17
**Phe1HA-HN**	3.02	2.72	3.32	0.00
**Phe1HA-Val2HN**	2.25	2.03	2.48	0.44
**Phe1HB2-HD1**	2.21	1.99	2.43	0.00
**Val2HN-HA**	3.01	2.71	3.31	0.00
**Val2HA-HB**	2.62	2.36	2.88	0.00
**Val2HN-HB**	2.83	2.55	3.11	0.36
**Val2HA-HG1**	2.72	2.05	3.39	0.00
**Val2HB-HG1**	2.37	1.73	3.01	0.00
**Val2HN-HG1**	3.29	2.56	4.02	0.00
**Val2HA-HG2**	2.66	1.99	3.33	0.01
**Val2HB-HG2**	2.30	1.67	2.93	0.00
**Arg3HA-HG1**	1.90	1.71	2.09	0.15
**Arg3HA-HG2**	1.90	1.71	2.09	0.36
**Arg3HA-HN**	2.94	2.65	3.23	0.00
**Gly4HN-Arg3HA**	2.52	2.27	2.77	0.00
**Arg3HN-Val2HA**	2.90	2.61	3.19	0.38
**Arg3HN-Val2HN**	3.52	3.17	3.87	-0.33
**Gly4HN-HA1**	3.19	2.87	3.51	-0.12
**Gly4HA1-Asp5HN**	2.55	2.30	2.81	-0.01
**Gly4HA1-HA2**	2.01	1.81	2.21	-0.05
**Gly4HN-HA2**	2.51	2.26	2.76	0.10
**Asp5HN-Gly4HA2**	3.24	2.92	3.56	0.00
**Asp5HA-HN**	2.14	1.93	2.35	0.00
**Asp5HN-HB1**	3.08	2.77	3.39	0.00
**Asp5HB1-HB2**	1.86	1.67	2.05	0.00
**Asp5HN-HB2**	3.12	2.81	3.43	0.23
**Phe1HN-HB1**	2.51	2.26	2.76	0.44
**Phe1HD1-Val2HG1**	3.41	2.67	4.15	0.15
**Arg3HA-HB1**	2.61	2.35	2.87	0.16
**Arg3HD1-HB1**	2.63	2.37	2.89	0.00
**Arg3HB2-HG1**	2.23	2.01	2.45	0.00
**Arg3HB1-HG2**	2.42	2.18	2.66	0.00

**Arg3HB1-HN**	2.44	2.20	2.68	0.00
**Arg3HB1-Val2HG2**	3.40	2.66	4.14	0.00
**Arg3HD1-HG2**	1.84	1.66	2.02	0.37

Table S17 : List of NOE's taken for structure calculation of **9c** and the violation observed.

Interaction	NOE Distance	Lower Limit	Upper Limit	Violation
**Phe1HA-HB1**	2.12	1.91	2.33	0.00
**Phe1HA-HB2**	2.14	1.93	2.35	0.48
**Phe1HB1-HB2**	1.84	1.66	2.02	0.00
**Phe1HA-HN**	3.35	3.02	3.69	0.00
**Phe1HA-Val2HN**	2.77	2.49	3.05	0.48
**Phe1HB2-HN**	3.17	2.85	3.49	0.00
**Phe1HA-Arg3HN**	3.48	3.13	3.83	0.00
**Val2HA-Arg3HN**	2.77	2.49	3.05	-0.24
**Val2HN-HA**	2.68	2.41	2.95	0.00
**Val2HB-HG2**	2.07	1.86	2.28	0.21
**Val2HN-HB**	2.51	2.26	2.76	0.31
**Val2HB-HA**	2.28	2.05	2.51	0.30
**Val2HB-HG1**	2.12	1.91	2.33	0.15
**Arg3HA-HB1**	2.23	2.01	2.45	0.06
**Arg3HA-HN**	2.50	2.25	2.75	0.15
**Arg3HA-HG1**	2.42	2.18	2.66	0.48
**Arg3HA-HG2**	2.31	2.08	2.54	0.04
**Arg3HA-HD1**	2.66	2.39	2.93	0.51
**Arg3HN-HB1**	2.56	2.30	2.82	0.34
**Arg3HB1-HG1**	2.18	1.96	2.40	0.42
**Arg3HB1-HG2**	1.91	1.72	2.10	0.42
**Arg3HD1-HB1**	2.39	2.15	2.63	0.46
**Arg3HD1-HG1**	1.82	1.64	2.00	0.50
**Arg3HD1-HG2**	1.59	1.43	1.75	0.49
**Arg3HG1-HN**	2.35	2.12	2.59	0.01
**Arg3HA-Gly4HN**	2.07	1.86	2.28	0.00
**Arg3HB1-Gly4HN**	3.23	2.91	3.55	0.00
**Val2HN-Arg3HN**	2.74	2.47	3.01	0.14
**Arg3HN-Gly4HN**	3.31	2.98	3.64	0.46
**Gly4HN-HA1**	2.83	2.55	3.11	0.00
**Gly4HA1-HA2**	1.81	1.63	1.99	0.00
**Gly4HN-HA2**	2.47	2.22	2.72	0.00
**Gly4HA1-Asp5HN**	2.24	2.02	2.46	0.60
**Asp5HA-HB1**	2.53	2.28	2.78	0.00
**Asp5HA-HB2**	2.21	1.99	2.43	0.36
**Asp5HN-HB1**	2.65	2.39	2.92	0.07
**Asp5HN-HB2**	2.74	2.47	3.01	0.00
**Asp5HB1-HB2**	1.76	1.58	1.94	0.00
**Asp5HA-HN**	2.64	2.38	2.90	0.00
**Asp5HA-Phe1HN**	2.48	2.23	2.73	0.36

Table S18 : List of NOE's taken for structure calculation of **9d** and the violation observed.

Interaction	NOE Distance	Lower Limit	Upper Limit	Violation
**Phe1HD1-HB1**	2.10	1.89	2.31	0.46
**Phe1HN-HB1**	2.89	2.60	3.17	0.46
**Phe1HA-HN**	2.52	2.27	2.77	0.00
**Phe1HD1-HB2**	2.35	2.11	2.58	0.02
**Phe1HN-HB2**	2.80	2.52	3.08	0.35
**Phe1HA-Val2HN**	2.13	1.92	2.34	0.00
**Val2HA-HB**	2.11	1.90	2.32	0.38
**Val2HN-HB**	2.73	2.46	3.00	0.26
**Val2HN-HA**	2.37	2.13	2.61	0.23
**Val2HA-HG1**	2.49	2.24	2.74	0.46
**Val2HG1-HB**	1.92	1.73	2.51	0.00
**Val2HA-HG2**	2.53	2.28	2.78	0.21
**Val2HG2-HB**	1.91	1.72	2.50	0.00
**Val2HN-HG2**	2.93	2.64	3.22	0.41
**Arg3HN-Val2HA**	3.02	2.72	3.32	-0.56
**Arg3HA-HB**	2.40	2.16	2.64	0.29
**Arg3HA-HN**	2.38	2.14	2.62	0.29
**Arg3HB1-HN**	2.44	2.20	2.68	0.00
**Arg3HB1-HG1**	1.86	1.67	2.05	0.34
**Arg3HG1-HD1**	2.45	2.21	2.70	0.09
**Arg3HG2-HB1**	2.17	1.95	2.39	0.13
**Arg3HG2-HD1**	1.83	1.65	2.01	0.37
**Arg3HG2-HG1**	2.16	1.94	2.38	-0.21
**Arg3HN-HG1**	2.88	2.59	3.17	0.27
**Arg3HA-HD1**	2.47	2.22	2.72	0.38
**Arg3HA-HG1**	2.46	2.21	2.71	0.00
**Arg3HA-HG2**	2.35	2.12	2.59	0.42
**Arg3HD1-HB1**	2.43	2.19	2.67	0.37
**Arg3HA-Gly4HN**	2.31	2.08	2.54	0.45
**Gly4HA1-HN**	2.71	2.44	2.98	0.00
**Gly4HA2-HA1**	1.80	1.62	1.98	0.00
**Gly4HN-HA2**	2.96	2.66	3.26	0.00
**Gly4HA1-Asp5HN**	3.24	2.92	3.56	0.00
**Asp5HA-Phe1HN**	2.20	1.98	2.42	0.11
**Gly4HN-Arg3HN**	2.69	2.42	2.96	0.41
**Asp5HN-Gly4HN**	3.57	3.21	3.93	-0.39
**Asp5HA-HN**	2.73	2.46	3.00	0.00
**Asp5HN-HB1**	3.06	2.75	3.37	0.01
**Asp5HN-HB2**	3.04	2.74	3.34	0.00

Table S19 : List of NOE's taken for structure calculation of **10** and the violation observed.

Interaction	NOE Distance	Lower Limit	Upper Limit	Violation
**Phe1HA-HN**	2.52	2.27	2.77	0.14
**Phe1HN-HB1**	2.89	2.60	3.17	0.00

**Phe1HD1-HB2**	2.35	2.11	2.58	0.01
**Phe1HD2-HB1**	2.30	2.07	2.53	0.45
**Phe1HN-HB2**	2.80	2.52	3.08	0.00
**Phe1HA-Val2HN**	2.13	1.92	2.34	0.00
**Val2HA-HB**	2.11	1.90	2.32	0.38
**Val2HN-HB**	2.73	2.46	3.00	0.21
**Val2HN-HA**	2.37	2.13	2.61	0.24
**Val2HA-HG1**	2.49	2.24	2.74	0.00
**Val2HA-HG2**	2.53	2.28	3.18	0.00
**Val2HG1-HB**	1.92	1.73	2.11	0.36
**Val2HG2-HB**	1.91	1.72	2.10	0.37
**Val2HN-HG2**	2.93	2.64	3.22	0.00
**Val2HN-Arg3HN**	2.62	2.42	2.96	0.00
**Arg3HN-Val2HA**	3.02	2.72	3.32	0.16
**Arg3HA-HN**	2.44	2.14	2.62	0.31
**Arg3HA-HG1**	2.35	2.21	2.71	0.00
**Arg3HA-HG2**	2.38	2.12	2.59	0.53
**Arg3HB1-HN**	2.43	2.20	2.68	0.01
**Arg3HB1-HG2**	2.45	1.67	2.05	0.32
**Arg3HD2-HB1**	1.86	2.19	2.67	0.46
**Arg3HG1-HD1**	2.17	2.21	3.10	0.00
**Arg3HN-HG2**	2.71	2.59	3.17	0.29
**Arg3HG2-HB1**	1.83	1.95	2.79	0.00
**Arg3HG2-HD1**	2.16	1.65	2.41	0.16
**Arg3HG2-HG1**	2.88	1.94	3.18	-0.20
**Gly4HA2-HA1**	2.96	1.62	1.98	0.00
**Gly4HA1-HN**	3.24	2.44	2.98	0.00
**Gly4HN-HA2**	2.73	2.66	3.26	0.00
**Gly4HA1-Asp5HN**	1.80	2.92	3.56	0.00
**Asp5HN-Gly4HN**	2.69	3.21	3.93	-0.14
**Asp5HA-HN**	3.06	2.46	3.00	0.00
**Asp5HN-HB1**	3.04	2.75	3.37	0.00
**Asp5HN-HB2**	3.57	2.74	3.34	0.00

Table S20 : List of NOE's taken for structure calculation of **11** and the violation observed.

**Interaction**	**NOE Distance**	**Lower Limit**	**Upper Limit**	**Violation**
**Phe1HA-HN**	3.34	3.01	3.67	-0.11
**Phe1HA-Val2HN**	2.04	1.84	2.24	0.00
**Phe1HA-Arg3HN**	3.47	3.12	3.82	0.00
**Phe1HA-HB1**	2.11	1.90	2.32	0.19
**Phe1HA-HB2**	2.13	1.92	2.34	0.30
**Phe1HB1-HB2**	1.83	1.65	2.01	0.00
**Phe1HB2-HN**	3.16	2.84	3.48	0.00
**Val2HN-HA**	2.67	2.40	2.94	0.00
**Val2HA-Arg3HN**	2.76	2.48	3.04	0.40
**Val2HB-HA**	2.27	2.04	2.50	0.21

**Val2HB-HG1**	2.11	1.90	2.32	0.39
**Val2HB-HG2**	2.06	1.85	2.27	0.34
**Val2HN-HB**	2.5	2.25	2.75	0.00
**Arg3HA-HB1**	2.22	2.00	2.44	0.16
**Arg3HA-HG1**	2.41	2.17	2.65	0.23
**Arg3HA-HG2**	2.3	2.07	2.53	0.00
**Arg3HA-HN**	2.49	2.24	2.74	0.17
**Arg3HA-Gly4HN**	2.06	1.85	2.27	0.00
**Arg3HD1-HB2**	2.38	2.14	2.62	0.00
**Arg3HB2-HG1**	2.17	1.95	2.39	0.11
**Arg3HB1-HG2**	1.9	1.71	2.09	0.34
**Arg3HN-HB2**	2.55	2.30	2.81	0.07
**Arg3HB2-Gly4HN**	3.22	2.90	3.54	0.28
**Arg3HD1-HG1**	1.81	1.63	1.99	0.44
**Arg3HN-Gly4HN**	3.3	2.97	3.63	0.45
**Gly4HN-HA1**	2.82	2.54	3.10	-0.13
**Gly4HA1-Asp5HN**	2.23	2.01	2.45	0.23
**Gly4HA1-HA2**	1.8	1.62	1.98	0.00
**Gly4HN-HA2**	2.46	2.21	2.71	0.18
**Asp5HA-Phe1HN**	2.47	2.22	2.72	0.00
**Asp5HA-HB1**	2.52	2.27	2.77	0.21
**Asp5HA-HB2**	2.2	1.98	2.42	0.16
**Asp5HA-HN**	2.63	2.37	2.89	0.00
**Asp5HN-HB1**	2.64	2.38	2.90	0.00
**Asp5HB1-HB2**	1.75	1.58	1.93	0.00
**Asp5HN-HB2**	2.73	2.46	3.00	0.41
**Phe1HD1-HB1**	2.21	1.99	2.43	0.00
**Phe1HD2-HB2**	2.4	2.16	2.64	0.00
**Phe1HA-HD1**	2.26	2.03	2.49	0.16

Table S21 : Average Φ-Ψ angles of peptide **P-5** obtained over the trajectory.

	Ala1		Ala2		Ala3		Ala4		Ala5	
	Φ	Ψ	Φ	Ψ	Φ	Ψ	Φ	Ψ	Φ	Ψ
**P**	67±9	-115±15	-132±15	47±16	-143±26	-56±13	-144±13	-54±11	-108±13	107±123
**1**	74±9	-81±9	-153±34	17±14	-143±13	-33±14	-146±58	-51±9	-114±9	17±170
**2**	71±9	-101±12	-135±11	28±14	-148±34	57±14	85±8	-50±11	-121±16	144±15
**3**	73±9	78±153	65±8	-83±11	139±14	2±23	-152±11	-71±21	-110±18	64±10
**4**	68±9	-111±14	-134±13	52±12	-150±25	-65±12	-140±14	-38±12	-127±12	124±108
**5**	145±30	-113±19	-110±16	20±19	-146±63	80±19	79±7	-58±14	-135±17	75±21

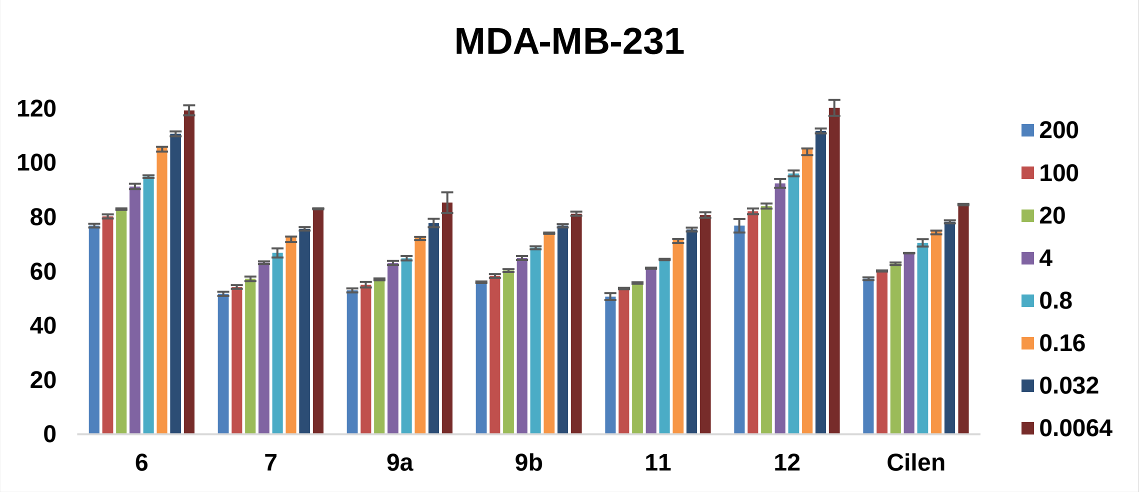

Figure S44 : Proliferation assessed for MDA-MB-231 after 48 hr using MTT Assay (data series are concentration in μM).

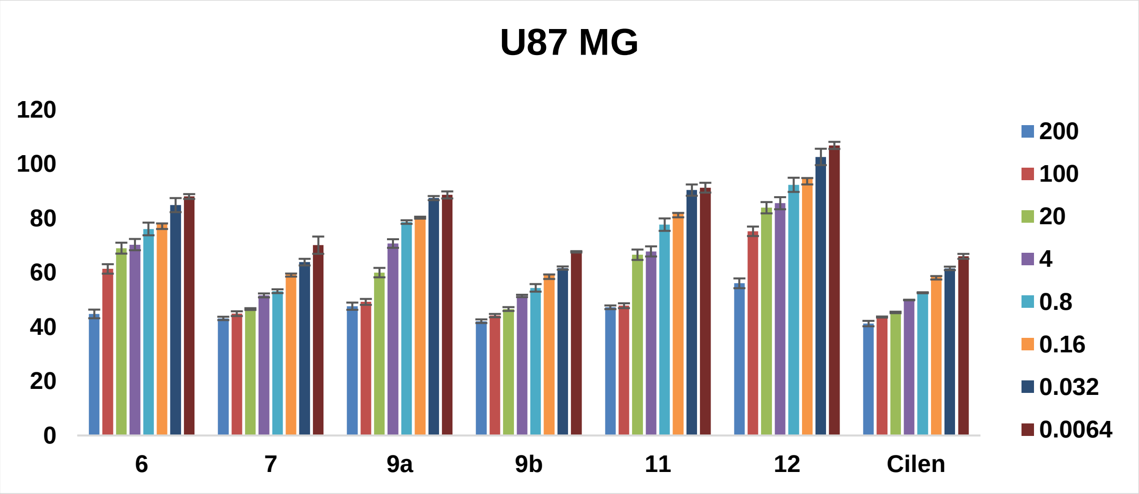

Figure S45 : Proliferation assessed for U-87 MG after 48 hr using MTT Assay (data series are concentration in μM).

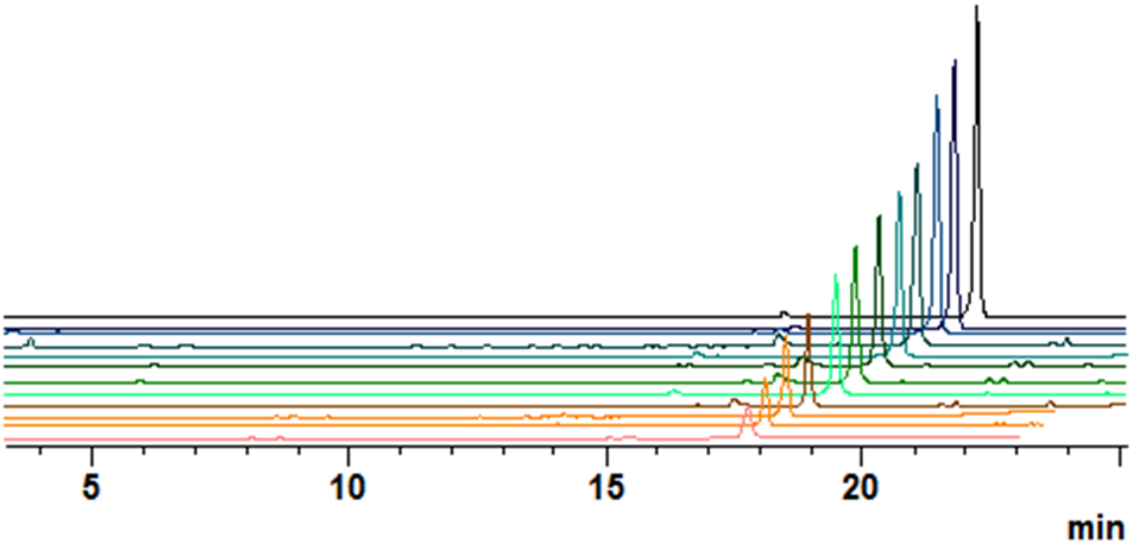

Figure S46 : Representative HPLC chromatogram (extracted at 215 nm) for the assessment of serum stability of **6** at various time points (0, 1, 3, 5. 7, 9, 12, 24, 36, 48, 60 and 72 h).

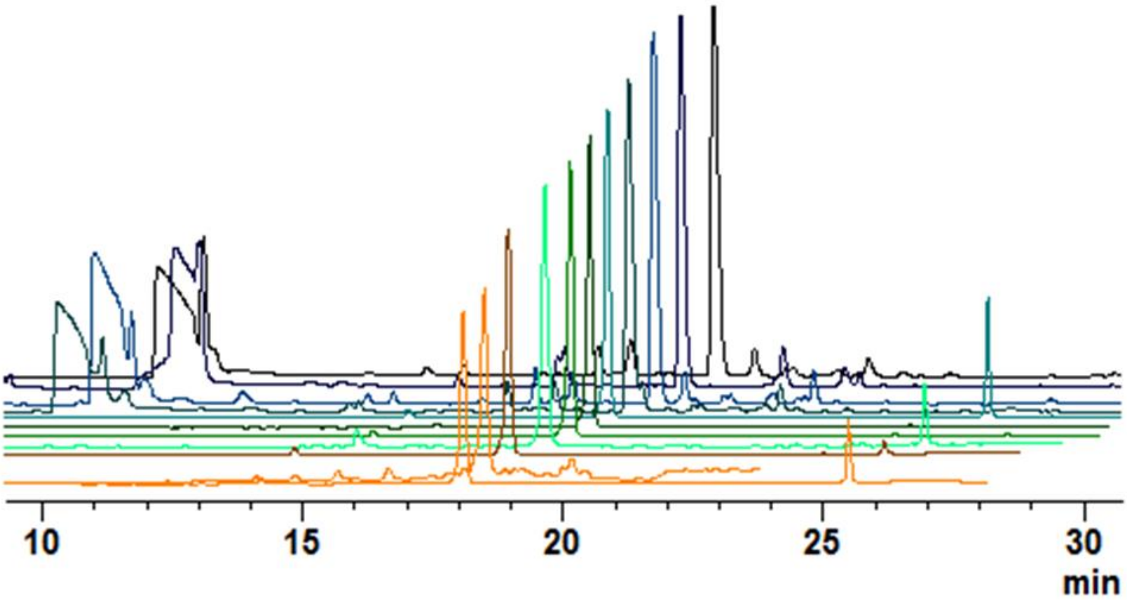

Figure S47 : Representative HPLC chromatogram (extracted at 215 nm for the assessment of serum stability of **7** at various time points (0, 1, 3, 5. 7, 9, 12, 24, 36, 48, 60 and 72 h).

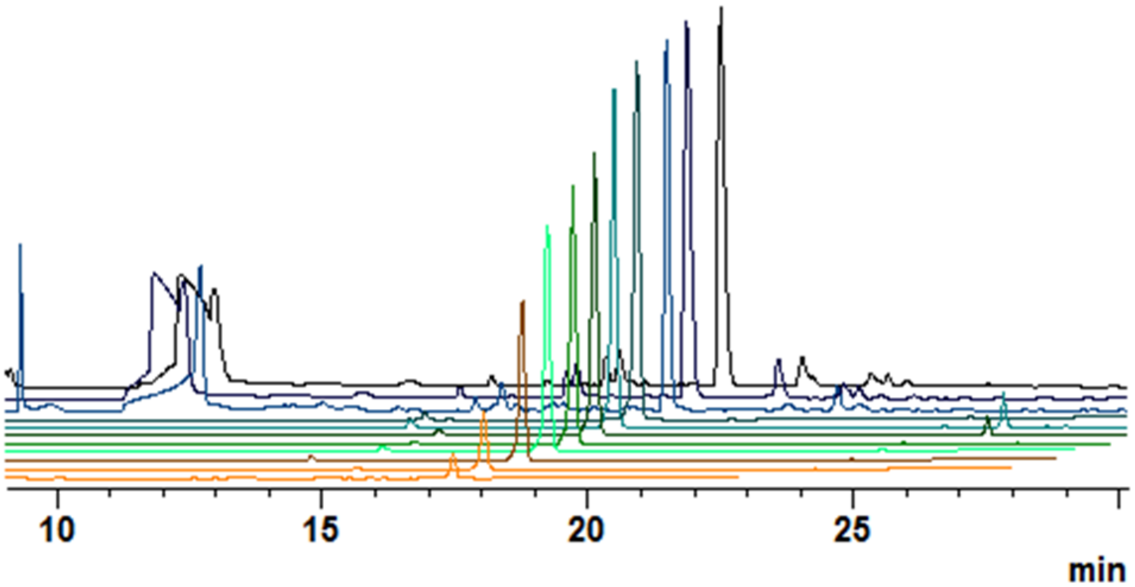

Figure S48 : Representative HPLC chromatogram (extracted at 215 nm) for the assessment of serum stability of **Cilen** at various time points (0, 1, 3, 5. 7, 9, 12, 24, 36, 48, 60 and 72 h).

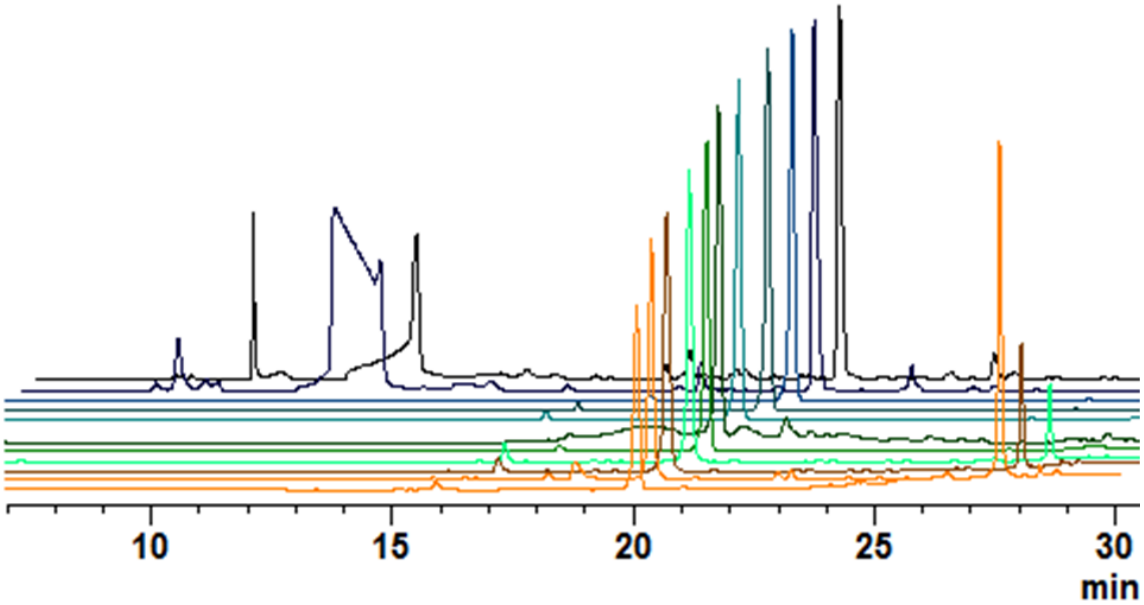

Figure S49 : Representative HPLC chromatogram (extracted at 215 nm) for the assessment of serum stability of **9a** at various time points (0, 1, 3, 5. 7, 9, 12, 24, 36, 48, 60 and 72 h).

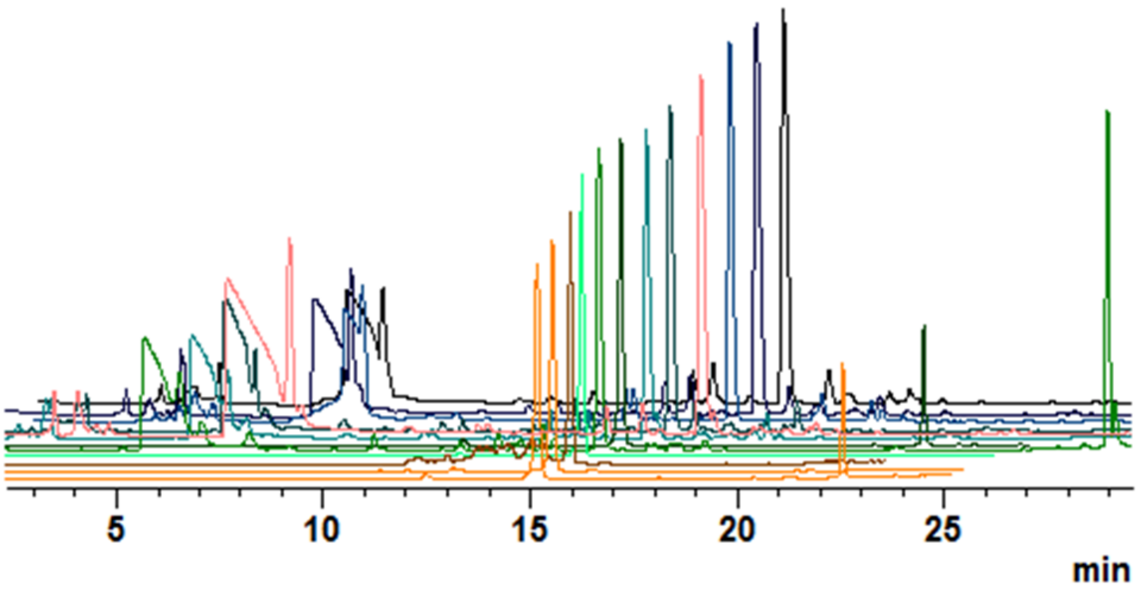

Figure S50 : Representative HPLC chromatogram (extracted at 215 nm) for the assessment of serum stability of **9b** at various time points (0, 1, 3, 5. 7, 9, 12, 24, 36, 48, 60 and 72 h).

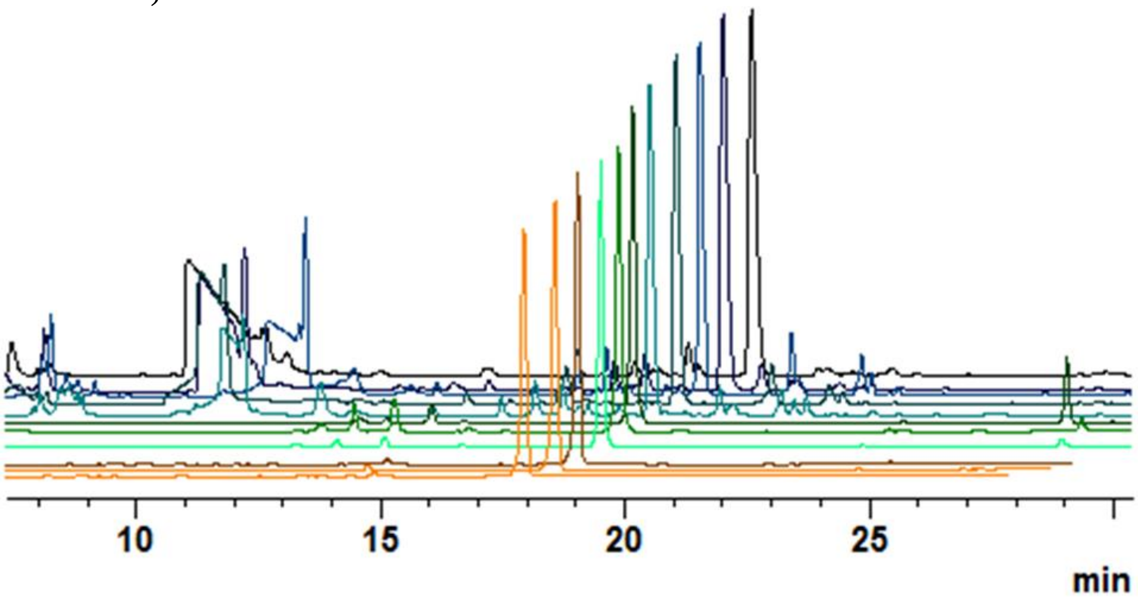

Figure S51 : Representative HPLC chromatogram (extracted at 215 nm) for the assessment of serum stability of **11** at various time points (0, 1, 3, 5. 7, 9, 12, 24, 36, 48, 60 and 72 h).

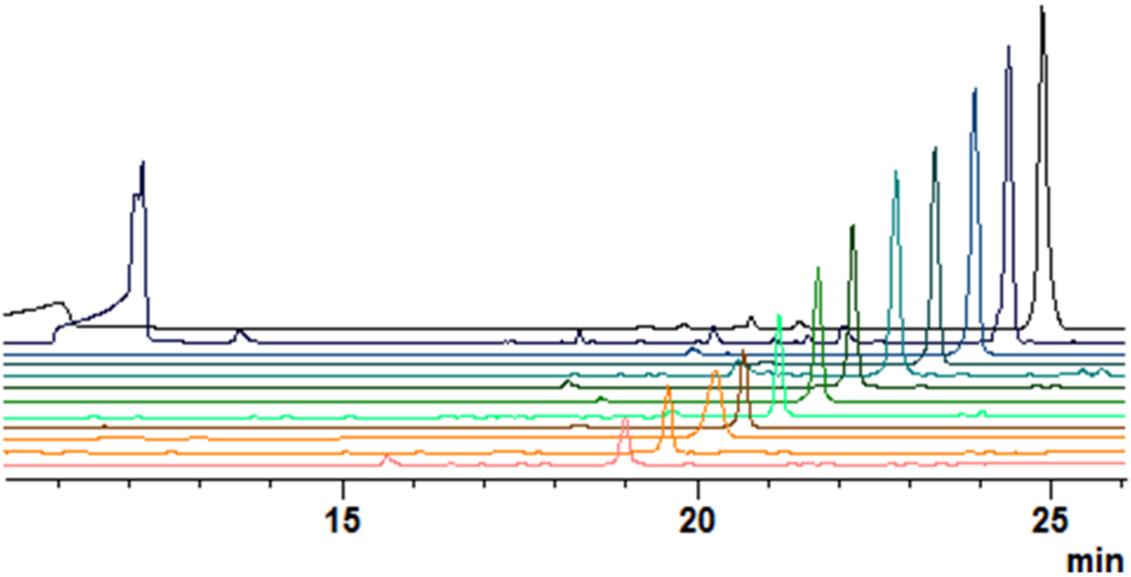

Figure S52 : Representative HPLC chromatogram (extracted at 215 nm) for the assessment of serum stability of **12** at various time points (0, 1, 3, 5. 7, 9, 12, 24, 36, 48, 60 and 72 h).

Results and discussions of in silico experiment

Important Interactions between Integrin and RGD ligands: The Arg and the Asp side chains of the ligand in the protein binding site point to opposite direction. The Arg side chain inserts into a narrow groove at the top of the propeller domain, and its guanidinium group is held in place by a bidentate salt bridge to (α)-Asp218 and by an additional salt bridge to (α)-Asp150. On the opposite side, one of the Asp carboxylate oxygens contacts the metal ion at the MIDAS in βA (which mediates the interaction of Asp with the integrin), while the other carboxylate oxygen forms a hydrogen bond with the backbone amide hydrogen of (β)-Asn215 and (β)-Tyr122. Asp and Arg of the RGD ligands act like an electrostatic clamp, attaching to charged regions of the protein. The pose of RGD ligands in the binding site of integrin influences the distance between C_α (as well as C_β) atoms of Arg and Asp which in turn affects the binding affinity of the ligands (due to conformation dependant loss/gain of some important interactions mediated by the RGD motif).[9] Furthermore, the backbone NH of Asp is involved in a hydrogen bond with the backbone carbonyl oxygen of (β)-Arg216. The glycine residue lies at the interface between the α and β subunits directly on the surface of the protein, making hydrophobic interactions with αV.The most critical hydrophobic contact mediated by Gly residue of the RGD motif appears to be the contact with carbonyl oxygen of (β)-Arg216.[10] In case of **Cilengitide** and αvβ3 complex, the D-Phe of c(-RGDf[NMe]V-) is involved in some hydrophobic interactions.[9]

Comparative analysis of pose and interaction of the ligands with the reference ligand: Although predicted binding affinity as indicated by the docking score is an useful descriptor to identify potential ligands which can bind to a particular receptor but the choice of best docking simulation model should be governed by its agreement with biologically relevant pose of the docked ligands which manifests the probable conformation of the ligand in the protein binding pocket under physiological conditions. The probable biologically relevant binding pose/s of any ligand can be identified when available experimental data are integrated with the in silico results. On a similar note, we have analysed the in silico docking results in the light of the binding pose of **Cilengitide** (the reference RGD motif containing peptide which was a drug candidate) in the crystal structure with PDB ID: 1L5G. Also, the mutagenesis data and structure-activity relationships (SARs) available from earlier literature[9-11] have been considered for analysis of our docking results.

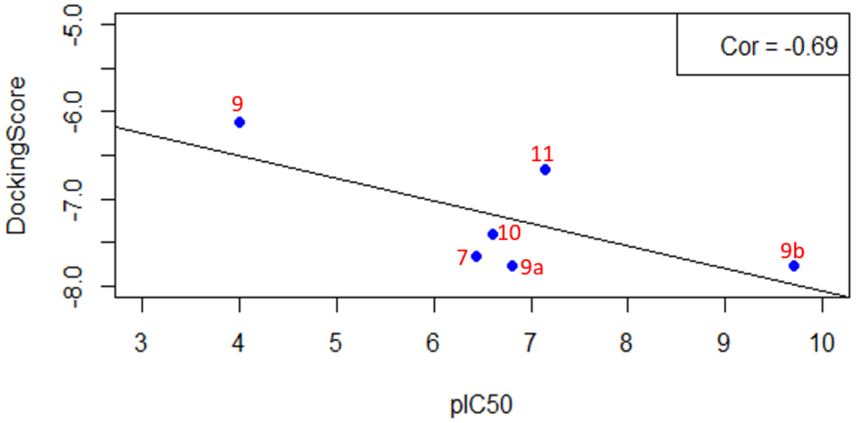

Fig S53: Plot of pIC_{50} Vs SP Docking Score (kcal/mol). Correlation Coefficient (Cor) between pIC_{50} and Docking Score =-0.69 (i.e., 69%, approx 70%; negative sign denotes the two parameters are inversely related). Molecule codes are marked in red against their respective point in the plot.

The default values in Maestro, version 11.1.011 (Schrödinger LLC, 2017-1 release) for detection of different types of interactions (viz., Hydrogen bond, Salt bridge, Aromatic Hydrogen bond etc.) between the ligands and the receptor were used for the analysis (as

discussed in Material and Methods section). Our experimental pIC_{50} for the synthesized peptides (for which docking analysis were carried out) holds a good co-correlation (nearly 70%) with the docking score (**Fig S53**). Higher pIC50 value and lower Docking Score indicates a compound with better potential to be taken forward for further optimization and vice-versa.

The docking analysis results are summarized in **Table S22** and the interpretation of the results are presented below for each molecule.

Re-docked Cilen: With the objective to test the performance of Glide for reproduction of ligand binding pose, the co-crystallized ligand was stripped off from its receptor and re-docked into the same binding pocket of the concerned receptor. It has been found that the re-docked ligand pose closely resembles (RMSD of re-docked pose with respect to co-crystallized Cilen = 0.512Å) the co-crystallized pose of **Cilen** bound to the concerned receptor. As a result, the interactions which are found between the ligand and receptor in the co-crystallized state are retained after re-docking. Superimposition of re-docked **cilen** on to the bound **cilen** in 1L5G shows only slight difference in orientation of Phe residue whereas all the other residues are well superimposed on each other **(Fig. S54)**. Also, upon comparing the experimental pose of **Cilen** bound to protein in 1L5G to its re-docked pose, we see that the stereo-geometrical parameters (which are important for binding and contributing in biological activity) in two cases are very close to each other indicating the presence of all the important interactions between the re-docked ligand and integrin (**Table S22**).

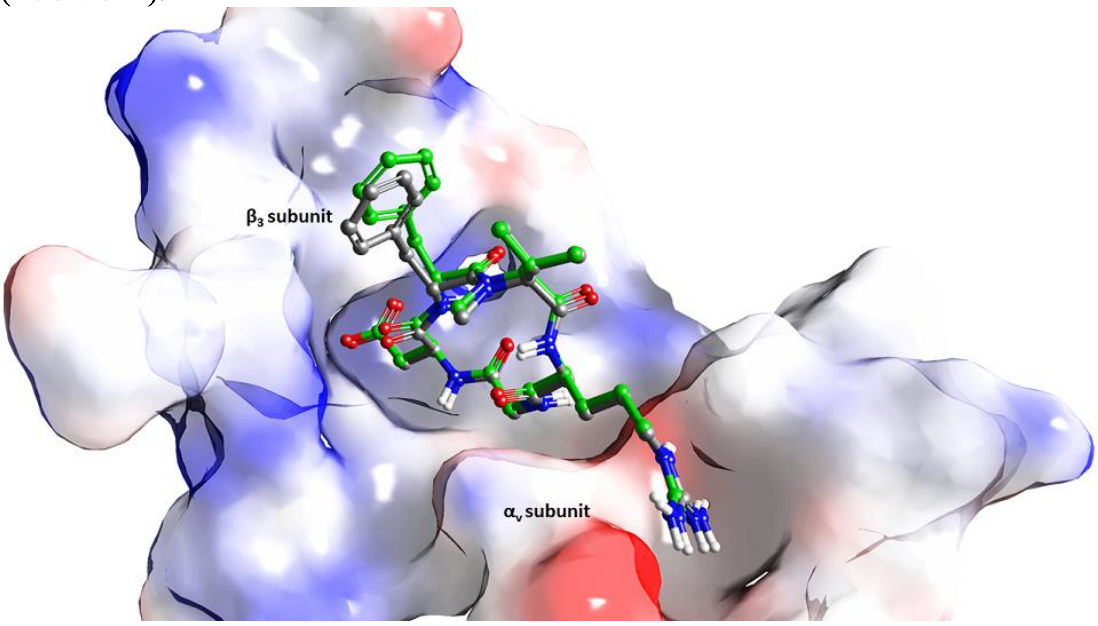

Fig S54: Superimposition of re-docked cilen (grey) on to experimental pose of cilen (green) in the αvβ3 binding site shown with electrostatic surface potential representation.

9b: 9b is the most potent thioamidated peptide in our series as revealed from its experimental IC_{50} against αvβ3. Its IC_{50} against αvβ3 is comparable to that of the reference compound **Cilen**. The pose **(Fig. S55)**, interaction profile **(Fig. 3, S56**) and different stereo-geometrical parameters (**Table S22**) of **9b** also indicate the same. All the important interactions which are observed between **Cilen** and αvβ3 are retained in case of **9b**. In addition, an extra interaction (aromatic hydrogen bond) in case of **9b** has been noted. Although such aromatic hydrogen bonds are weak but they play important role in protein-ligand interactions.[12] One of the aromatic hydrogens of the phenyl ring of Phe residue participates in aromatic hydrogen bonding with carboxylate oxygen (C-H···O) of (β)-Asp126. The distance between C_α (as well as C_β) of Asp and C_α (as well as C_β) of Arg is

very close to that in **Cilen**. As a result, the hydrophobic contacts mediated by Gly are retained; the RMSD of Gly in **9b** with respect to **Cilen** is also low which re-iterates the fact that important hydrophobic contacts mediated by Gly are retained in **9b (Fig. S57)**. Hydrophobic contacts with the β_3 subunit surface similar to that mediated by D-Phe in **Cilen** are also expected in **9b** as indicated by the placement of the Phe ring in L-Phe residue of **9b**.

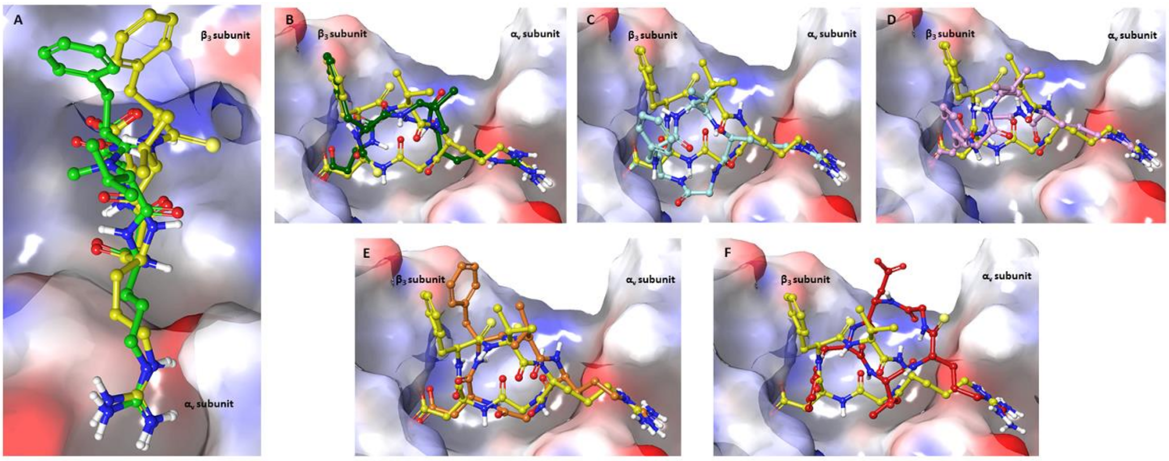

Fig S55: Pose of different peptide ligands in αvβ3 binding site. (A) Overlay of **9b** on to experimental pose of **cilen**. (B) Overlay of docked pose of **11** on to docked pose of **9b**. (C) Overlay of docked pose of **9a** on to docked pose of **9b.** (D) Overlay of docked pose of **10** on to docked pose of **9b**. (E) Overlay of docked pose of **7** on to docked pose of **9b.** (F) Overlay of docked pose of **9** on to docked pose of **9b.** The peptide ligands are shown in ball and stick model (Cilen: light green; 9b: yellow; 11: dark green; 9a: cyan; 10: pink; 7: orange; 9: red) and the protein binding site is sown in electrostatic potential surface representation. The non-polar hydrogens have not been displayed for visual clarity

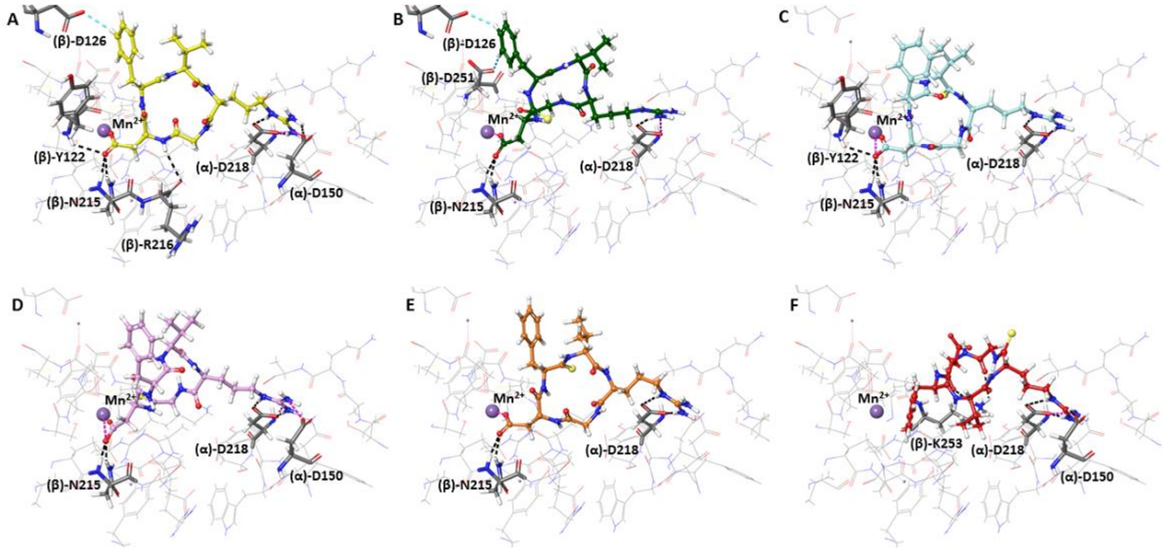

Fig S56: Interaction profile of different peptide ligands in αvβ3 binding site. (A) Interaction of **9b** with binding site residues. (B) Interaction of **11** with binding site residues. (C) Interaction of **9a** with binding site residues. (D) Interaction of **10** with binding site residues. (E) Interaction of **7** with binding site residues. (F) Interaction of **9**

with binding site residues. The peptide ligands are shown in ball and stick model (9b: yellow; 11: dark green; 9a: cyan; 10: pink; 7: orange; 9: red) and the protein residues (in grey colour) within 5Å from the docked ligand in every case are shown (the interacting reidues are shown in tube model while rest of the residues are shown in line model) in the images. The black, magenta and cyan broken lines represent hydrogen bond, salt bridge and aromatic hydrogen bond respectively.

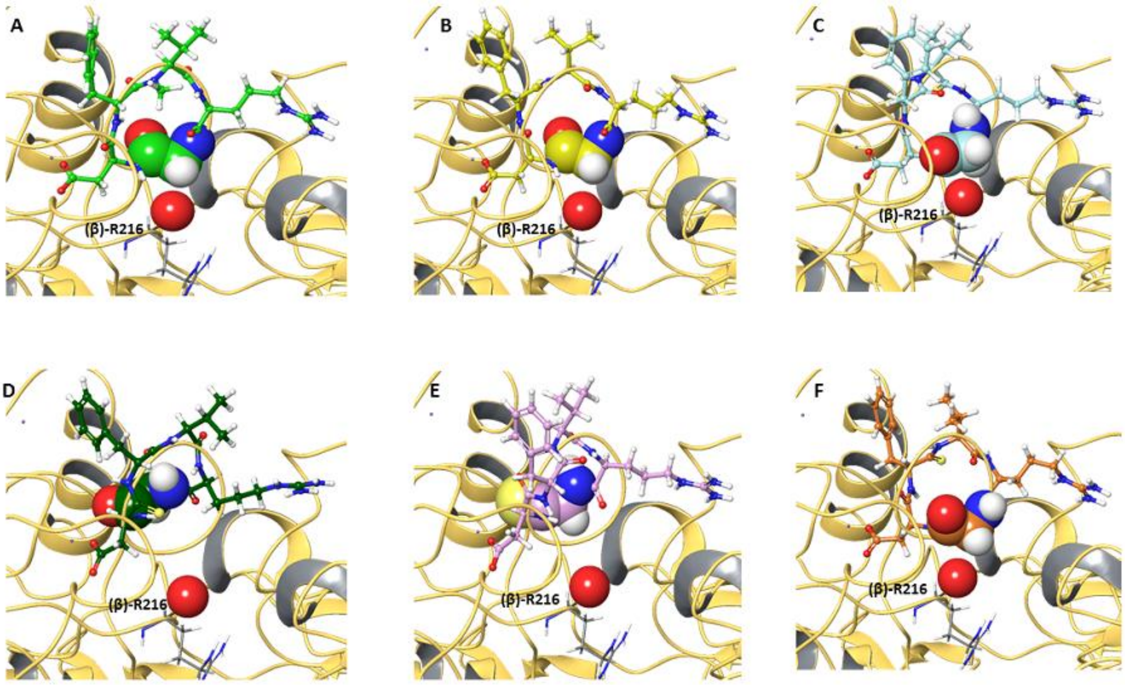

Fig S57: Contact of Gly in RGD motif of different peptide ligands with with carbonyl oxygen of (β)-R216 at the interface of α_v and β_3 subunit. (A) Conatct of **Cilen** with carbonyl oxygen of (β)-R216. (B) Conatct of **9b** with carbonyl oxygen of (β)-R216. (C) Conatct of **9a** with carbonyl oxygen of (β)-R216. (D) Loss of contact (due to larger distance of separation) between Gly of RGD motif in **11** and carbonyl oxygen of (β)-R216. (E) Loss of contact (due to larger distance of separation) between Gly of RGD motif in **10** and carbonyl oxygen of (β)-R216. (F) Conatct of **7** with carbonyl oxygen of (β)-R216. The Gly residue in the peptide ligands are shown in CPK model while rest part of the peptides are shown in ball and stick model (9b: yellow; 11: dark green; 9a: cyan; 10: pink; 7: orange; 9: red). The carbonyl oxygen of (β)-R216 is shown in CPK model but the remaining part of the residue is shown in thin tube model in every case. The protein is represented in cartoon form (faded yellowish orange colour).

11: Experimentally determined IC_{50} value of **11** shows that **11** is poorer than **Cilen** and **9b** which is evident from the visual inspection of the docked pose **(Fig. S55)** of **11** in αvβ3. Some of the stereo-geometrical features **(Table S22)** associated with the docking pose of **11** are close to that of **9b**. However, the distance between C_α of Asp and C_α of Arg is considerably shorter than that in **Cilen** bound to αvβ3. As a result of this Arg and Asp of **11** are not placed optimally in the binding site leading to loss of important interactions **(Fig S56)**. Such deviations from the orientation of RGD motif as observed in **Cilen** bound to αvβ3 resulted in loss of contact between Gly and carbonyl oxygen of (α)-Arg216 **(Fig S57)**. Furthermore, the interaction between Arg and (α)-Asp150 is lost and one of the two hydrogen bonds between Arg and (α)-Asp218 is lost. The hydrogen bonds between the backbone NH of Asp and backbone CO of (β)-Arg216 is lost and also the hydrogen bond between one of the carboxylate oxygens of Asp and backbone amide hydrogen of (β)-

Tyr122 is lost. However, two weak aromatic hydrogen bonds (C-H···O) involving hydrogens attached to aromatic carbons in Phe ring of Phe residue and (β)-Asp251, (β)-Asp126 in the protein binding site are found. The orientation of the Phe ring of D-Phe residue in **11** favours both hydrophobic interactions with the β_3 subunit of the protein as well as aromatic hydrogen bond as seen in **9b (Fig S56)**.

9a: Our experimental data shows that IC_{50} of **9a** is poorer than that of **9b** and **Cilen**. The same is also reflected in the values of different stereo-geometrical parameters **(Table S22)** which are dependent on the pose **(Fig. S55)** of the molecule and in turn affects the interaction profile **(Fig S56)** of the molecule with the receptor. Although we see that most of the stereo-geometrical parameters of **9a** are close to that of **9b** but the distance between C_α of Asp and C_α of Arg in **9a** is considerably shorter than that seen in **Cilen** and **9b** which indicates that Arg and Asp are not optimally placed in the binding site thereby failing to establish some important interactions; the RMSD of Gly residue of **9a** with respect to Gly of **Cilen** bound to αvβ3 is considerably higher than that of **9b**. Also D-residue in **9a** is Asp. All these facts taken together perhaps leads to loss of hydrogen bond between the backbone NH of Asp and backbone CO of (β)-Arg216 but the overall pose of the docked molecule favours contact of Gly with carbonyl oxygen of (β)-Arg216 **(Fig. S57)** which is not seen in **11**. Also, the interaction between Arg of **9a** and (α)-Asp150 is lost. The orientation of the Phe ring of the residue Phe is such that the hydrophobic contacts mediated by D-Phe in **Cilen** and L-Phe in **9b** are somewhat retained in **9a**. However, the orientation of the Phe ring in **9a** does not favour the formation of aromatic hydrogen bond as seen in **9b.**

10: IC_{50} of **10** is quite poor as compared to that of **9b**. However, IC_{50} of **10** is in the in same range as that of **7**. The same is in reflected in the interaction profile **(Fig S56)** and stereo-geometrical features of docked pose **(Fig. S55)** of **10 (Table S22)**. The distance between C_α of Asp and C_α of Arg is considerably shorter than that in **Cilen** bound to αvβ3 which resulted in loss of some important interaction mediated by the RGD motif of the peptide **(**as summarized in **Table S22).** Although the orientation of the Phe ring of the Phe residue is such that the hydrophobic contacts mediated by D-Phe in **Cilen** and L-Phe in **9b** are somewhat retained in **10**. However, the orientation of the Phe ring in **10** does not favour the formation of aromatic hydrogen bond as seen in **9b** and **11 (Fig. S58).** The Gly mediated contact between the peptide and carbonyl oxygen of (β)-Arg216 is also retained **(Fig. S57)**.

7: IC_{50} of **7** shows that it is way poorer than **9b** and the reference ligand (**Cilen**). This is in agreement with the stereo-geometrical features and interaction profile **(Fig S56, Table S22)** of **7**. Although we see that the distance between C_α of Asp and C_α of Arg is very close to that in **Cilen** but at C_β level that distance is little lesser. As a result of which Asp and Arg side chains are not optimally placed to establish all the interactions which are observed in **Cilen** and **9b**. The only difference between **9b** and **7** is change of chirality of Phe residue. In **9b**, L-Phe is present whereas in **7**, D-Phe is present. On comparing the docked pose of **7** and **9b (Fig S55)**, we see that Asp residue of these two molecules is pretty much superimposable on each other. However, as we move counter-clockwise through the backbone of the peptides, we see due to change of chirality, the backbone of the Phe residues moved considerably apart from each other and such shift in the position of the backbone is transmitted to rest of the residues leading to loss of some important interactions. Although Arg mediated side-on interaction with (α)-Asp218 leading to bidentate hydrogen bonding is retained but the end-on interaction with (α)-Asp150 is lost. Backbone mediated interactions of Asp with the backbone of (β)-Tyr122 and (β)-Asn216

are also lost. In **7**, the orientation of Phe ring of Phe residue is such that the hydrophobic interactions mediated by Phe ring are lost and also such orientation does not favour aromatic hydrogen bond. Contact mediated by Gly of RGD motif with carbonyl oxygen of (β)-R216 is retained in **7 (Fig S57)**.

9: The IC_{50} of **9** shows that it is poorest molecule in our series. This is in full agreement with the stereo-geometrical features and interaction profile **(Table S22)** of **9**. No biologically relevant pose **(Fig S55, S56)** was obtained for **9** where the Asp and Arg residue bind to β_3 and α_v subunit respectively as seen in all biologically active RGD ligands against αvβ3**.**

It is interesting to note that in all the biologically active RGD peptides which were subjected to docking analysis, the sulphur introduced into the peptides (during thioamidation) is well accommodated in the binding cavilty of αvβ3 **(Fig S58)**
and might strengthen the protein peptide interaction as discussed in main text

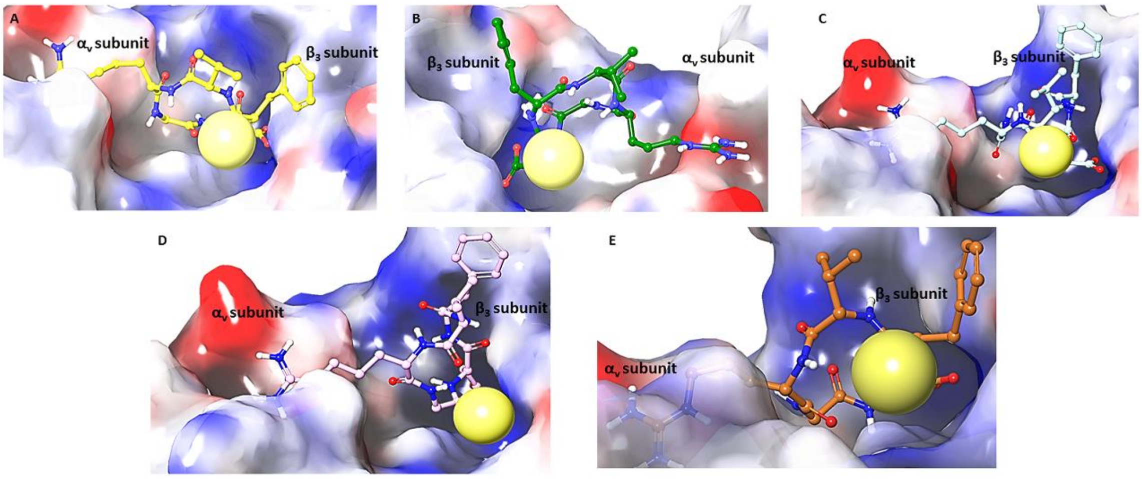

Figure S58: Docked pose of different peptides highlighting position of sulphur atom in the peptide with respect to the protein binding cavity. (A) Docked pose of 9b (B) Docked pose of 11. (C) Docked pose of 9a. (D) Docked pose of 10. (E) Docked pose of 7. The sulphur atom of every peptide has been shown in CPK model and the protein binding cavity has been shown in surface representation to elucidate that the binding cavity of αvβ3 is big enough to accommodate sulphur (which is a bigger atom than oxygen) which has been substituted in place of oxygen during thioamidation. Except sulphur, remaining part of every peptide has been shown in ball and stick model (9b: yellow; 11: dark green; 9a: cyan; 10: pink; 7: orange).

Table S22 : Summarised analysis of in silico experiment's result.

Interactions \ Molecules		Cilen		Synthesized Peptides					
		Bound in 1L5G	Re-docked	**7**	**9**	**9a**	**9b**	**10**	**11**
IC50 (nM)		0.6±0.4	N/A	372±52	>10^5	169±100	0.2±0.1	252±200	72±63
pIC50		9.22	N/A	6.43	4.00	6.80	9.70	6.60	7.14
SP score (kcal/mol)		N/A	-8.76	-7.65	-6.12	-7.77	-7.76	-7.40	-6.66
RMSD at different levels with respect to bound Cilen in 1L5G (Å)	All-atom RMSD	0.00	0.51	7.15	7.76	6.92	7.12	6.77	7.28
	Backbone RMSD	0.00	0.32	5.20	5.89	4.93	4.88	4.88	5.21
	All-atom RMSD of RGD motif	0.00	0.28	8.00	9.01	8.28	8.27	8.21	7.92
	Backbone RMSD of RGD motif	0.00	0.29	3.95	6.91	3.59	3.45	3.88	3.96
	Arg RMSD	0.00	0.28	2.18	3.88	3.77	3.60	2.20	2.72
	Asp RMSD	0.00	0.28	1.48	9.80	2.72	2.82	1.81	2.93
	Gly RMSD	0.00	0.27	1.52	6.62	4.26	2.93	3.13	4.26
	Phe RMSD	0.00	0.96	5.87	6.30	4.34	4.27	4.02	3.95
Distances (Å)	Cα of Gly to C=O of (β)-Arg216	3.28	3.26	3.3	10.72	3.75	3.55	5.62	7.08
	Cα of Asp to Cα of Arg	6.43	6.4	6.41	6.79	5.71	6.46	5.99	5.63
	Cβ of Asp to Cβ of Arg	8.98	8.98	8.31	8.73	7.87	8.86	8.24	6.45
Hydrogen Bond Interaction with Arg	**I** : (α)-D218 (side-on mediated by one H attached to -N^+H_2)	YES	YES	YES	NO	YES	YES	NO	YES
	**II** :(α)-D218 (side-on mediated by one H attached to -$N^{\varepsilon}H$)	YES	YES	YES	YES	YES	YES	YES	NO
	**III** : (α)-D150 (end-on)	YES	YES	NO	YES	NO	YES	NO	NO
	**IV** : (α)-D218 (mediated by second H attached to -N^+H2)	NO	NO	NO	YES	NO	NO	YES	NO

Hydrogen Bond Interaction with Gly#	(β)-K253	NO	NO	NO	YES	NO	NO	NO	NO
Hydrogen Bond Interaction with Asp	Asp & of (β)-N215#	YES	YES	YES	NO	YES	YES	YES	YES
	Asp & (β)- N215_B*	YES	YES	YES	NO	YES	YES	YES	YES
	Asp & (β)-Tyr122#	YES	YES	NO	NO	YES	YES	NO	NO
	Asp# & (β)-R216	YES	YES	NO	NO	NO	YES	NO	NO
	Asp# & (β)-K253#	NO	NO	NO	YES	NO	NO	NO	NO
Aromatic H-bond	H^{ε} of Phe & (β)-D126	NO	NO	NO	NO	NO	YES	NO	NO
	H^{ζ} of F & (β)-D126	NO	NO	NO	NO	NO	NO	NO	YES
	H^{ε} of F & (β)-D251	NO	NO	NO	NO	NO	NO	NO	YES
Salt bridge	R & Asp218_A	YES	YES	YES	YES	YES	YES	YES	YES
	D & Mn^{2+}	YES	YES	YES	NO	YES	YES	YES	YES
	R & Asp150_A	YES	YES	NO	YES	NO	YES	YES	NO
Important Contact	G & Arg 216 [G & (carbonyl O of) Arg 216]	YES	YES	YES	NO	YES	YES	NO	NO

Note: All numerical values are rounded off to two decimal places; # stands for backbone; * stands for side-chain. For interaction cut-off criteria please refer to page number S8.

Mechanistic insight into degradation of Compound 8

A) **RGDfV'** B) C) D) E)

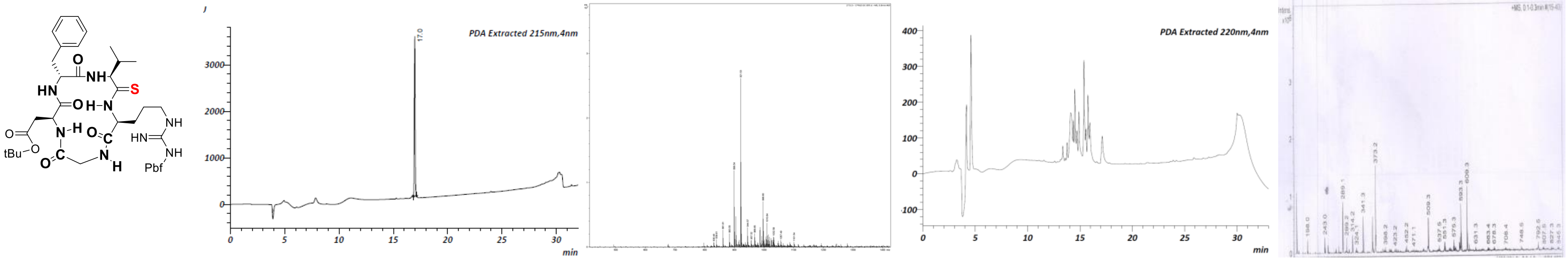

Figure S59 : A) ChemDraw image of cyclo(RGDfV'). B) HPLC Chromatogram of Protected Peptide. C) MS of Protected Peptide. D) HPLC chromatogram after TFA treatment. E) MS of TFA treated crude mixture.

A) **RGDfA'** B) C) D) E)

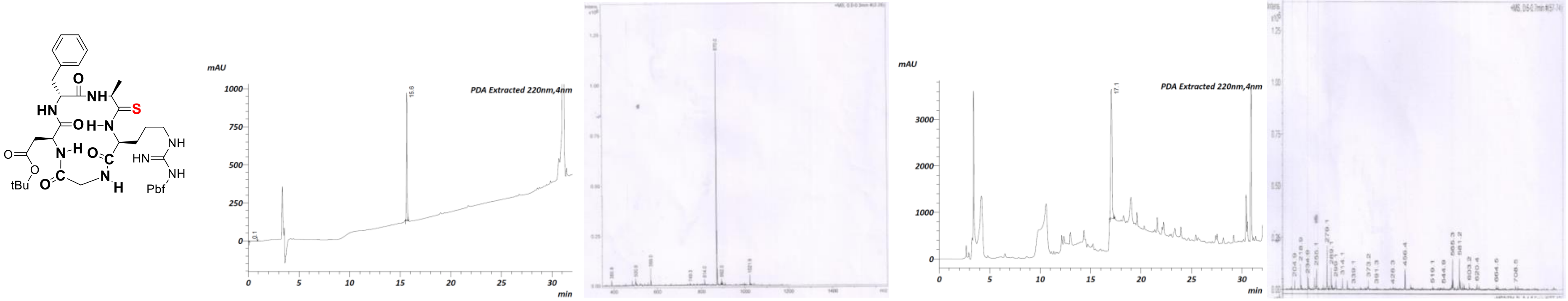

Figure S60 : A) ChemDraw image of cyclo(RGDfA'). B) HPLC Chromatogram of Protected Peptide. C) MS of Protected Peptide. D) HPLC chromatogram after TFA treatment. E) MS of TFA treated crude mixture.

A) **KGDfV'** B) C) D) E)

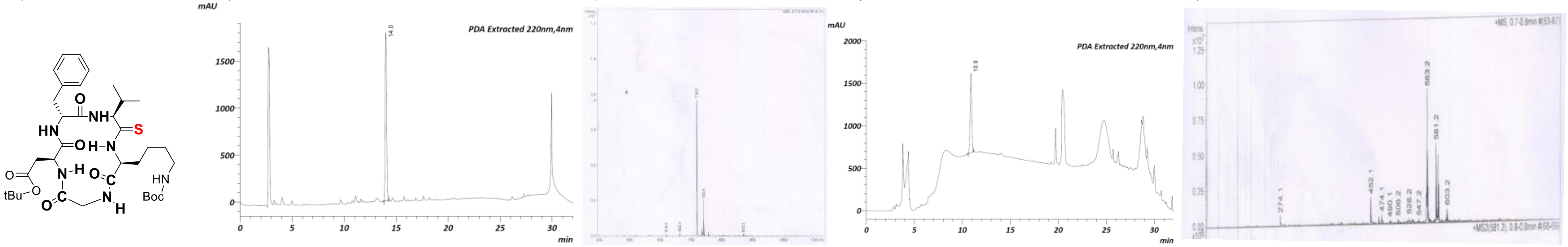

Figure S61 : A) ChemDraw image of cyclo(KGDfV'). B) HPLC Chromatogram of Protected Peptide. C) MS of Protected Peptide. D) HPLC chromatogram after TFA treatment. E) MS of TFA treated crude mixture.

A) (**Nle)GDfV'** B) C) D) E)

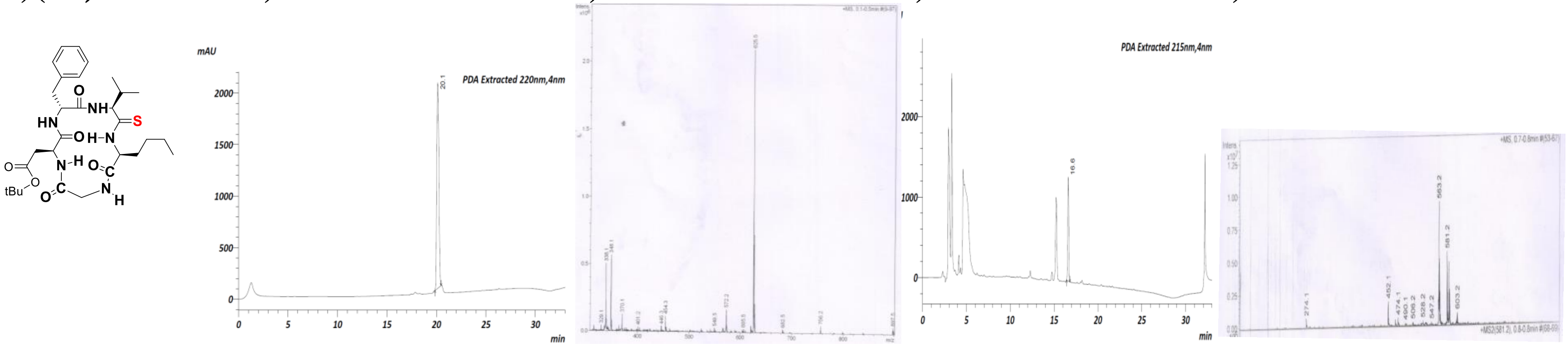

Figure S62 : A) ChemDraw image of cyclo(NleGDfV'). B) HPLC Chromatogram of Protected Peptide. C) MS of Protected Peptide. D) HPLC chromatogram after TFA treatment. E) MS of TFA treated crude mixture.

References

[1] S. Mukherjee, H. Verma, J. Chatterjee, *Org. Lett.* 2015, **17**, 3150.

[2] J. Chatterjee, B. Laufer, H. Kessler, *Nat. Prot.* 2012, **7**, 432.

[3] T. D Goddard, D. G. Kneller, SPARKY 3, University of California, San Francisco.

[4] B. R. Brooks, R. E. Bruccoleri, B. D. Olafson, D. J. States, S. Swaminathan, M. Karplus, *J. Comput. Chem.* 1983, **2**, 187.

[5] H. M. Berman, J. Westbrook, Z. Feng, G. Gilliland, T. N. Bhat, H. Weissig, I. N. Shindyalov, P. E. Bourne, *Nucleic Acids Res.* 2000, **28**, 235.

[6] G. M. Sastry, M. Adzhigirey, T. Day, R. Annabhimoju, W. Sherman, *J. Comput. Aided Mol. Des.* 2013, **27**, 221.

[7] T. A. Halgren, R. B. Murphy, R. A. Friesner, H. S. Beard, L. L. Frye, W. T. Pollard, J. L. Banks, *J. Med. Chem.* 2004, **47**, 1750.

[8] E. Harder, W. Damm, J. Maple, C. Wu, M. Reboul, J. Y. Xiang, L. Wang, D. Lupyan, M. K. Dahlgren, J. L. Knight, J. W. Kaus, D. S. Cerutti, G. Krilov, W. L. Jorgensen, R. Abel, R. A. Friesner, *J. Chem. Theory Comput.* 2016, **12**, 281.

[9] K. E. Gottschalk, H. Kessler, *Angew Chem. Int. Ed.* 2002, **41**, 3767.

[10] J. P. Xiong, T. Stehle, R. Zhang, A. Joachimiak, M. Frech, S. L. Goodman, M. A. Arnaout, *Science* 2002, **296**, 151.

[11] L. Marinelli, A. Lavecchia, K. E. Gottschalk, E. Novellino, H. Kessler, *J. Med. Chem.* 2003, **46**, 4393.

[12] a) V. Aparna, G. Rambabu, S. K. Panigrahi, J. A. R. P. Sarma, G. R. Desiraju, *J. Chem. Inf. Model.* 2005, *45*, 725-738; b) C. Bissantz, B. Kuhn, M. Stahl, *J. Med. Chem.* 2010, **53**, 5061-5084; c) S. K. Panigrahi, G. R. Desiraju, *Proteins* 2007, **67**, 128.
